# Anti-bacterial and anti-biofilm activities of arachidonic acid against the cariogenic bacterium *Streptococcus mutans*

**DOI:** 10.3389/fmicb.2024.1333274

**Published:** 2024-02-26

**Authors:** Manoj Chamlagain, Jieni Hu, Ronit Vogt Sionov, Doron Steinberg

**Affiliations:** ^1^Institute of Biomedical and Oral Research (IBOR), The Faculty of Dental Medicine, The Hebrew University of Jerusalem, Jerusalem, Israel; ^2^Department of Biology, Hong Kong Baptist University, Kowloon, Hong Kong SAR, China; ^3^Lee Kong Chian School of Medicine, Nanyang Technological University, Singapore, Singapore; ^4^School of Life Science and Technology, ShanghaiTech University, Shanghai, China

**Keywords:** arachidonic acid, anti-bacterial, anti-biofilm, antioxidant, efflux pumps, membrane perforation, *Streptococcus mutans*

## Abstract

*Streptococcus mutans* is a Gram-positive, facultative anaerobic bacterium, which causes dental caries after forming biofilms on the tooth surface while producing organic acids that demineralize enamel and dentin. We observed that the polyunsaturated arachidonic acid (AA) (ω-6; 20:4) had an anti-bacterial activity against *S. mutans*, which prompted us to investigate its mechanism of action. The minimum inhibitory concentration (MIC) of AA on *S. mutans* was 25 μg/ml in the presence of 5% CO_2_, while it was reduced to 6.25–12.5 μg/ml in the absence of CO_2_ supplementation. The anti-bacterial action was due to a combination of bactericidal and bacteriostatic effects. The minimum biofilm inhibitory concentration (MBIC) was the same as the MIC, suggesting that part of the anti-biofilm effect was due to the anti-bacterial activity. Gene expression studies showed decreased expression of biofilm-related genes, suggesting that AA also has a specific anti-biofilm effect. Flow cytometric analyses using potentiometric DiOC2(3) dye, fluorescent efflux pump substrates, and live/dead SYTO 9/propidium iodide staining showed that AA leads to immediate membrane hyperpolarization, altered membrane transport and efflux pump activities, and increased membrane permeability with subsequent membrane perforation. High-resolution scanning electron microscopy (HR-SEM) showed remnants of burst bacteria. Furthermore, flow cytometric analysis using the redox probe 2′,7′-dichlorofluorescein diacetate (DCFHDA) showed that AA acts as an antioxidant in a dose-dependent manner. α-Tocopherol, an antioxidant that terminates the radical chain, counteracted the anti-bacterial activity of AA, suggesting that oxidation of AA in bacteria leads to the production of cytotoxic radicals that contribute to bacterial growth arrest and death. Importantly, AA was not toxic to normal Vero epithelial cells even at 100 μg/ml, and it did not cause hemolysis of erythrocytes. In conclusion, our study shows that AA is a potentially safe drug that can be used to reduce the bacterial burden of cariogenic *S. mutans*.

## 1 Introduction

The oral cavity is in constant contact with the external environment which leads to the flourishing of a complex microbiota composed of hundreds of different bacterial and fungal species (Dewhirst et al., 2010; Sharma et al., 2018; Deo and Deshmukh, 2019). The oral environment provides ideal conditions for microbial growth with a temperature of about 37°C, hydrated by saliva with a pH of 6.5–7 which sometimes drops to pH 4–5, and a humidified vapor of exhalant that can reach up to 5% CO_2_ (Sharma et al., 2018; Deo and Deshmukh, 2019). The microbes can colonize both the hard surfaces of the teeth and the soft mucosal tissues (Sharma et al., 2018; Deo and Deshmukh, 2019) where they participate in the development of caries, gingivitis and periodontitis, which are among the most common health problems in both young and elderly people (Abou Neel et al., 2016; Wong et al., 2017). Maintaining a healthy oral condition is also important for the overall well-being of the body, whose function is influenced by the oral microbiome (Sharma et al., 2018; Deo and Deshmukh, 2019).

One of the oral microbes involved in dental caries is the facultative anaerobic Gram-positive *Streptococcus mutans*, which forms strong biofilms on the tooth surface and oral mucosa especially in the presence of sucrose (Forssten et al., 2010; Johansson et al., 2016; Lemos et al., 2019). *S. mutans* can also adhere to the tooth surface independently of sucrose through interaction of adhesion P1 (encoded by *spaP*) with salivary agglutinin (Ahn et al., 2008). Sucrose induces the expression of glycosyltransferases (Gtfs) responsible for the production of exopolysaccharides (EPS) involved in biofilm formation (Koo et al., 2010), but it is also metabolized by *S. mutans* into organic acids such as lactic acid leading to demineralization of the tooth surface and caries (Jijakli and Jensen, 2019; Lemos et al., 2019). Protons expelled from *S. mutans* via the F_1_F_0_-ATPase pump can be trapped by the EPS of biofilms, resulting in localized low pH at the tooth surfaces, which further promotes enamel demineralization (Krzyściak et al., 2014; Guo et al., 2015). Besides being acidogenic, *S. mutans* is acidophilic and has developed mechanisms that allow it to survive under acidic conditions, resulting in a higher prevalence of *S. mutans* and other acidophilic bacteria while preventing the growth of acid-sensitive bacterial strains (Takahashi and Nyvad, 2011; Abranches et al., 2018; Lemos et al., 2019). This vicious circle leads to an imbalanced oral microbiota with further exacerbation of dental caries and accompanying health problems (Abou Neel et al., 2016; Wong et al., 2017; Sharma et al., 2018).

Over the years, various approaches have been used to reduce the oral burden of cariogenic bacteria and to prevent plaque and caries formation (Philip and Suneja, 2023). These include reduced sugar consumption, mechanical removal of plaque, use of antiseptic agents such as chlorhexidine, triclosan, and cetylpyridinium chloride (Marsh, 2010; Qiu et al., 2020; Yazicioglu et al., 2023), detergents such as sodium lauryl sulfate (Yazicioglu et al., 2023), and compounds that strengthen the enamel such as fluoride and casein phosphopeptide-amorphous calcium phosphate (CPP-ACP) (Gurunathan et al., 2012; Giacaman et al., 2023; Philip and Suneja, 2023). In severe cases, treatment with antibiotics is required (Qiu et al., 2020), but these drugs should in general be avoided in order to prevent the development of antibiotic-resistant strains. Various natural plant compounds have also been examined for their usefulness in reducing bacterial load, but their minimum inhibitory concentrations (MICs) are often high, e.g., 1–4 mg/ml for epigallocatechin gallate (EGCG) (Schneider-Rayman et al., 2021; Aragão et al., 2022) and 128–256 μg/ml for curcumin (Song et al., 2012; Ehteshami et al., 2021). EGCG and other polyphenols have the disadvantage of causing dehydration (Oz, 2017), while chlorhexidine and curcumin cause undesirable discoloration of teeth. Moreover, cetylpyridinium chloride, chlorhexidine, triclosan, and amine fluorides can cause toxicities with excessive uses (Dann and Hontela, 2011; Müller et al., 2017; Nasila et al., 2021) and bacteria can develop resistance (Liao et al., 2017; Mao et al., 2020; Huang et al., 2022). Chlorhexidine can cause allergic reactions in rare cases (Fernandes et al., 2019). Therefore, there is still a need to optimize the treatment.

In the course of studying the anti-bacterial and anti-biofilm activities of various compounds against *S. mutans*, we observed that the polyunsaturated arachidonic acid (AA) has anti-bacterial activity against this bacterium. Previous studies have shown that AA has anti-bacterial activity against *Streptococcus pneumoniae* by disrupting fatty acid biosynthesis (Eijkelkamp et al., 2018) and anti-bacterial activity against *Staphylococcus aureus* by causing lipid peroxidation (Beavers et al., 2019). AA also inhibits growth of the unicellular protozoan parasites *Plasmodium falciparum* (Kumaratilake et al., 1992) and the flatworm *Schistosoma mansoni* (Barakat et al., 2015). AA is a natural polyunsaturated fatty acid (ω-6; 20:4) that is ubiquitously present in the human body and serves as a precursor of prostaglandins, thromboxanes and leukotrienes (Tallima and El Ridi, 2018). Most of the cell’s AA is incorporated into membrane phospholipids with a particularly high prevalence in the membranes of macrophages, neutrophils and platelets (Tallima and El Ridi, 2018; Gil-de-Gómez et al., 2020). AA, which is released by activated macrophages upon stimulation (Card et al., 1994; Gil-de-Gómez et al., 2020), is thought to contribute to their anti-bacterial activities (Desbois and Smith, 2010; Das, 2018). Also other fatty acids and their metabolites have been documented to exert anti-bacterial as well as anti-fungal activities (Zheng et al., 2005; Ells et al., 2009; Desbois and Smith, 2010; Huang et al., 2011; Das, 2018; Casillas-Vargas et al., 2021; Wang et al., 2022; Maione et al., 2023), and they are involved in the natural anti-bacterial defense of the skin to maintain a homeostatic skin microbiota (Desbois and Smith, 2010). The anti-bacterial activities of free fatty acids have been associated with altered membrane fluidity, pore formation, impaired nutrient uptake, generation of free radicals, and formation of lipid peroxides (Desbois and Smith, 2010; Das, 2018).

In this article, we have investigated the anti-bacterial and anti-biofilm effects of AA on *S. mutans* and its mode of action, with the aim of evaluating its potential use as a drug to prevent dental caries. We observed that AA has both anti-bacterial and anti-biofilm activities against *S. mutans*, where the minimum inhibitory concentration (MIC) being affected by the environmental CO_2_ levels. We further showed that AA has both bacteriostatic and bactericidal effects which are concentration dependent. AA leads to alterations in the membrane properties, with increased membrane fluidity, membrane perforation, and altered membrane transport of fluorescent efflux pump substrates. AA acts as an antioxidant in *S. mutans*, and its anti-bacterial activity was abrogated by the lipid peroxyl radical scavenger α-tocopherol. Gene expression studies showed that AA reduces the expression of biofilm-related genes indicating a direct anti-biofilm effect besides the anti-bacterial activity. Cytotoxicity studies using normal Vero epithelial cells showed that AA was non-toxic, and a hemolytic assay shows that AA at the concentrations used did not lead to hemolysis. Thus, AA might be considered a safe drug for reducing the oral load of *S. mutans*.

## 2 Materials and methods

### 2.1 Materials

Arachidonic acid (AA) (>99% purity) was purchased from Nu-Check Prep (109 W Main St, Elysian, MN, USA) and dissolved in ethanol (HPLC-grade, Baker, Gliwice, Poland) to a final concentration of 50 mg/ml and stored at −20°C. α-Tocopherol (vitamin E, Sigma, St. Louis, MO, USA), ascorbic acid (vitamin C, Fluka chemika AG, Buchs, Switzerland) and acetyl-L-cysteine (Sigma, St. Louis, MO, USA) solutions were prepared freshly just before use. α-Tocopherol (20 mM) was dissolved in ethanol, while vitamin C (20 mM) and acetyl-L-cysteine (20 mM) were dissolved in sterile double distilled water (DDW). For the AA experiments, the highest concentration of ethanol reached 0.2% and for the combined incubation with α-tocopherol, the highest concentration of ethanol was 0.7%. For all experiments, controls including the same ethanol concentrations (0.03–0.2%) were included, with no significant effect on either planktonic growth or biofilm formation.

### 2.2 Bacterial growth conditions

*Streptococcus mutans* UA159 (ATCC 700610), a clinical isolate from a child with active caries, was cultivated in brain-heart infusion (BHI) broth (HiMedia Laboratories Pvt. Ltd., Maharashtra, India) for planktonic growth and in BHI supplemented with 2% sucrose (BHIS) for biofilm formation (Wolfson et al., 2023). The day before the experiment, 100 μl of a frozen bacterial stock was inoculated in 10 ml BHI and cultured in a humidified incubator supplemented with 5% CO_2_. The morphology of the bacteria was inspected under a light microscope (AXIO Lab.1A, Carl Zeiss Microscopy GmbH. Jena, Germany) at a × 1,000 magnification showing the classical ovoid bacteria held in chains.

### 2.3 Determining the anti-bacterial activity of arachidonic acid

To measure the effect of the compounds on planktonic bacterial growth, an overnight *S. mutans* culture was diluted to an initial optical density (OD_600 nm_) of 0.1 and incubated in the presence of various concentrations of AA for 24 h in 200 μl medium in a 96 flat-bottomed tissue culture grade plate (Corning Incorporation, Kennebunk, ME, USA) at 37°C in a humidified incubator supplemented with 5% CO_2_ (Avraham et al., 2023). At the end of incubation, the OD at 600 nm was measured in a M200 PRO infinite plate reader (Tecan Group Ltd., Männedorf, Switzerland). The % viability was calculated by the following formula: (OD_sample_-OD_background_)/(OD_control_-OD_background_)*100. The minimum inhibitory concentration (MIC) was defined as the lowest concentration of the compound required for no visible bacterial growth after a 24 h incubation. For kinetic studies, the OD_600 nm_ was measured every 30 min at 37°C in a M200 infinite plate reader for 20 h. For checkerboard assay, the bacteria were incubated in various combinations of the two compounds or equal amount of ethanol. Colony forming units (CFUs) were determined at different time points by taking 10 μl of each sample for 10-fold serial dilutions in BHI after vigorous vortexing, and seeding 100 μl of the dilutions onto BHI agar plates, which were incubated for 24 h (Wolfson et al., 2023). The CFU per ml was calculated by multiplying the number of colonies with the dilution factor multiplied with 10 (as 100 μl was seeded out of 1 ml). To measure the ATP content of the bacteria used for the CFU assay, 100 μl of each sample was mixed with 100 μl of BacTiter-Glo microbial cell viability reagent (Promega, Madison, WI, USA) in a μ-clear 96 flat-bottomed white microplate (Greiner Bio-One GmbH, Frickenhausen, Germany), and after 5 min the luminescence was measured in a M200 infinite plate reader (Avraham et al., 2023).

### 2.4 Determining the anti-biofilm activity of arachidonic acid

For biofilm formation, *S. mutans* at an initial OD_600nm_ of 0.1 was incubated in 200 μl BHI with 2% sucrose (BHIS) in the presence of various concentrations of AA for 24 h in a 96 flat-bottomed tissue culture grade plate at 37°C in a humidified incubator supplemented with 5% CO_2_. At the end of incubation, the biofilms were washed twice with phosphate buffered saline (PBS), and either stained with 0.25% crystal violet in ddw (diluted 1:4 from 1% Gram crystal violet (CV) solution, Merck KGaH, Darmstadt, Germany) for 20 min at room temperature, or the metabolic activity was measured by adding 50 μl of a 0.5 mg/ml MTT solution in PBS followed by a 1 h incubation at 37°C as described (Wolfson et al., 2023). The CV stained biofilms were washed twice with DDW, and the absorbance read at 595 nm in a M200 infinite plate reader. The formazan formed by the enzymatic reduction of MTT in the live bacteria was measured by the absorbance at 570 nm in a M200 infinite plate reader. The minimum biofilm inhibitory concentration (BMIC) was defined as the lowest concentration of the compound required for no visible biofilm after a 24 h incubation. To determine the effect of AA on preformed biofilms, *S. mutans* was incubated in BHIS for 6 and 24 h, and the biofilms formed were washed twice with 200 μl PBS and then exposed to AA for 24 h, and the remaining biofilm mass was determined by MTT assay and CV staining as described above.

### 2.5 General description of assays analyzed by flow cytometry

For all assays involving flow cytometry, *S. mutans* was incubated in the absence or presence of various concentrations of AA for 2 h in 1 ml BHI with an initial OD_600 nm_ of 0.3, followed by centrifugation at 5,000 g for 5 min at 4°C, and resuspension in 1 ml of PBS containing the respective probes as indicated below. Data acquisition was performed with a Fortezza flow cytometer (BD Biosciences) using FACSDiva software. Fifty thousand events were collected for each sample, and the collected data were analyzed using FCS Express 7 software. Unstained bacteria served as negative control. The geometric mean of the relative fluorescence intensity (RFI) was calculated by the FCS Express 7 software after setting the same marker for all samples, covering the entire peak(s) of the histograms. Geometric mean is the N^th^ root of the product of N numbers, thus including all collected events.

#### 2.5.1 Membrane permeability assay (SYTO9/PI staining)

For studying membrane permeability, the bacteria were resuspended in 1 ml of PBS containing 3.3 μM SYTO 9 (Molecular Probes, Life Technologies, Carlsbad, CA, USA) and 10 μg/ml propidium iodide (PI) (Sigma, St. Louis, MO, USA), and the fluorescence intensities was measured in a Fortezza flow cytometer using the excitation/emission of 488 nm/520 nm for SYTO 9 and 561 nm/586 nm for PI (Avraham et al., 2023). PI is a positively charged fluorescent dye that binds both DNA and RNA, and it can only penetrate bacteria with damaged membranes. SYTO 9 is another fluorescent dye that binds nucleic acids. In contrast to PI, it can freely enter both live and dead bacteria, and it can be lost from the bacteria if cytoplasmic leakage occurs. Three bacterial populations can be distinguished using this method. Namely, PI^negative^SYTO 9^high^ (live bacteria), PI^high^SYTO 9^high^ (dying bacteria with damaged membrane) and PI^high^SYTO 9^low^ (dead bacteria with damaged membrane and cytoplasmic leakage). The terms “low” and “high” refer to the respective bacterial populations with low and high fluorescence intensity.

#### 2.5.2 Binding of anionic dextran 10,000 to bacterial surface

The binding of the anionic polysaccharide dextran to control and AA-treated bacteria was analyzed by incubating the bacteria in 1 ml of PBS containing 10 μg/ml AlexaFluor^647^-conjugated anionic Dextran 10,000 (Invitrogen, Thermo Fisher Scientific, Eugene, OR, USA) and the fluorescence intensities were measured in a Fortezza flow cytometer using the excitation/emission of 640 nm/670 nm. Dextrans can bind to the bacterial surface (Wu-Yuan et al., 1979) among others by interacting with glucan-binding protein C (Takashima et al., 2015) and glucosyltransferase (Kaseda et al., 2000).

#### 2.5.3 Membrane potential assay

For analyzing alterations in membrane potential, control and AA-treated bacteria were resuspended in 1 ml of PBS containing 30 μM of the potentiometric dye DiOC2(3) (BacLight Membrane Potential Kit, Molecular Probes, Life Technologies, Eugene, OR, USA) (Wolfson et al., 2023). The fluorescence intensities were measured in a Fortezza flow cytometer using the excitation/emission of 488 nm/530 nm for green fluorescence and 488 nm/620 nm for red fluorescence. A relative increase in red fluorescence in comparison to green fluorescence is a sign for membrane hyperpolarization.

#### 2.5.4 DAPI accumulation assay

DAPI accumulation assay was performed by resuspending control and AA-treated bacteria in 1 ml of PBS containing 1 μg/ml of DAPI (4′,6-diamidino-2-phenylindole, Sigma, St. Louis, MO, USA). The fluorescence intensities were measured in a Fortezza flow cytometer using the excitation/emission of 405 nm/450 nm (Banerjee et al., 2021). Since the bacteria are alive, the DAPI staining is a combination of DNA content and efflux pump activities (Button and Robertson, 2001; Kouidhi et al., 2011; Banerjee et al., 2021).

#### 2.5.5 Determination of the DNA content per bacteria

For analyzing the DNA content per bacterium, the control and AA-treated bacteria were fixed in 100% methanol and kept overnight at −20°C. Then the bacteria were rehydrated in PBS, and stained with 1 μg/ml of DAPI. The fluorescence intensities were measured in a Fortezza flow cytometer using the excitation/emission of 405 nm/450 nm (Banerjee et al., 2021). Since the bacteria are fixed, the DAPI fluorescence intensity reflects the DNA content of the bacteria (Button and Robertson, 2001). DAPI is a DNA-specific fluorescent probe (Kapuściński and Yanagi, 1979).

#### 2.5.6 Accumulation assays for various efflux pump substrates

To follow the intracellular dye levels in control and AA-treated bacteria, they were resuspended in 1 ml of PBS containing either of the following efflux pump substrates: 10 μg/ml Nile Red (APExBIO, Houston, TX, USA), 10 μM Rhodamine 6G (Sigma, St. Louis, MO, USA) or 2 μg/ml Ethidium bromide (EtBr, Sigma, St. Louis, MO, USA) for 30 min in PBS. The bacteria were then washed in PBS, and the fluorescence intensities were measured at various time points in a Fortezza flow cytometer using the excitation/emission of 561 nm/635 nm for Nile red, and 488 nm/620 nm for Rhodamine 6G and Ethidium bromide (Banerjee et al., 2021). Nile Red emits red fluorescence when integrated into the membrane, but it is also a substrate for efflux pumps (Bohnert et al., 2010). Ethidium bromide which binds to both DNA and RNA, and Rhodamine 6B which has no known binding partners in bacteria, are fluorescent dyes that are commonly used in measuring efflux pump activities (Paixão et al., 2009; Zeng et al., 2017; Whittle et al., 2019; Lu et al., 2020; Banerjee et al., 2021).

#### 2.5.7 Reactive oxygen species production

For analyzing the effect on ROS production, control and AA-treated bacteria were resuspended in 1 ml of PBS containing 20 μM of the redox probe 2′,7′-dichlorofluorescin diacetate (DCFHDA) (Sigma, St. Louis, MO, USA) for 20 min, and the fluorescence intensities were measured in a Fortezza flow cytometer using the excitation/emission of 488 nm/520 nm.

### 2.6 Morphological studies

To visualize the morphology of planktonic growing cells incubated with AA for 2 h, and biofilms formed in the presence of AA for 24 h, the samples were fixed with 4% glutaraldehyde (Electron Microscopy Sciences, Hatfield, PA, USA) in DDW for 2 h, then gently washed with DDW, and dried before being coated with iridium and visualized by an analytical high-resolution scanning electron microscope (HR-SEM) (Apreo 2 S LoVac, ThermoScientific) at various magnifications. The length of the bacteria was measured by Image J software. For dividing bacteria with a clear septum, the length was measured until the septum.

### 2.7 Spinning disk confocal microscopy (SDCM) of biofilms

Biofilms formed in the presence or absence of AA were stained with 3.3 μM SYTO 9 and 10 μg/ml PI in PBS for 20 min at room temperature, washed in PBS and fixed in 4% paraformaldehyde (Electron Microscopy Sciences, Hatfield, PA, USA) for 30 min at room temperature and then mounted with 50% glycerol before visualization by a spinning disk confocal microscope (Nikon Corporation, Tokyo, Japan) using the 50 μm pinhole, and the 488 nm filter for SYTO 9 and the 561 nm filter for PI (Wolfson et al., 2023). Images were taken at intervals of 2.5 μm from the bottom to the top of the biofilm. Analysis of the relative fluorescence intensities (RFI) in each layer was performed with the NIS-Element software.

### 2.8 Gene expression analysis

*Streptococcus mutans* that have been incubated in the absence or presence of AA for 2 h in 10 ml BHIS with an initial OD_600 nm_ of 0.1, were centrifuged at 5,000 *g* for 10 min at 4°C, resuspended in 1 ml of RNA Protect (Qiagen, GmbH, Hilden, Germany) and kept for 5 min on ice. After removing the RNA protect, the RNA was purified using Tri-Reagent (Sigma, St. Louis, MO, USA) according to the manufacturer’s instruction. The RNA pellet was washed twice with 70% ethanol. The quality of RNA was visualized in 1% agarose gel, and the amount of RNA quantified in a nanodrop (Thermo Scientific) where a ratio of 260/280 around 2 was used as a criterion to proceed to the next steps. The RNA was subjected to DNase I digestion (PerfeCTa DNase I, Quanta Biosciences, Beverly, MA, USA) followed by cDNA synthesis using the AB high-capacity cDNA reverse transcription kit (Applied Biosystems, Life Technologies, Waltham, MA, USA). Ten ng cDNA equivalents were used for each reaction performed in triplicates for each sample. The real-time qPCR reaction was performed with 300 nM forward/reverse primer mix (Supplementary Table S1) and using Power Sybr Green Master Mix (Applied Biosystems, Life Technologies) and the following program: 2 min at 50°C, initial hot start of 10 min at 95°C, and 40 cycles of 15 s at 95°C, 1 min at 60°C, followed by a dissociation curve to ensure uniform PCR product (Banerjee et al., 2021). The relative gene expression was calculated according to the 2^-ΔΔCt^ method (Livak and Schmittgen, 2001) and using *gyrA* as internal standard (Nakano et al., 2007). Each gene of each sample was calculated against each of three controls, and the average of three treated samples, each calculated against the three controls, is presented.

### 2.9 Cytotoxicity assay using Vero epithelial cells

Vero epithelial cells are considered the Gold standard for testing drug cytotoxicity. 4 × 10^5^ Vero cells were seeded in each well of a 96 flat-bottomed tissue-cultured plates in 200 μl DMEM supplemented with 10% heat-inactivated fetal calf serum, 2 mM L-glutamine, sodium pyruvate and penicillin/streptomycin. On the following day, when a confluent monolayer had been formed, the medium was changed to fresh medium containing different concentrations of AA and incubated 24 h at 37°C in a humidified incubator containing 5% CO_2_. The morphology of the cells was inspected by light microscopy, and the relative number of cells was measured by either crystal violet (CV) staining or metabolic MTT assay as described (Avraham et al., 2023).

### 2.10 Hemolytic assay

Defibrinated sheep blood (ThermoScientific, Oxoid Ltd., Hampshire, UK) was washed several times with sterile PBS (Sartorius) until the supernatant became clear. The blood cells were centrifuged at 1,700 *g* for 10 min between each wash. Approximately 10^9^ erythrocytes were incubated with 1 ml of various concentrations of AA in PBS supplemented with 1% bovine serum albumin (BSA, VWR Chemicals, Solon, OH, USA) for 1 h at 37°C. The color intensity of the supernatant was determined at 405 nm in a Multiskan SkyHigh microplate reader (ThermoScientific, Life Technologies Holdings Pte Ltd., Singapore). Percentage hemolysis was calculated in comparison to ddw-exploded erythrocytes, which was set to 100% (Sæbø et al., 2023).

### 2.11 Statistical analysis

The experiments were performed in triplicates and the data are presented as the average ± standard deviation. Statistical analyses were performed by Student’s *T*-test, and differences considered statistically significant when the *p*-value was less than 0.05.

## 3 Results

### 3.1 Anti-bacterial and anti-biofilm activity of arachidonic acid

**Figure 1:**
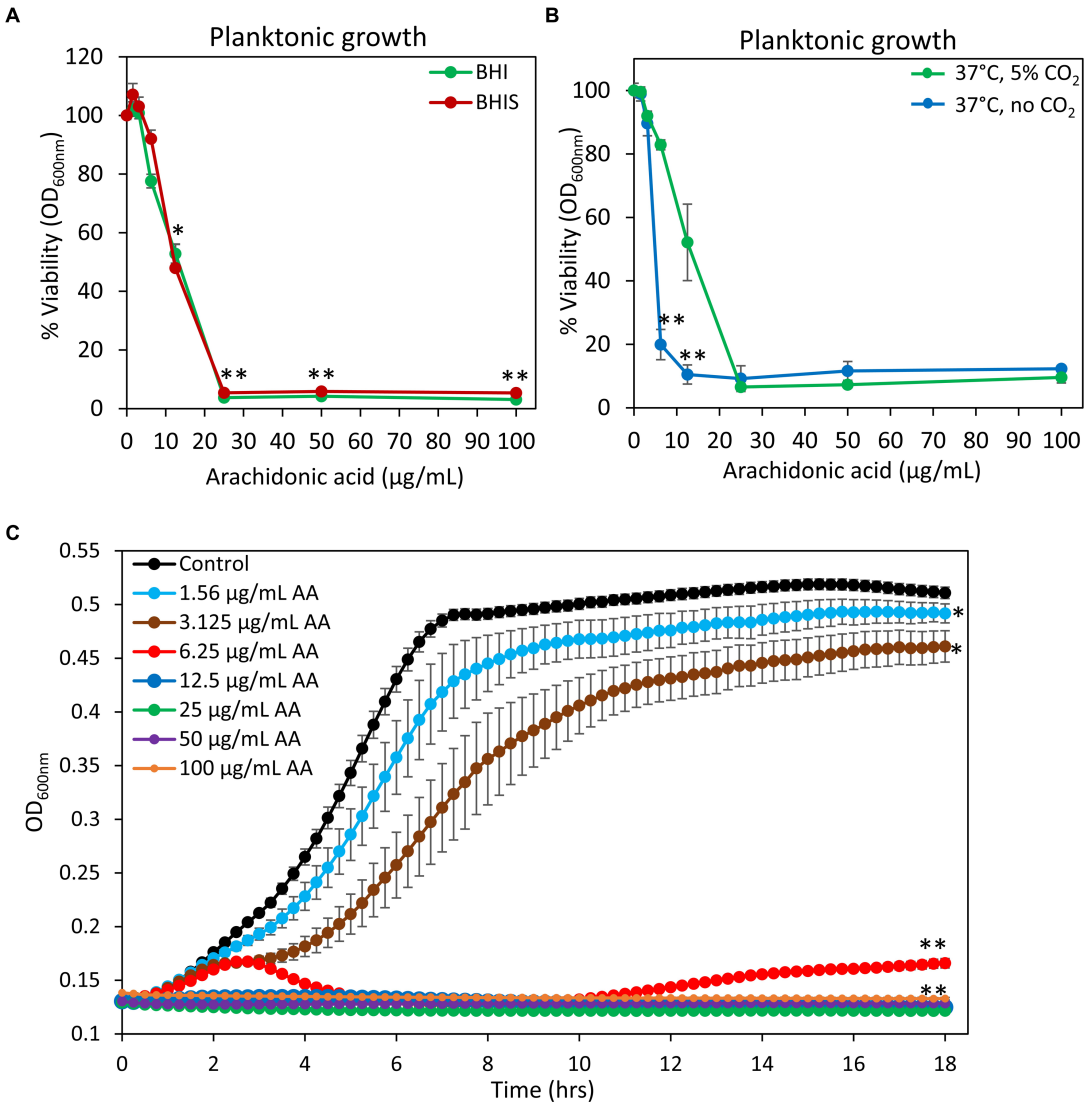
Anti-bacterial activity of arachidonic acid (AA). **(A)** *S. mutans* was incubated with increasing concentrations of AA in BHI growth medium without sucrose (BHI, green line) or BHI with 2% sucrose (BHIS, red line) and the viability was calculated according to the optical density at 600 nm (OD_600 nm_) measured in the culture media after a 24 h incubation. *N* = 3. *p < 0.05 compared to control. **p < 0.001 compared to control. **(B)** The viability of *S. mutans* after a 24 h incubation with various concentrations of AA in the presence (green line) or absence (blue line) of 5% CO_2_. *N* = 3. **p < 0.001 compared to bacteria cultivated in the presence of 5% CO_2_. **(C)** Kinetic growth curve of *S. mutans* incubated with various concentrations of AA as measured in an atmosphere without CO_2_ supplementation. *N* = 3. *p < 0.05 compared to control. **p < 0.001 compared to control. It should be noted that subfigures **(A,B)** are endpoint studies, while subfigure **(C)** is a kinetic study. Parallel incubation with equal dilutions of ethanol (0.03–0.2%) had no effect on bacterial growth.

**Figure 2:**
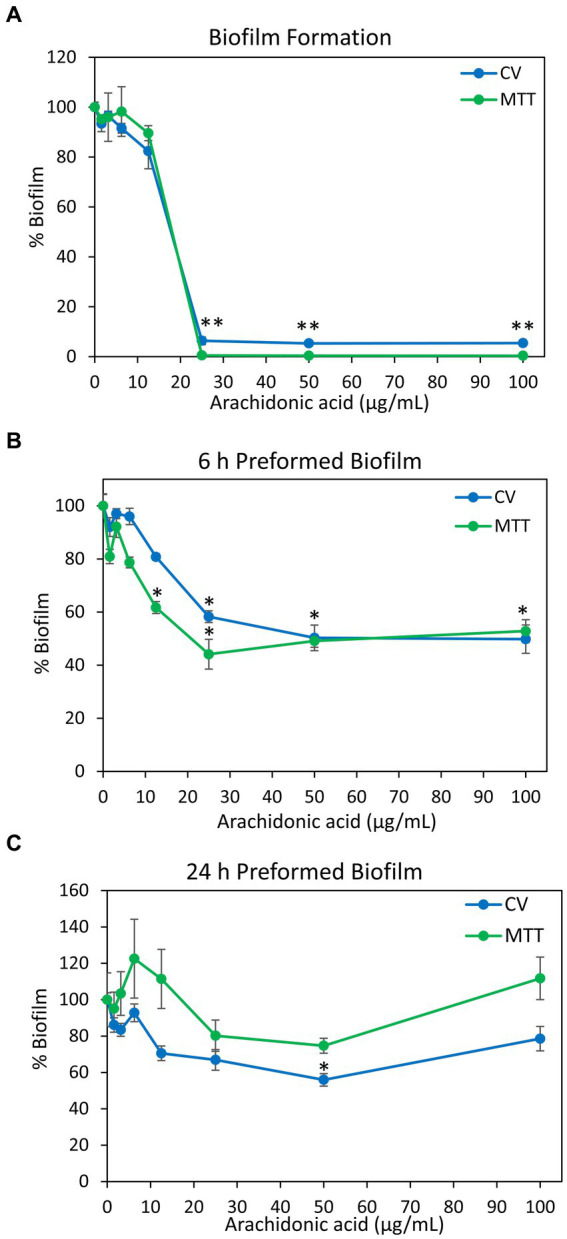
Anti-biofilm activity of arachidonic acid (AA). **(A)** *S. mutans* was incubated with various concentrations of AA in BHI growth medium supplemented with 2% sucrose, and the biofilms formed on the surface was measured after a 24 h incubation by crystal violet (CV) staining (blue line) or MTT metabolic assay (green line). **(B,C)** *S. mutans* was allowed to form biofilm on the surface for 6 h **(B)** and 24 h **(C)** in the presence of 2% sucrose, and then exposed to various concentrations of AA for another 24 h. The resulting biofilms were analyzed by crystal violet (CV) staining (blue line) or MTT metabolic assay (green line). *N* = 3. *p < 0.05 compared to control. **p < 0.001 compared to control. Parallel incubation with equal dilutions of ethanol (0.03–0.2%) had no effect on biofilm metabolic activity or biomass.

Previous studies showed that arachidonoylethanolamine (anandamide) has anti-bacterial and anti-biofilm activities against the cariogenic *S. mutans* (Wolfson et al., 2023). In this context, we were interested in studying whether the polyunsaturated (20:4) ω-6 fatty acid arachidonic acid (AA), which forms the backbone of anandamide (Piomelli, 2014), also possesses such effects. Indeed, we observed that both the planktonic growth and biofilm formation of *S. mutans* were inhibited by AA with the same MIC and MBIC values of 25 μg/ml when incubated at 37°C in an atmosphere of 95% air/5% CO_2_ (Figures 1A, 2A). Equal dilutions of ethanol (0.03–0.2%) had no significant effect on either bacterial growth or biofilm formation. AA had, however, only a partial inhibitory effect (20–40% inhibition) on preformed biofilms (Figures 2B,C), which may be due to the dense biofilm matrix formed by *S. mutans* that prevents drug penetration. The presence of 2% sucrose in the growth medium did not alter the susceptibility of *S. mutans* to AA (Figure 1A). However, when the bacteria were cultured in the absence of CO_2_, they become more susceptible to AA with a MIC value reduced to 6.25–12.5 μg/ml (Figure 1B), suggesting that CO_2_ promotes a salvage pathway that overcomes the anti-bacterial actions of AA. A kinetic study performed without CO_2_ supplementation showed delayed log phase growth when the bacteria were exposed to 1.56 and 3.125 μg/ml AA, while at 6.25 μg/ml there was an initial growth, followed by reduced bacterial density and regained growth after 12 h (Figure 1C). At 12.5 μg/ml and higher concentrations of AA, no growth was observed within the tested period of 18 h (Figure 1C), and even further incubation up to 48 h showed no growth recovery (data not shown). When AA was removed from the growth medium, surviving bacteria regained growth, indicating that the growth inhibition is reversible.

### 3.2 Bactericidal activity of arachidonic acid

**Figure 3:**
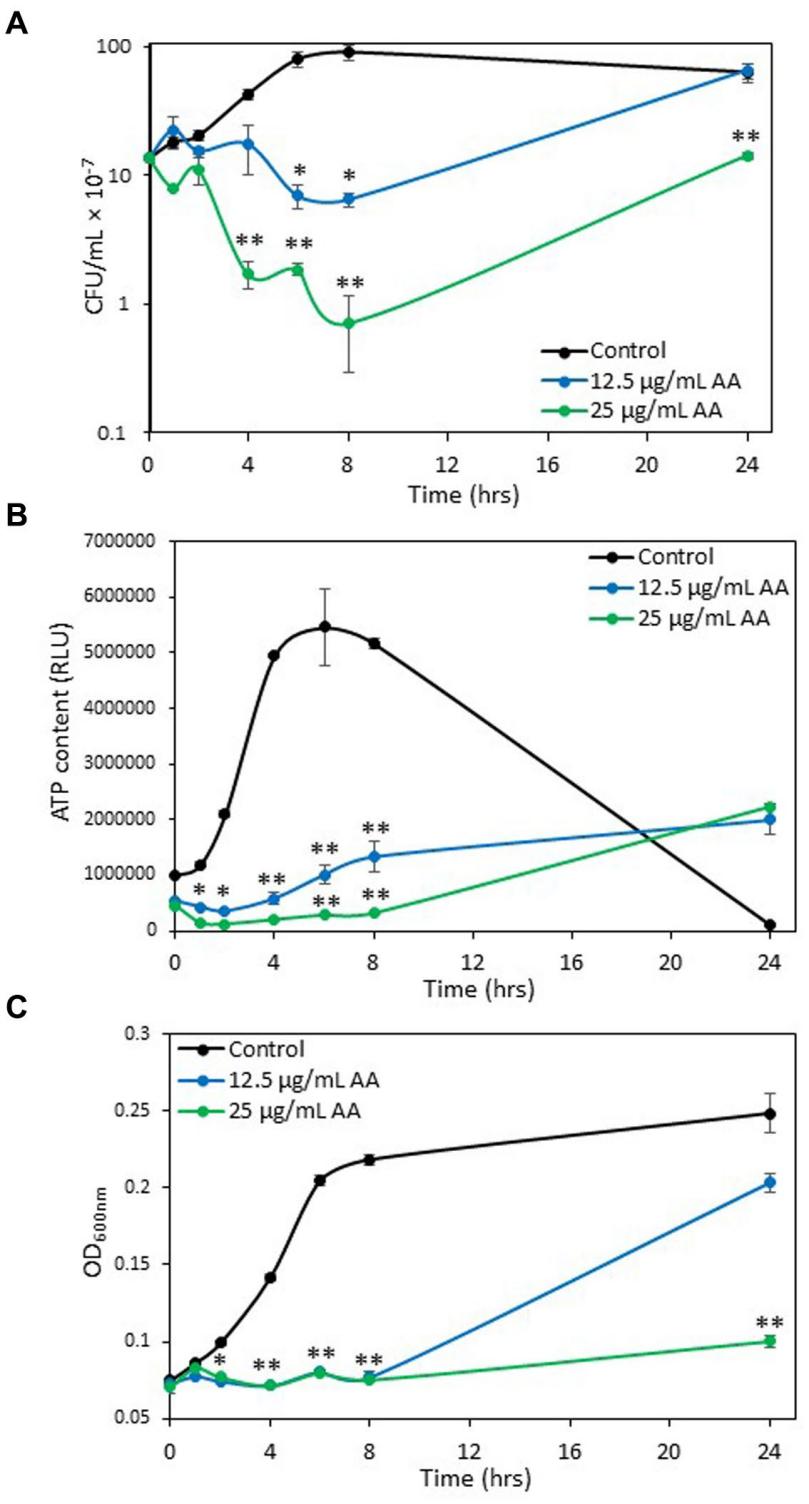
Bacteriostatic and bactericidal effects of arachidonic acid (AA). **(A–C)** Control and AA-treated *S. mutans* were analyzed for colony forming units (CFUs) **(A)**, ATP content **(B),** and optical density **(C)** at various time points following incubation at 37°C in an atmosphere of 95% air/5% CO_2_. The numbers in the *Y*-axis of subfigure **(A)** present the number of CFU × 10^−7^. *N* = 3. *p < 0.05 compared to control. **p < 0.001 compared to control.

To study whether AA has a bacteriostatic or a bactericidal effect, the number of colony forming units (CFUs) were counted at various time points. The control bacteria showed classical growth curve with initial log phase growth at 2 h, reaching a plateau at 8 h, followed by the dead phase (Figure 3A). The dead phase is especially visible by measuring the ATP content of the bacteria that is strongly reduced after 24 h (Figure 3B), despite still high OD (Figure 3C). Bacteria treated with 12.5 μg/ml AA showed an initial doubling of cell number up to 1 h, followed by a decline with a significant 2.5-fold reduction in the cell number between 4 and 6 h, reaching a maximum reduction of 51.9 ± 5.9% at 8 h (Figure 3A). After 24 h, the bacteria treated with 12.5 μg/ml AA had regained growth reaching the number of cells comparable to that observed for control bacteria at 6 h (Figure 3A). This goes along with increased ATP content (Figure 3B) and OD values (Figure 3C) at 24 h. At 25 μg/ml AA, there was a significant reduction in the cell number between 2 and 4 h, reaching a maximum reduction of 94.7 ± 3.1% at 8 h (Figure 3A). There was a small regain of bacterial growth after 24 h, reaching the initial cell number (Figure 3A). The ATP content of 25 μg/ml AA-treated bacteria was also reduced during the first 8 h (85.71 ± 0.1% and 63.44 ± 4.84% reduction after 1 h and 8 h, respectively, in comparison to control at time 0), with an increase after 24 h reaching 2.3–4.0 times the level of the initial value (Figure 3B). The OD remained quite stable during the entire incubation period of 24 h (Figure 3C). These findings indicate that AA has both bacteriostatic and bactericidal effects. The bactericidal effect of AA is observed after a 2–4 h incubation, suggesting that AA does not have an immediate, non-specific cytotoxic effect, but rather acts on essential survival pathways.

### 3.3 Arachidonic acid treatment leads to membrane perforation

**Figure 4:**
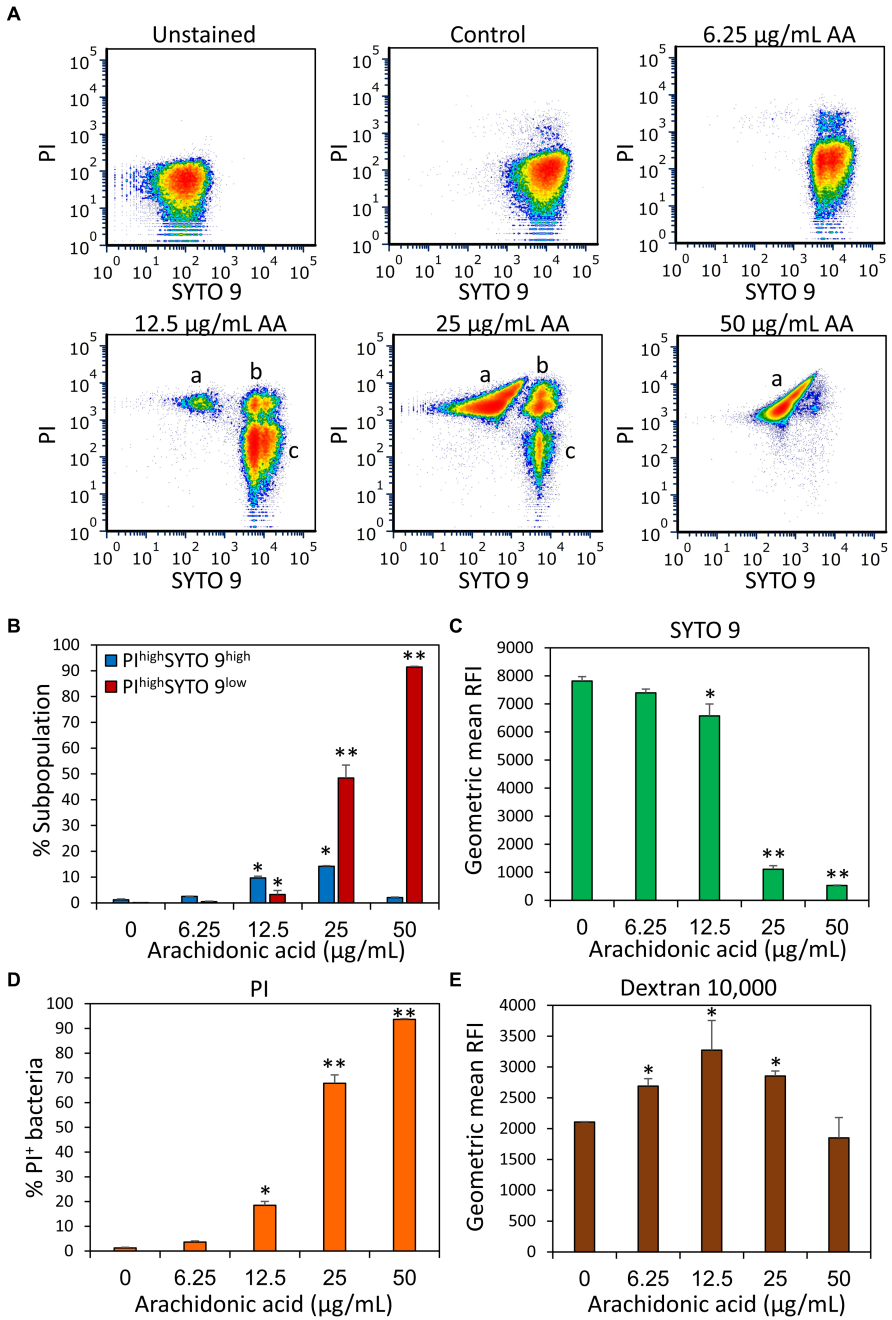
Arachidonic acid (AA) increases the membrane permeability of *S. mutans*. **(A)** Flow cytometry density plots of SYTO 9 and propidium iodide (PI) staining of control bacteria and bacteria treated for 2 h with the indicated concentrations of AA. “a” represents PI^high^ SYTO 9^low^ perforated bacteria with cytoplasmic leakage; “b” represents PI^high^ SYTO 9^high^ bacteria with increased membrane permeability; “c” represents PI^negative^ SYTO 9^high^ live bacteria. The “low” and “high” terminology refers to the respective bacterial populations exhibiting relatively low and high fluorescence intensities. **(B)** The percentage of PI^high^ SYTO 9^high^ and PI^high^ SYTO 9^low^ bacteria upon a 2 h – treatment with various concentrations of AA. **(C–E)** The geometric mean of relative fluorescence intensity (RFI) of SYTO 9 **(C)**, PI **(D)**, and Alexafluor^647^-labeled Dextran 10,000 **(E)**. 50,000 events were collected per sample. *N* = 3. *p < 0.05 compared to control. **p < 0.001 compared to control.

Next, we studied the effect of AA on the membrane properties of *S. mutans*. Live/dead staining with SYTO 9/ propidium iodide (PI) of 2 h-treated *S. mutans* showed a gradual increase in PI uptake with increasing concentrations of AA, indicating that AA increases membrane permeability (Figures 4A,D and Supplementary Figure S1B). This was accompanied by an appearance of a PI^high^ SYTO 9^low^ cell population which represents dying bacteria, reaching cell death values of 48.4 ± 5.0% and 91.4 ± 0.3% for 25 and 50 μg/ml AA, respectively (Figures 4A,B). The SYTO 9^low^ cell population, which is especially observed at 25 and 50 μg/ml AA (Figures 4A,C and Supplementary Figure S1A), is due to a leakage of SYTO 9 out of the perforated bacteria. Simultaneous staining of the bacteria with fluorescent anionic Dextran 10,000 that binds to glucan-binding protein C (Takashima et al., 2015) and glucosyltransferase (Kaseda et al., 2000) as well as positive charged domains on the bacterial surface, showed a bell-shaped dose-dependency, with increasing Dextran 10,000 binding to 6.25–25 μg/ml AA-treated bacteria, while 50 μg/ml AA-treated bacteria showed similar affinity to dextran as control bacteria (Figure 4E and Supplementary Figure S2).

### 3.4 Morphology of arachidonic acid-treated *Streptococcus mutans*

**Figure 5:**
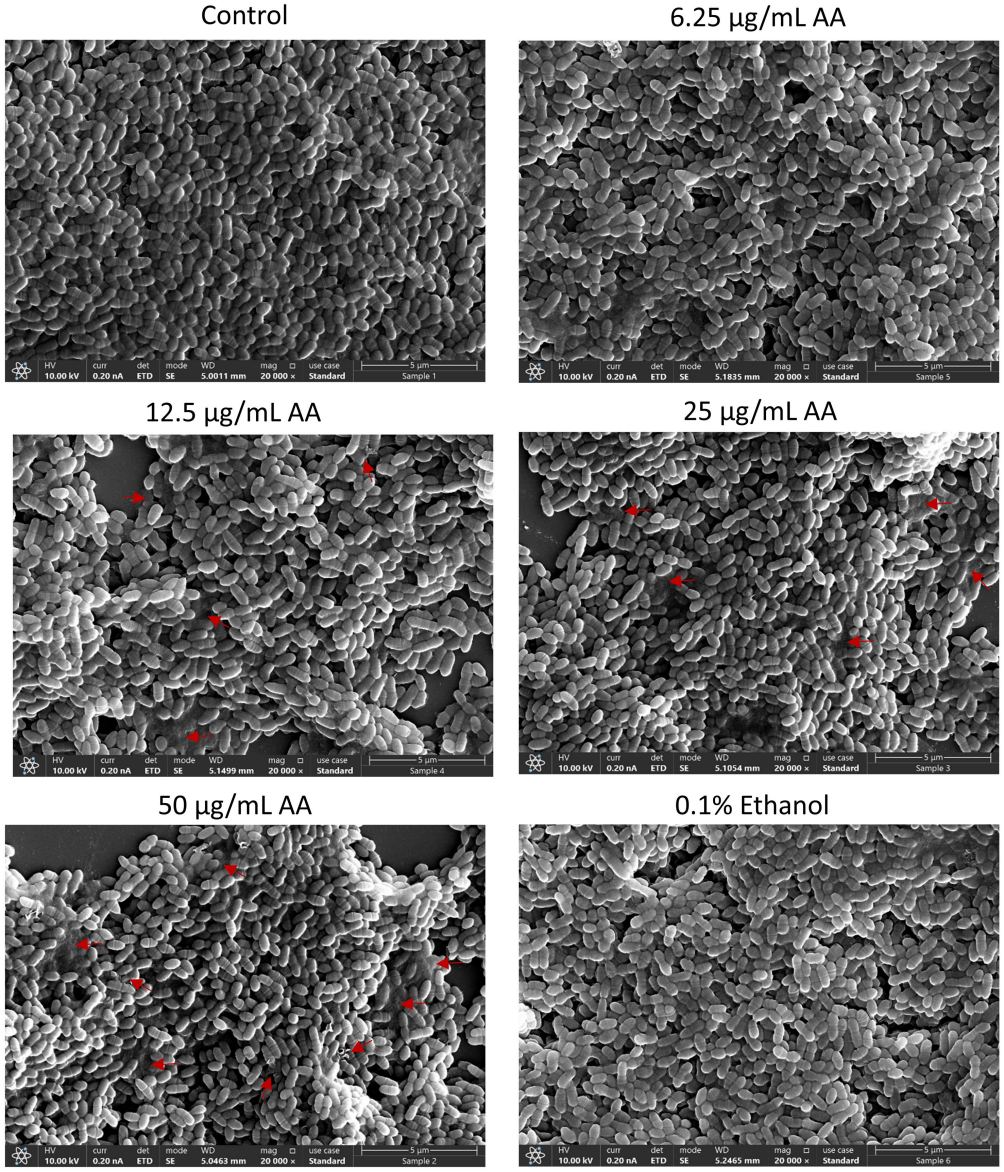
HR-SEM images of control bacteria and bacteria exposed to various concentrations of arachidonic acid (AA) or 0.1% ethanol (negative control) for 2 h. Red arrows point to debris of burst bacteria. Magnification: ×20,000. Uncropped images are presented in Supplementary Figures S3A–F.

**Figure 6:**
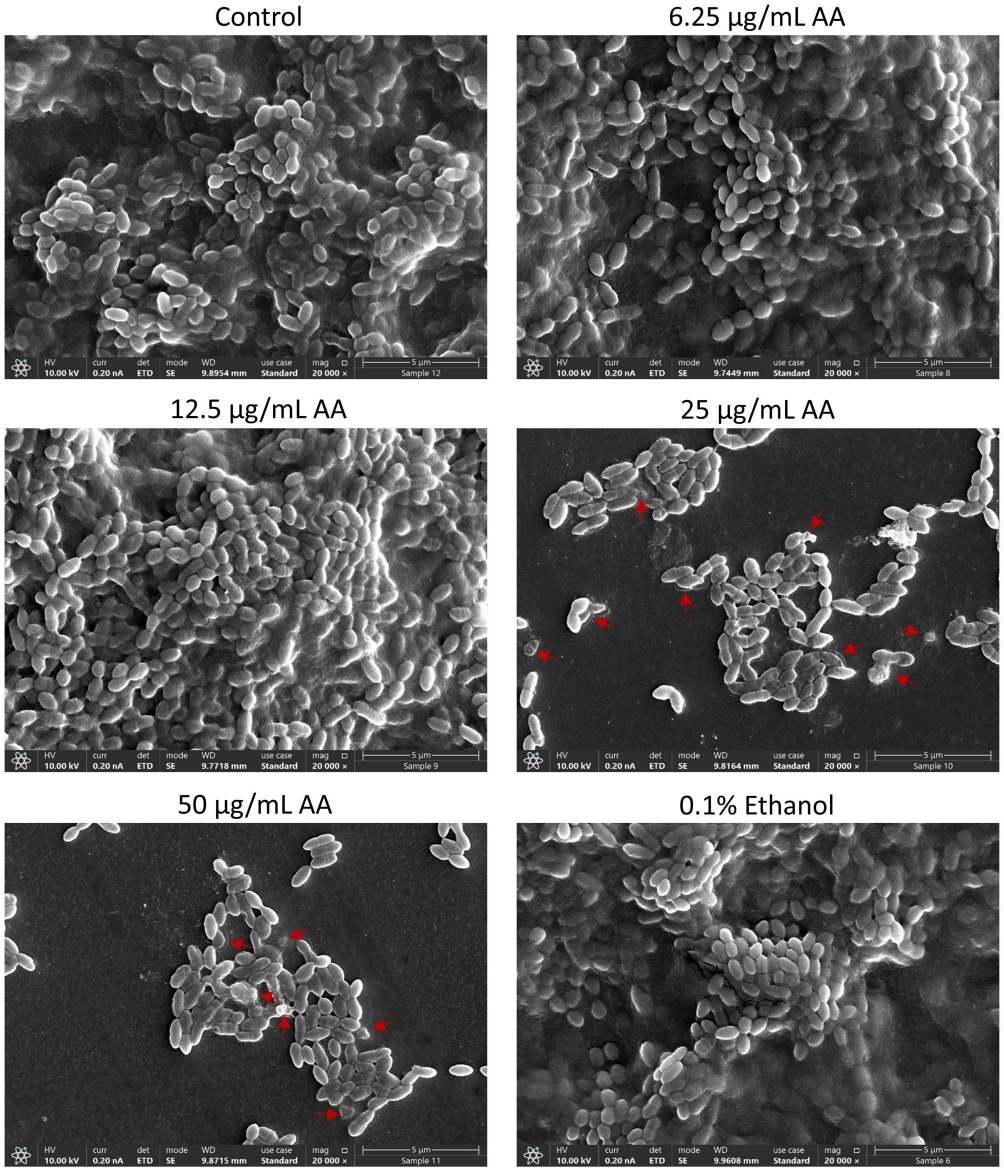
HR-SEM images of biofilms formed by control bacteria and bacteria exposed to various concentrations of arachidonic acid (AA) or 0.1% ethanol (negative control) for 24 h. Extracellular polymeric substances (EPS) appear as a grey diffuse mass that surrounds the bacteria. The EPS can be seen in control, 0.1% ethanol, 6.25 and 12.5 μg/mL AA-treated samples, while absent in samples treated with 25 and 50 μg/mL AA. Red arrows point to swollen bacteria and debris of disintegrated bacteria. Magnification: ×20,000. Lower magnifications of panoramic images are presented in Supplementary Figure S5. Uncropped images are presented in Supplementary Figure S6.

HR-SEM images of planktonic growing bacteria that have been exposed to AA for 2 h showed a similar morphology as the control bacteria (Figure 5 and Supplementary Figure S3A) with classical ovococcoid appearance and double ring-formed structures in the center of the bacteria that seem to represent the FtsZ and MapZ rings of the division plane (Zamakhaeva et al., 2021). Dividing cells can also be seen in bacteria treated with 50 μg/ml AA for 2 h, which frequently appear with more ring-formed structures (Figure 5 and Supplementary Figure S3E). The 50 μg/ml AA-treated bacteria appeared in general with a similar range of cell lengths as the control bacteria with a median cell length of 0.797 μm for 50 μg/ml AA-treated bacteria versus 0.845 μm for control bacteria (Supplementary Figure S4A). Debris and remnants of exploded bacteria can be discerned in the images of 50 μg/ml AA-treated bacteria, suggesting that the bacteria have burst and undergone disintegration (Figure 5 and Supplementary Figure S3). Debris of disintegrated bacteria was also observed in HR-SEM images of bacteria that have managed to adhere to the surface after being exposed to 25 or 50 μg/ml AA for 24 h in growth medium supplemented with 2% sucrose (Figure 6 and Supplementary Figures S5, S6D,E). As expected, the number of bacteria adhered to the surface is strongly reduced when cultured in the presence of 25 and 50 μg/ml AA and only scattered adherent bacterial clusters are observed (Figure 6 and Supplementary Figures S5, S6D,E). While the control biofilms show multilayers of bacteria appearing as hills and valleys filled with a matrix of extracellular polymeric substances (EPS) (Figure 6 and Supplementary Figures S5, S6A), the 25 and 50 μg/ml AA-treated bacteria appear in small scattered monolayer clusters with no apparent extracellular matrix surrounding the bacteria (Figure 6 and Supplementary Figures S5, S6D,E).

The thickness of the biofilm formed in the presence of 12.5 μg/ml AA was strongly reduced, although EPS was produced (Figure 6 and Supplementary Figures S5, S6C). Only some subtle variance in the bacterial lengths were observed between control and AA-treated bacteria (Supplementary Figure S4B). Many of the adherent 25 and 50 μg/ml AA-treated bacteria had pores and altered membrane structures (Figure 6 and Supplementary Figures S6D,E, S7). Many of the 50 μg/ml AA-treated bacteria are held together by shared membrane structures (Supplementary Figure S7).

**Figure 7:**
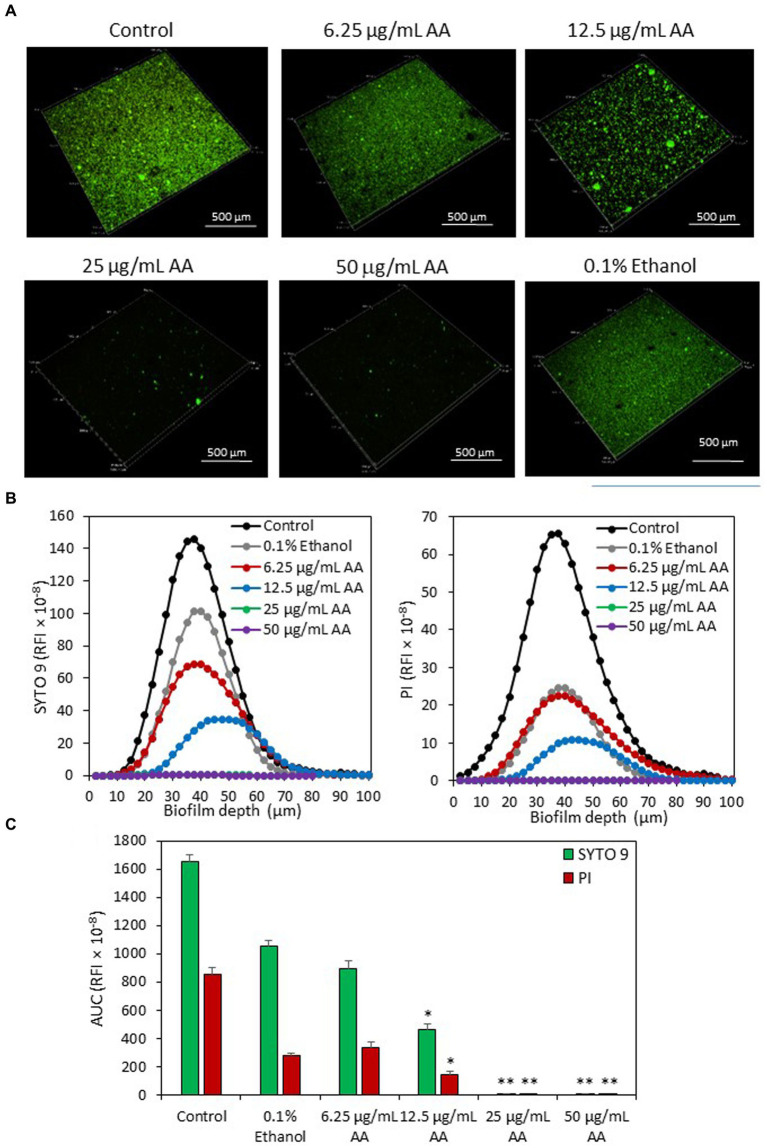
**(A)** 3D Live/dead SYTO 9/PI merged images of *S. mutans* biofilms formed after a 24 h incubation with various concentrations of AA as observed by spinning disk confocal microscopy (SDCM). **(B)** The relative fluorescence intensities (RFI) of SYTO 9 and PI of each biofilm layer of each sample. The graphs represent the average measurements done on 10–13 images captured from each treatment group performed in triplicates. **(C)** The average area under the curve (AUC) of the samples analyzed in **(B)**. The numbers in the *Y*-axis of subfigures **(B,C)** present the relative fluorescence intensity (RFI) × 10^−8^. *p < 0.05 compared to control. **p < 0.001 compared to control.

Live/dead staining of biofilms with SYTO 9/PI confirms a strong reduction (>99%) of biofilm mass when bacteria were cultured in the presence of 25 and 50 μg/ml AA, and a partial reduction (72 ± 3%) of biofilm in the presence of 12.5 μg/ml AA (Figure 7). Both SYTO 9 staining which represents live and dead bacteria, and PI staining that represents dead bacteria, were reduced with increasing concentrations of AA (Figure 7). The reduced PI staining goes along with the reduced number of adherent bacteria in the AA-treated samples (Figure 7). There was no increase in the PI/SYTO 9 ratio in biofilms formed in the presence of AA, which may be due to the fact that only bacteria that survived AA treatment can adhere to the surface.

### 3.5 Arachidonic acid induces immediate membrane hyperpolarization in *Streptococcus mutans*

**Figure 8:**
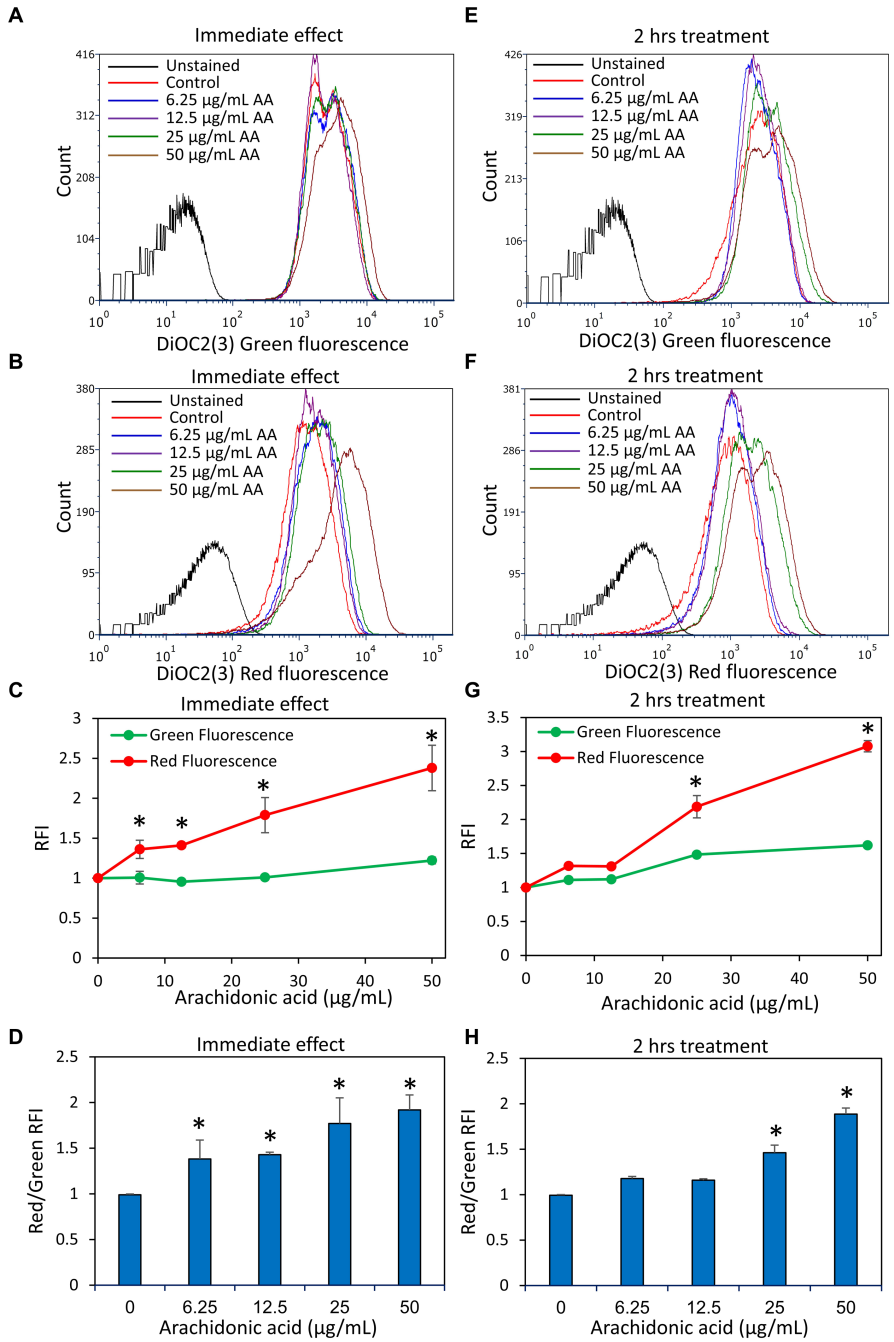
Arachidonic acid (AA) causes an immediate membrane hyperpolarization in *S. mutans*. **(A,B)** *S. mutans* was exposed to the indicated concentrations of AA in PBS at room temperature, and the membrane potential was measured by exposing the bacteria to the potentiometric dye DiOC2(3). The green fluorescence of DiOC2(3) is an indication of the amount of dye taken up by the bacteria, while a relative increase in red fluorescence indicates membrane hyperpolarization. **(C)** The relative fluorescence intensities (RFIs) of green and red fluorescence of samples from **(A,B)**. *N* = 3. **(D)** The ratio of red to green fluorescence intensities of samples from **(A,B)**. *N* = 3. **(E,F)** *S. mutans* was incubated with the indicated concentrations of AA in BHI for 2 h at 37°C, and then the membrane potential was measured by DiOC2(3). **(G)** The relative fluorescence intensities (RFIs) of green and red fluorescence of samples from **(E,F)**. *N* = 3. **(H)** The ratio of red to green fluorescence intensities of samples from **(E,F).** *N* = 3. *p < 0.05 compared to control.

Another parameter important for bacterial cell viability is the maintenance of a dynamic and homeostatic membrane polarization which is an important energy source for the bacteria (Benarroch and Asally, 2020). We therefore studied the immediate effect of AA on the membrane potential of *S. mutans* within 30 min after exposing the bacteria to AA, and the late effect after a 2 h incubation. We observed that AA induced an immediate membrane hyperpolarization in a dose-dependent manner, that was still high after a 2 h incubation with the higher AA doses (25 and 50 μg/ml) (Figure 8). The lower AA doses (6.25 and 12.5 μg/ml) also caused an immediate membrane hyperpolarization, but it was normalized after 2 h (Figure 8), which goes along with the findings that the bacteria regained growth at later time points at these AA concentrations (Figure 1). Further studies showed that AA did not have any significant effect on the membrane ATPase activity (Supplementary Figure S8A), but increased the membrane fluidity in a dose-dependent manner (Supplementary Figure S8B).

### 3.6 Arachidonic acid causes an accumulation of 4,6-diamidino-2-phenylindole (DAPI) in live, but not fixed bacteria

**Figure 9:**
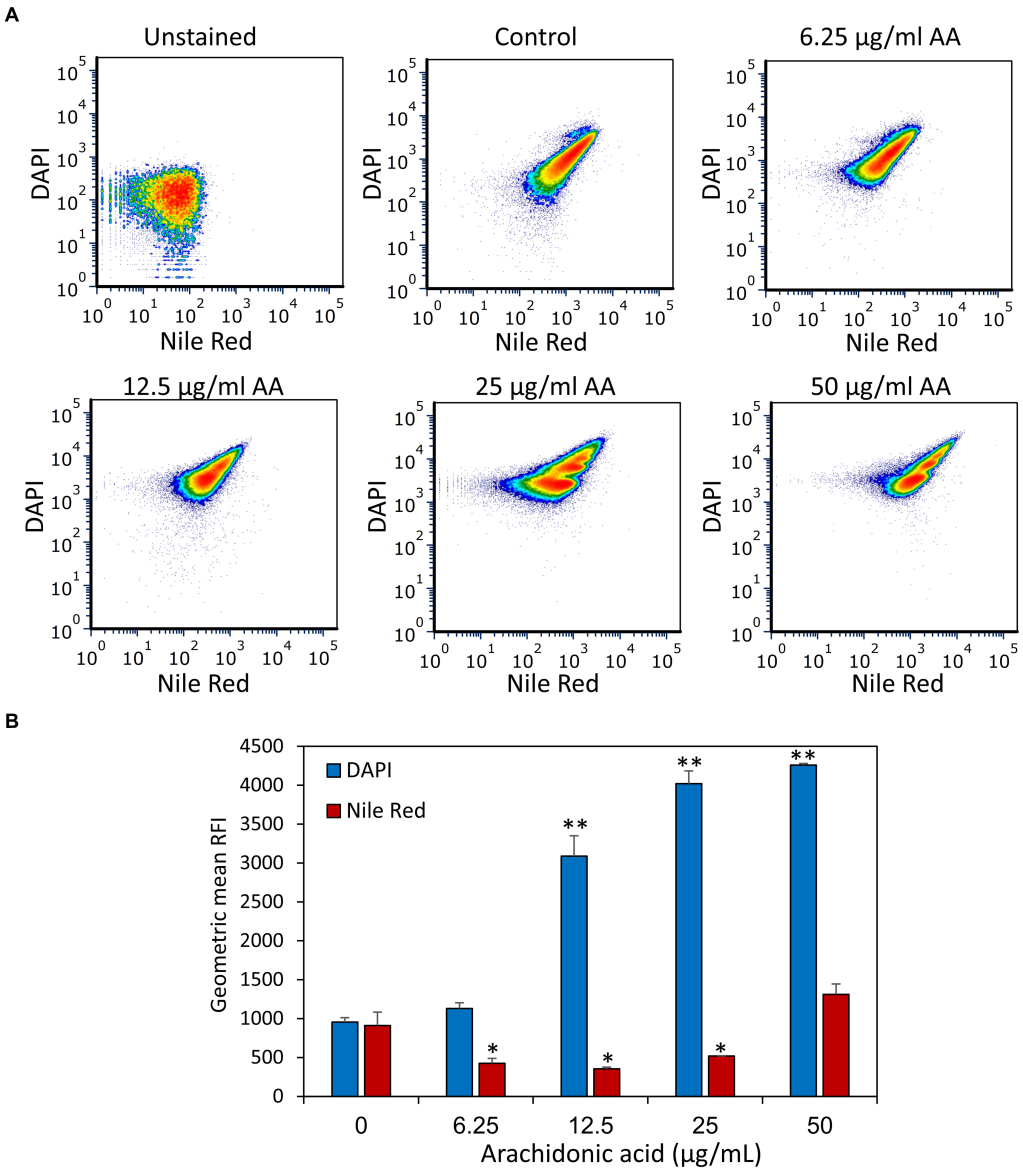
Treatment of *S. mutans* with arachidonic acid (AA) leads to an intracellular accumulation of DAPI. **(A)** Flow cytometry analysis of DAPI and Nile Red staining of live *S. mutans* that have been treated with various concentrations of AA for 2 h. **(B)** The geometric mean of relative fluorescence intensities (RFIs) of DAPI and Nile Red for the different treatment groups. *N* = 3. *p < 0.05 compared to control. **p < 0.001 compared to control.

**Figure 10:**
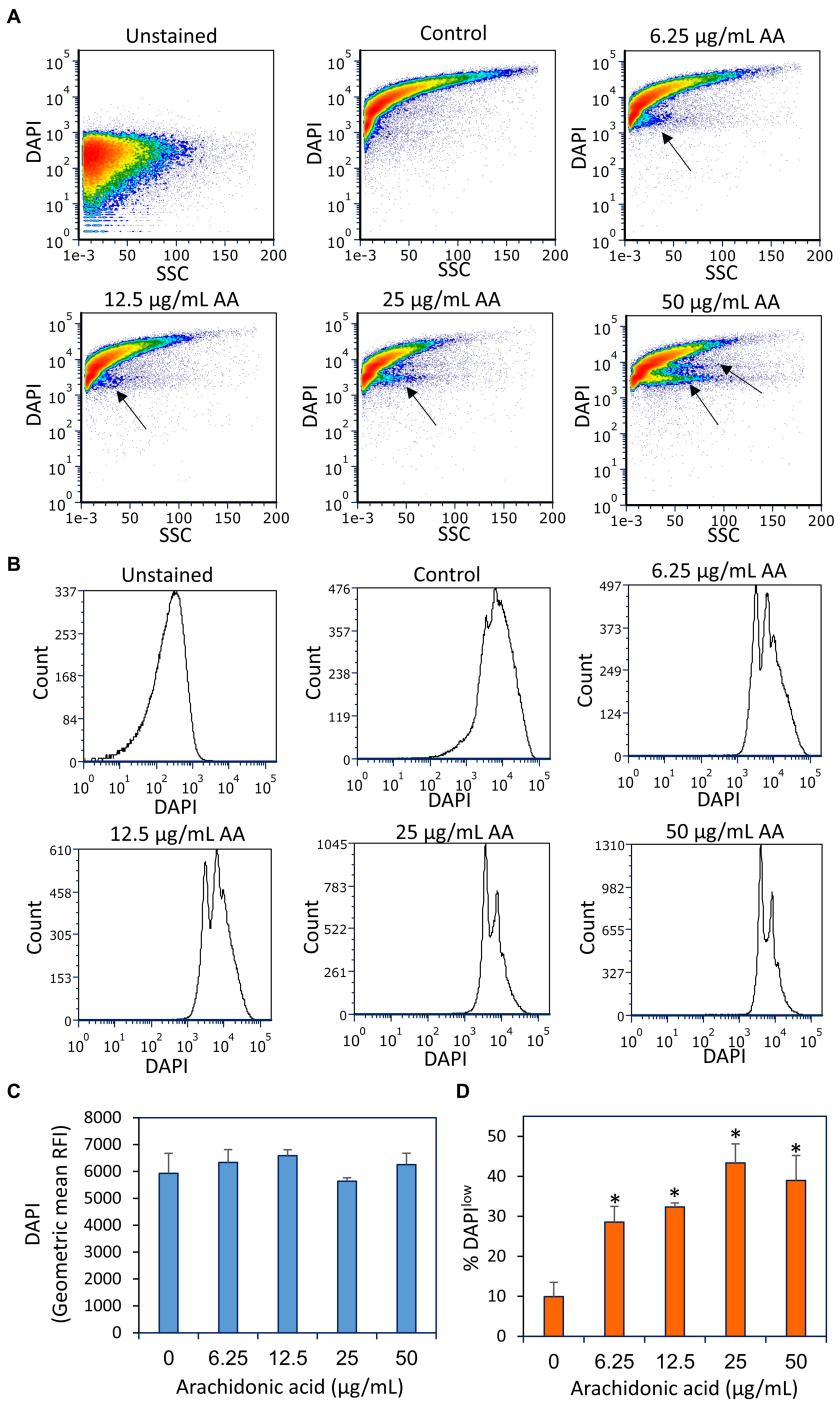
Fixation of the arachidonic acid (AA)-treated bacteria prior to DAPI staining did not lead to intracellular DAPI accumulation, but rather to the appearance of DAPI^low^ cell populations as indicated by black arrows. **(A)** Flow cytometry density plots of DAPI staining of fixed control and AA-treated bacteria. The bacteria were incubated with AA for 2 h and fixed with methanol, rehydrated and stained with DAPI. **(B)** Histograms of DAPI staining of the same samples as in **(A)**. **(C)** The geometric mean of relative fluorescence intensities (RFIs) of DAPI stain fixed bacteria. *N* = 3. **(D)** The percentage of DAPI^low^ cells of the samples in **(A)**. *N* = 3. *p < 0.05 compared to control. SSC, side scatter on flow cytometry.

DAPI is a neutral molecule that can freely enter both live and dead bacteria and fluoresces when binding to DNA. It has been used to measure the DNA content of cells including bacteria, but it can also be a substrate of efflux pumps (Kouidhi et al., 2011; Banerjee et al., 2021) and thus be a measure for efflux pump activities. *S. mutans* that have been treated with various concentrations of AA for 2 h were stained with both DAPI and the lipophilic dye Nile Red which emits red fluorescence when integrated into the membrane. Surprisingly, AA treatment resulted in a dose-dependent accumulation of DAPI, while Nile Red fluorescence was reduced by AA doses of 6.25–25 μg/ml AA, with a slight increase at 50 μg/ml AA (Figure 9 and Supplementary Figure S9). At the higher AA doses (25 and 50 μg/ml), there are two peaks of different DAPI intensities (Supplementary Figure S9), which might represent different amounts of DNA or differential accumulation of DAPI. To distinguish between these possibilities, the control group and AA-treated bacteria were fixed with methanol prior to DAPI staining. The fixation prevents bioactive efflux, while it preserves the DNA, such that the DNA intensity of fixed bacteria reflects the DNA content (Pozarowski and Darzynkiewicz, 2004). After fixation, AA did not affect the mean geometric fluorescence intensities (Figure 10), which is in contrast to DAPI staining of live bacteria (Figure 9). This finding suggests that AA may either prevent an efflux mechanism or increase an influx transport system that uses DAPI as a substrate. Nile Red has also been shown to serve as an efflux pump substrate (Bohnert et al., 2010). As we have observed here, the effect of AA on DAPI and Nile Red influx/efflux shows quite different patterns, suggesting an involvement of different membrane transport mechanisms that are differentially affected by AA.

Notably, DAPI staining of fixed AA-treated bacteria resulted in the appearance of two DAPI^low^ subpopulations, that reached up to 40–45% of the cells at the higher AA doses (25–50 μg/ml) (Figure 10). The DAPI^low^ population represents cells with lower DNA content, which might be due to a growth arrest before initiation of DNA replication. Notably, there was no general loss of DAPI staining, all being far above the threshold of unstained bacteria, indicating that the DNA is retained within the bacteria.

### 3.7 Differential accumulation of efflux pump substrates following arachidonic acid treatment of *Streptococcus mutans*

**Figure 11:**
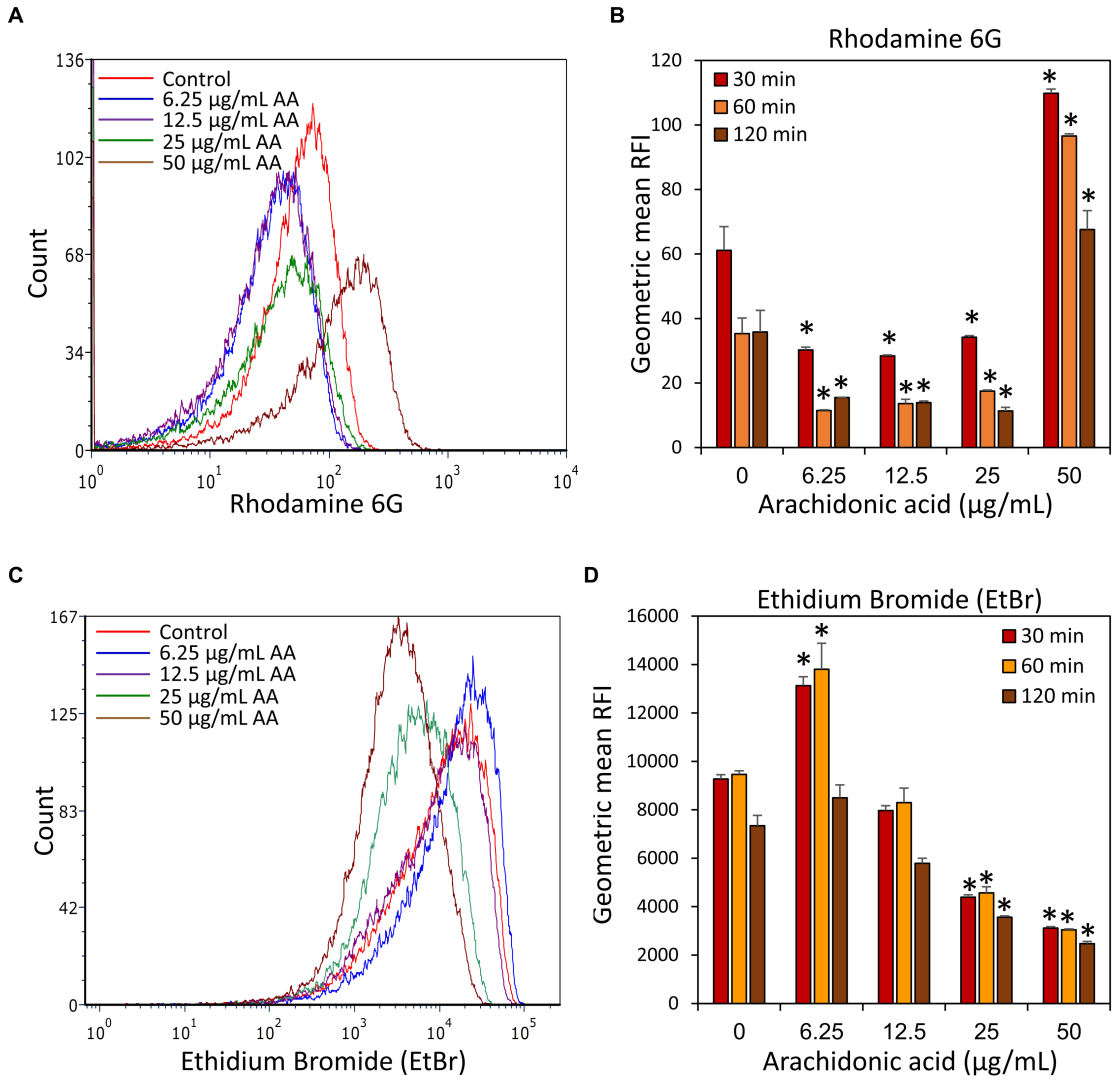
Accumulation and efflux of Rhodamine 6G and ethidium bromide (EtBr) of control *S. mutans* and bacteria treated with various concentrations of arachidonic acid (AA) for 2 h. **(A)** After a 2 h incubation with or without AA, the bacteria were loaded with Rhodamine 6G, washed, and the fluorescence intensity measured by flow cytometry after 30 min. **(B)** The geometric mean relative fluorescence intensity (RFI) of Rhodamine 6G-loaded samples 30, 60, and 120 min after dye removal. **(C)** After a 2 h incubation with or without AA, the bacteria were loaded with Ethidium bromide (EtBr), washed, and the fluorescence intensity measured by flow cytometry after 30 min. **(D)** The geometric mean of relative fluorescence intensity (RFI) of Ethidium bromide (EtBr)-loaded samples 30, 60, and 120 min after dye removal. *N* = 3. *p < 0.05 compared to control.

In light of the observation that DAPI accumulates in *S. mutans* following a 2 h treatment with AA, it was prompting to study whether AA also affects the uptake of other efflux pump substrates. We chose to use Rhodamine 6B and Ethidium bromide (EtBr), both being fluorescent dyes frequently used to study efflux pump activities (Paixão et al., 2009; Zeng et al., 2017; Whittle et al., 2019; Lu et al., 2020; Banerjee et al., 2021). Rhodamine 6G accumulation took a pattern similar to Nile Red with less accumulation in bacteria treated for 2 h with 6.25, 12.5, and 25 μg/ml AA, while an increased accumulation in bacteria treated with 50 μg/ml AA (Figures 11A,B). Studying the release of Rhodamine 6G after removing AA, the dye was extruded from all samples, although much slower in 50 μg/ml AA-treated bacteria (Figure 11B). Ethidium bromide presented a quite different pattern with an unexpectedly increased accumulation in the 6.25 μg/ml AA-treated bacteria, and a strong reduction of its uptake in 25 and 50 μg/ml AA-treated bacteria (Figures 11C,D). Likewise, the efflux was stronger in the 6.25 μg/ml AA-treated bacteria, while both processes were less active in the 25 and 50 μg/ml AA-treated bacteria (Figure 11D). In contrast to DAPI that stains only DNA, ethidium bromide stains both DNA and RNA (Kapuściński and Yanagi, 1979), such that the reduced ethidium bromide staining could also reflect reduced amount of RNA. Altogether, these data suggest that different efflux mechanisms are used for the different dyes, and these processes are differentially regulated by AA in a dose-dependent manner.

### 3.8 Arachidonic acid acts as an antioxidant in *Streptococcus mutans*

**Figure 12:**
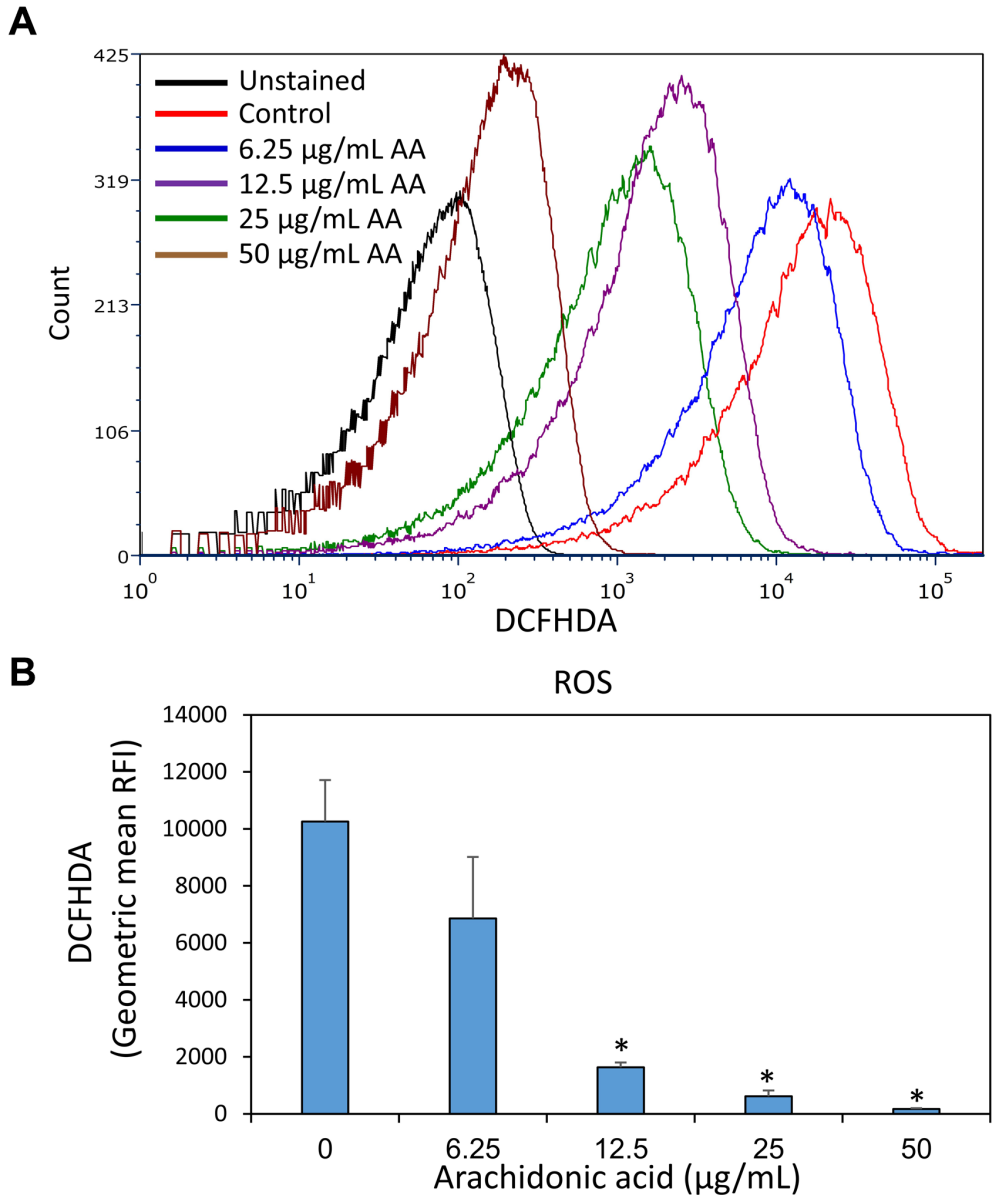
Arachidonic acid possesses anti-oxidative properties. *S. mutans* that have been incubated in the absence or presence of various concentrations of arachidonic acid (AA) for 2 h, were loaded with the redox probe 2′,7′-dichlorofluorescin diacetate (DCFHDA) and the fluorescence intensities were analyzed by flow cytometry. **(A)** The histograms of DCFHDA-loaded control and AA-treated bacteria. **(B)** The geometric mean of relative fluorescence intensities (RFIs) of the samples of **(A)**. *N* = 3. *p < 0.05 compared to control.

Since AA possesses a polyunsaturated alkyl chain, it was of interest to investigate whether it acts as an antioxidant in *S. mutans*. This is inquiring in view of the fact that several antioxidants have anti-bacterial activities (Sendamangalam et al., 2011; Stagos et al., 2012; Martelli and Giacomini, 2018; Schneider-Rayman et al., 2021). To this end, control and AA-treated bacteria after a 2 h incubation were loaded with the redox probe 2′,7′-dichlorofluorescin diacetate (DCFHDA) and the fluorescence intensities were analyzed by flow cytometry. Control bacteria were stained with DCFHDA, indicating that they produce reactive oxygen species (ROS) (Figures 12A,B). The fluorescence intensity of DCFHDA was reduced by AA in a dose-dependent manner (Figures 12A,B), indicating that it has antioxidant properties and reacts with the ROS produced by *S. mutans*.

**Figure 13:**
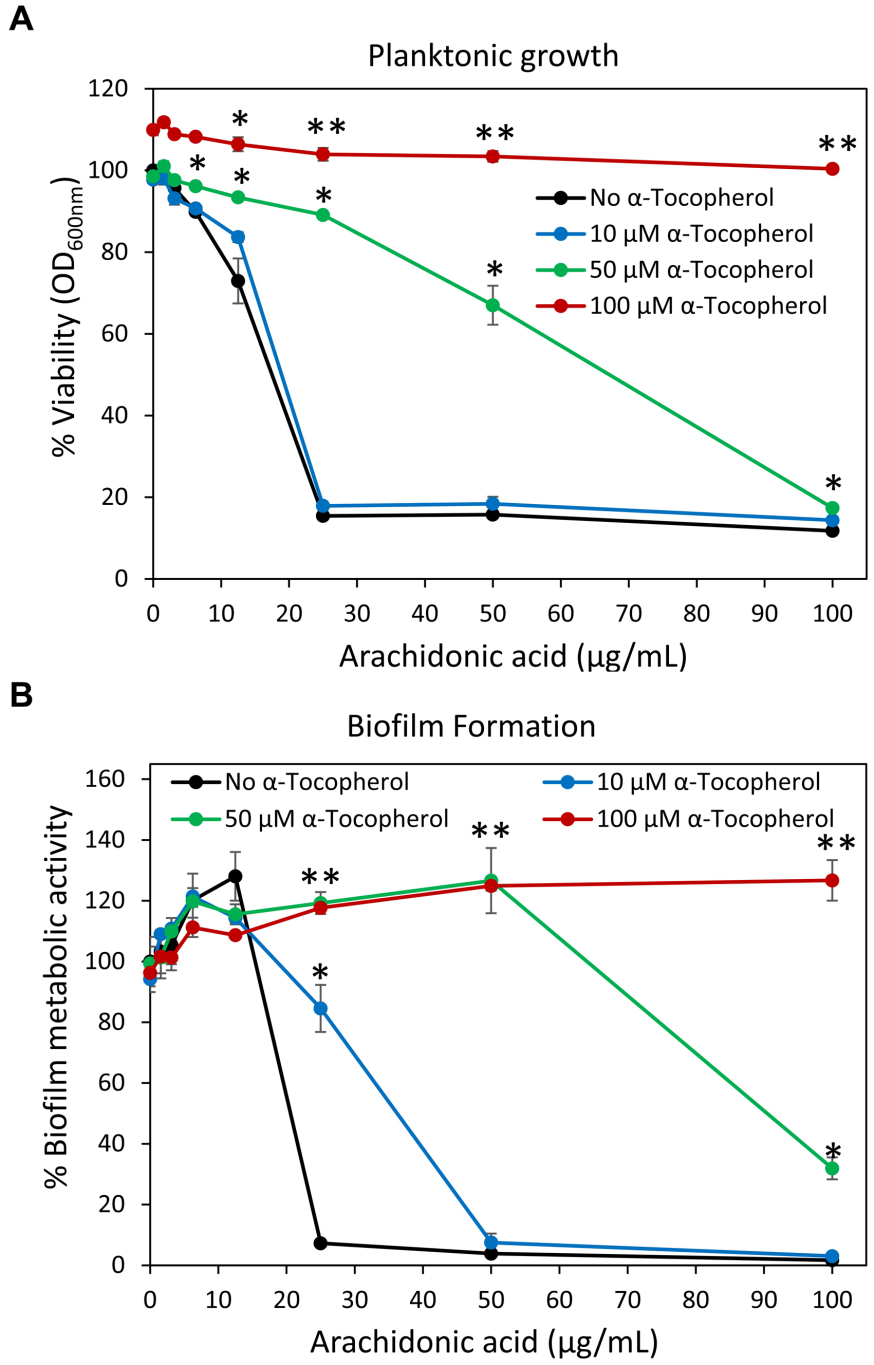
**(A,B)** α-Tocopherol antagonized the anti-bacterial **(A)** and anti-biofilm **(B)** activities of arachidonic acid against *S. mutans*. *S. mutans* was incubated with α-tocopherol in the absence or presence of various concentrations of arachidonic acid for 24 h. The planktonic growth was analyzed by OD at 600 nm. The metabolic activity of biofilms formed in the presence of various combinations of compounds was analyzed by MTT metabolic assay. *N* = 3. *p < 0.05 compared to AA-treated samples. **p < 0.001 compared to AA-treated samples.

A previous work of Beavers et al. (2019) suggested that AA kills *Staphylococcus aureus* by a mechanism involving lipid peroxidation. The killing could be prevented by α-tocopherol (Vitamin E) (Beavers et al., 2019) which acts as a lipid peroxyl radical scavanger (Liebler et al., 1986; Lebold and Traber, 2014). To study whether this also holds true for *S. mutans*, we incubated the bacteria with different concentrations (10–100 μM) of Vitamin E, the redox antioxidant agent ascorbic acid (Vitamin C) (Gęgotek and Skrzydlewska, 2022) or the reducing antioxidant agent N-acetyl-L-cysteine (Aldini et al., 2018) together with various concentrations of AA, and the resulting planktonic growth and biofilm formation were measured after 24 h. α-Tocopherol antagonized the anti-bacterial and anti-biofilm activities of AA at 50 and 100 μM (Figures 13A,B). Ten μM α-Tocopherol did not change the MIC value of AA (Figure 13A), but increased the MBIC value from 25 to 50 μg/mL AA (Figure 13B). Neither ascorbic acid nor N-acetyl-L-cysteine had any effect on the anti-bacterial and anti-biofilm activities of AA at the concentrations studied (Supplementary Figures S10, S11). This might be due to the water-soluble nature of ascorbic acid and N-acetyl-L-cysteine which do not penetrate the hydrophobic membrane regions reached by α-tocopherol and AA.

### 3.9 Gene expression affected by arachidonic acid in *Streptococcus mutans*

**Figure 14:**
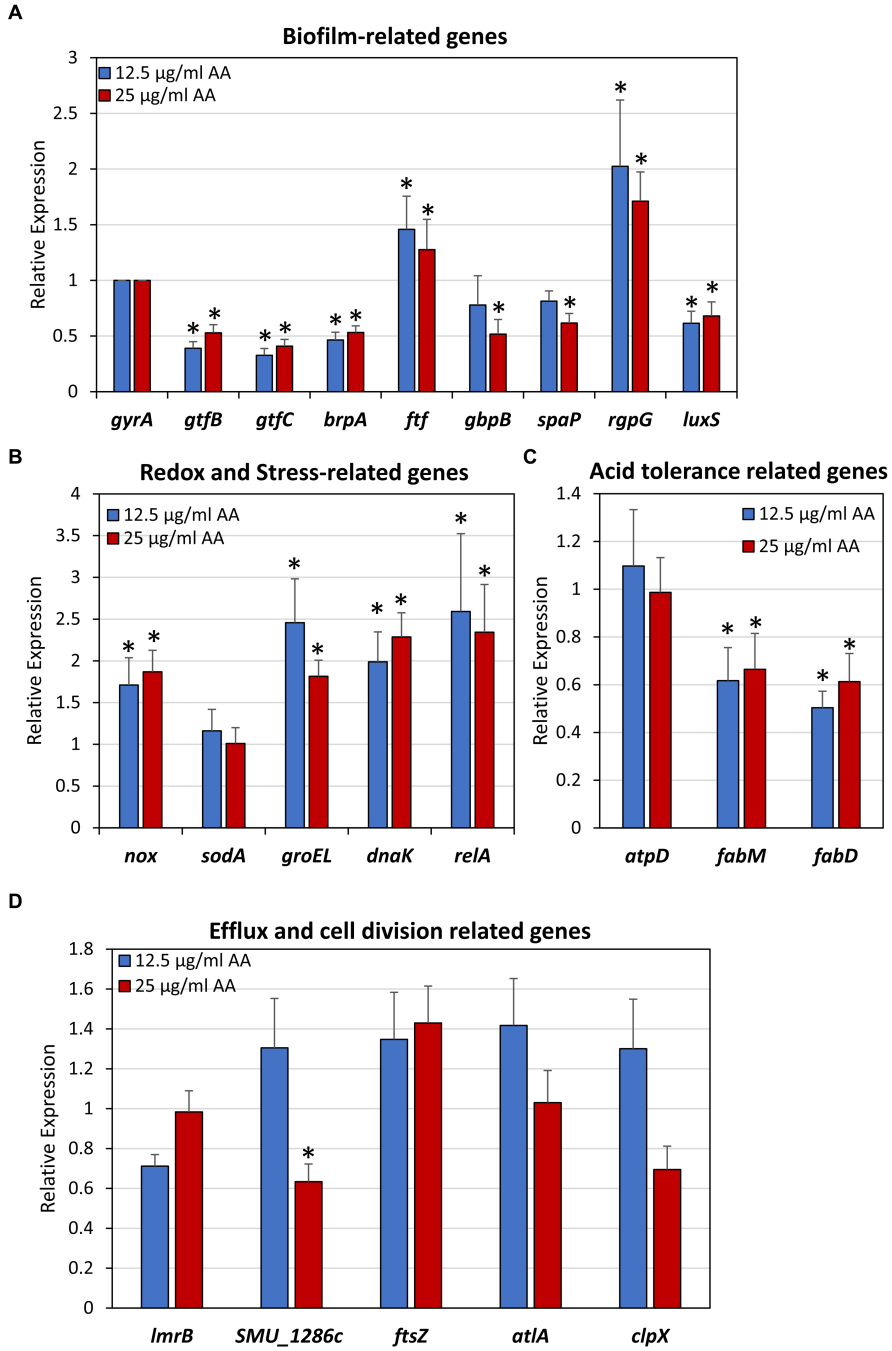
Effect of arachidonic acid on the expression of biofilm-related genes **(A)**, redox and stress-related genes **(B)**, acid tolerance-related genes **(C)** and efflux and cell division-related genes **(D)**. *S. mutans* was exposed to 12.5 and 25 μg/mL AA for 2 h, and then subjected to RNA isolation, cDNA conversion and real-time quantitative PCR. Each treatment was performed in triplicates, and the fold changes were calculated for each treated samples against each of 3 controls using the 2^–ΔΔCt^ method and *gyrA* as internal standard. The average of 9 calculations of each treatment group is shown together with the standard deviation. *p < 0.05 compared to control.

Besides the direct effects of AA on membrane properties, efflux pumps and oxidation described above, it could be that AA also has an effect on gene expression. We specifically looked at genes that are related to biofilm formation (Figure 14A), redox and stress-regulated genes (Figure 14B), acid tolerance (Figure 14C), efflux and cell division (Figure 14D) after a 2 h incubation with 12.5 or 25 μg/ml AA in the presence of 2% sucrose. The 2 h incubation period was chosen in order to catch the early changes in gene expression caused by AA. All gene expression analyses were done against DNA gyrase subunit A (*gyrA*) which served as a house-keeping gene (Nakano et al., 2007). Both AA concentrations resulted in a 50–60% reduction in the expression of glucosyltransferases *gtfB* and *gtfC*, and biofilm regulator protein A (*brpA*) involved in biofilm formation, while fructosyltransferase (*ftf*) and *rgpG* that catalyzes the first step of rhamnose-containing glucose polymers, were upregulated 1.5-2-fold (Figure 14A). A 40–50% reduction of glucan binding protein B (*gbpB*), the sucrose-independent adhesin *spaP,* and the autoinducer-2 synthase *luxS* was observed with the higher AA concentration of 25 μg/ml (Figure 14A). Both concentrations of AA increased the expression of NADH oxidase (*nox*) which reduces oxygen to water, the stress-response chaperones *groEL* and *dnaK*, and the (p)ppGpp synthase *relA*, but had no effect on superoxide dismutase (*sodA*) gene expression (Figure 14B). AA had no effect on *atpD* expression which is a subunit of ATP synthase important for acid adaptation, but reduced the expression of *fabM* and *fabD* involved in fatty acid homeostasis (Figure 14C). AA had no significant effect on the expression of the cell division related gene *ftsZ*, the autolysin *atlA*, and the ATPase *clpX* which regulates ClpP protease activity (Figure 14D). Twenty-five μg/ml AA caused a small reduction in the expression of the multidrug efflux protein *SMU_1286c*, but had no effect on the expression of the efflux transporter *lmrB* (Figure 14D).

### 3.10 Arachidonic acid was non-toxic to normal Vero epithelial cells and did not cause hemolysis

**Figure 15:**
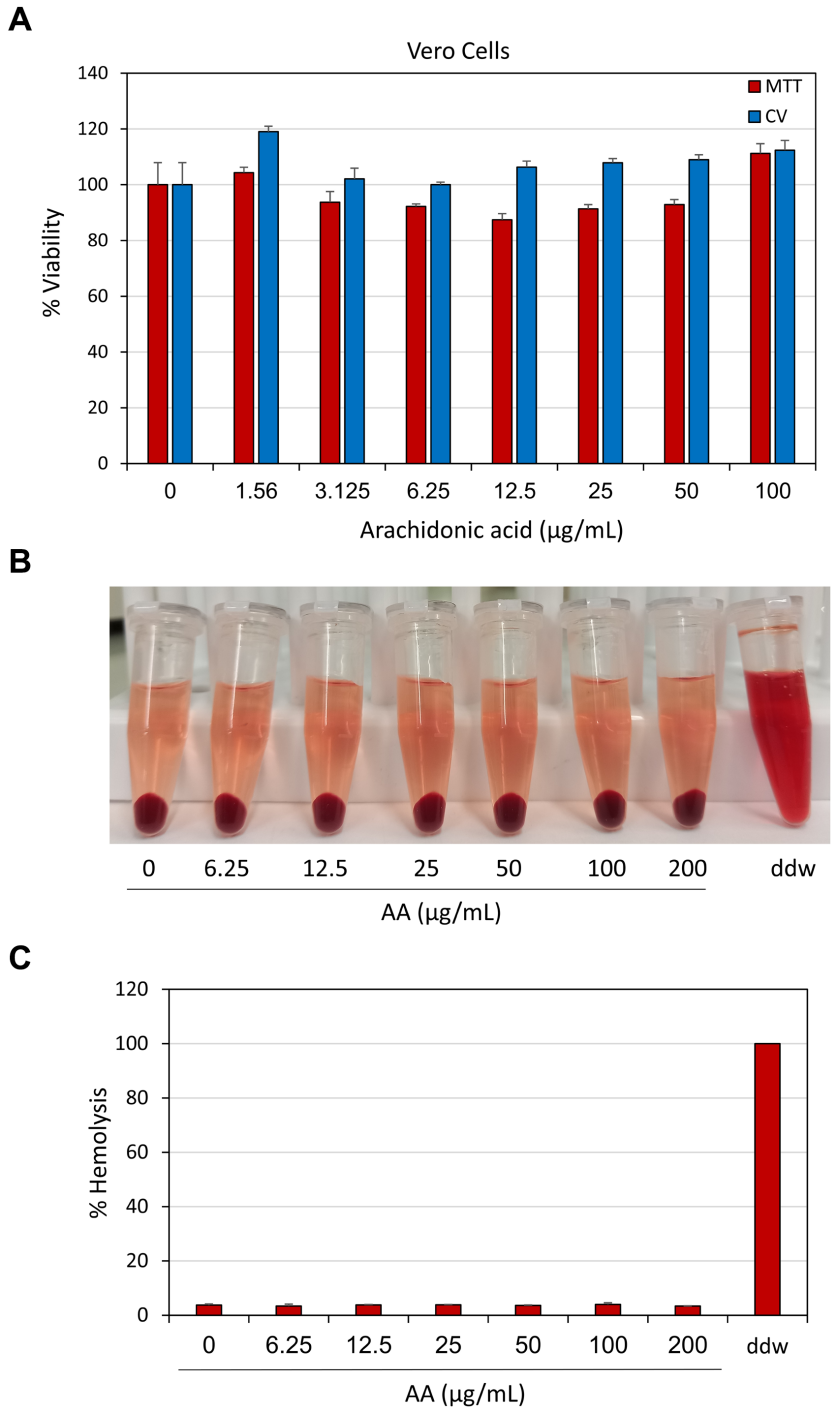
**(A)** Cytotoxicity assay of arachidonic acid (AA) on normal Vero epithelial cells. Monolayers of Vero epithelial cells were exposed to indicated concentrations of AA for 24 h and the cell mass was determined by crystal violet (CV) staining, and the metabolic activity determined by the MTT metabolic assay. **(B)** Hemolytic assay. Sheep erythrocytes were exposed to indicated concentrations of AA in PBS supplemented with 1% BSA for 1 h at 37°C. Double distilled water (ddw) was used as positive control for hemolysis. *N* = 3. **(C)** The percentage hemolysis of samples presented in **(B)** in comparison to ddw-induced hemolysis that was set to 100%.

It was important to study whether arachidonic acid is toxic to normal mammalian cells. For this purpose, we used normal Vero epithelial cells which is the gold standard for testing cytotoxicity (ISO 10993-5 (2009) recommendations; Standard, 2009), and exposed them to increasing concentrations of AA up to 100 μg/ml which is far above the MIC and MBIC against *S. mutans*. After a 24 h incubation, both the cell mass and metabolic activity were studied by crystal violet (CV) staining and MTT assay, respectively. Both assays showed that AA has no significant cytotoxic effect against Vero cells, even at the highest dose of 100 μg/ml (Figure 15A). Further studies with other cell lines showed that the lethal concentration of AA for CAL27 oral squamous carcinoma cells was 100 μg/ml, while it was 200 μg/ml for MCF-7 breast cancer cells and the HaCaT keratinocyte cell line (data not shown).

Next, we studied whether AA causes hemolysis. To this end, washed sheep erythrocytes were exposed to AA in PBS supplemented with 1% BSA for 1 h at 37°C, and the level of hemoglobin from exploded erythrocytes was measured in the supernatant. AA did not cause hemolysis under these conditions, even at a concentration of 200 μg/ml (Figures 15B,C). Thus, according to the above results, the use of AA against *S. mutans* can be considered safe.

## 4 Discussion

Arachidonic acid is a natural polyunsaturated fatty acid found in various food sources (e.g., eggs, fish, and poultry), and is integrated into the membrane phospholipids where it is released as AA or endocannabinoids such as anandamide and 2-AG upon various stimuli (Tallima and El Ridi, 2018). Released AA is metabolized to prostaglandins, thromboxanes and leukotrienes (Das, 2018; Tallima and El Ridi, 2018), but it is also secreted from activated macrophages where it is thought to enhance anti-bacterial activity (Das, 2018). Moreover, AA increases the phagocytic activity of macrophages and increases the intracellular killing of ingested bacteria (Anes et al., 2003; Adolph et al., 2012; Schumann, 2016; Eijkelkamp et al., 2018). Several bacterial components including lipopolysaccharides from Gram-negative bacteria, lipoteichoic acid (LTA) from Gram-positive bacteria and insoluble glucans from *S. mutans* can stimulate arachidonic acid mobilization in macrophages with concomitant increased production of prostaglandin E_2_ and thromboxane B2 (Abiko et al., 1983; Shibata et al., 1989; Card et al., 1994).

Arachidonic acid has been shown to have anti-bacterial effects against the Gram-positive bacteria *S. pneumoniae* (Eijkelkamp et al., 2018) and *S. aureus* (Beavers et al., 2019, 2022). In our initial screening for bacteria that respond to arachidonic acid, we observed that this fatty acid has anti-bacterial activity against the Gram-positive bacteria: *S. mutans*, *S. sobrinus*, methicillin-sensitive *S. aureus* (MSSA), methicillin-resistant *S. aureus* (MRSA) and one multidrug-resistant clinical *S. aureus* isolate (MDRSA), while no effect was observed on the Gram-negative *Pseudomonas aeruginosa* (Figure 1 and unpublished data). The anti-bacterial activities of AA together with its low cytotoxicity makes AA an attractive compound to be used to reduce bacterial burden of responding bacterial species and therefore may be a potential alternative to antibiotics (Desbois and Smith, 2010; Eijkelkamp et al., 2018; Beavers et al., 2019; Casillas-Vargas et al., 2021; Figures 1, 15). It is worth mentioning that AA has been administrated to children at a dose of 10 mg/kg for 15 days, resulting in around 50% cure rate of *Schistosoma mansoni*-infected children (Barakat et al., 2015). This and other studies showed that AA treatment was safe (Barakat et al., 2015; Tallima et al., 2020).

In the present study, we focused on the anti-bacterial and anti-biofilm activities of AA on the oral cariogenic *S. mutans*. We observed that the MIC and MBIC of AA are the same, with a value of 25 μg/ml when incubated in an atmosphere containing 5% CO_2_ (Figures 1A, 2A). This concentration is below the documented AA serum levels in humans (110–490 μg/ml) (Suwa et al., 2020). Interestingly, when incubated in an atmosphere without CO_2_ supplementation, the bacteria became much more sensitive to AA, with a MIC value reduced to 6.25–12.5 μg/ml (Figure 1B). The antagonistic effect of CO_2_ on the anti-bacterial effect of AA may be related to the ability of *S. mutans* to use CO_2_ as a carbon source. CO_2_ is used in the conversion of phosphoenolpyruvate to oxaloacetate catalyzed by phosphoenolpyruvate carboxykinase (Yamada and Carlsson, 1973; Franke and Deppenmeier, 2018). This process leads to generation of ATP (Yamada and Carlsson, 1973; Franke and Deppenmeier, 2018). It is likely that the conversion of phosphoenolpyruvate to oxalacetate in the presence of CO_2_ may competitively decrease the conversion of phosphoenolpyruvate to pyruvate and consequent lactate production. CO_2_ is also important for cytoplasmic buffering in *S. mutans* (Lemos et al., 2019).

The major energy source of *S. mutans* comes from glycolysis, as these bacteria do not possess oxidative phosphorylation due to incomplete electron transport chain system (Abranches et al., 2018; Lemos et al., 2019). *S. mutans* undergoes metabolic remodeling when the pH drops, resulting in increased glycolytic activity and increased F_1_F_0_-ATPase activity (Baker et al., 2015; Jijakli and Jensen, 2019; Lemos et al., 2019). We therefore wondered whether AA could affect membrane ATPase activity. However, we found that AA did not alter the ATPase activity of isolated membranes (Supplementary Figure S8A), nor did it affect the *atpD* (ATP synthase subunit beta) gene expression level (Figure 14). Instead, AA treatment induced membrane hyperpolarization in a dose-dependent manner (Figure 8), suggesting an interruption of the proton motive force. The latter is important for several vital bacterial processes (Benarroch and Asally, 2020), which might be an underlying cause for the AA-induced growth inhibition. Besides growth inhibition, AA also has a bactericidal effect on *S. mutans* (Figure 2). The number of colony forming units was reduced upon time in the presence of AA, which goes along with reduced ATP content (Figure 2). AA increases membrane permeability as observed by a dose-dependent increase in propidium iodide uptake, followed by membrane leakage as shown by a dose-dependent reduction in SYTO 9 fluorescence (Figure 4). HR-SEM images of AA-treated bacteria showed the presence of cell debris and remnants of burst bacteria (Figures 5, 6). This might be due to alterations in membrane properties caused by AA.

At low pH, *S. mutans* incorporates a higher fraction of monounsaturated fatty acids into the plasma membrane, which is thought to be important for the adaptation to acidic stress (Fozo et al., 2004; Fozo and Quivey, 2004). The production of monounsaturated fatty acids is mediated by FabM encoding a trans-2-*cis*-3-decanoyl-ACP isomerase (Fozo and Quivey, 2004). Gene expression analysis of AA-treated *S. mutans* showed only a slight, but significant, reduction in the expression of *fabM* as well as *fabD* encoding malonyl coenzyme A-acyl carrier protein transacylase involved in fatty acid synthesis (Figure 14). Thus, AA might affect fatty acid synthesis, which has also been suggested by Eijkelkamp et al. (2018) as a mechanism for the anti-bacterial action of AA against *S. pneumoniae*. It is likely that the inhibition of fatty acid production by AA is a negative feedback mechanism (Lambert et al., 2022). Since fatty acid synthesis is essential for bacterial survival (Balemans et al., 2010), its inhibition may affect bacterial viability.

In analogy to the incorporation of monounsaturated fatty acids, AA can be incorporated into the membrane of the bacteria (Eijkelkamp et al., 2018). This is reflected by a higher membrane fluidity observed in AA-treated bacteria (Supplementary Figure S8B and Eijkelkamp et al., 2018). Within the membrane, the AA moiety with four unsaturated carbon–carbon bonds can be oxidized by ROO· radicals resulting in lipid peroxidation (Beavers et al., 2019), inhibition of essential membrane protein activities (Beavers et al., 2019) and collapse of the cell membrane (Stagos et al., 2012). We observed that *S. mutans* constitutively produces reactive oxygen species (ROS) as detected by the redox probe 2′,7′-dichlorofluorescin diacetate (DCFHDA) (Figure 12). AA reduced the ROS levels in *S. mutans* in a dose-dependent manner, indicating that AA acts as a potent antioxidant (Figure 12). The reaction of AA with ROS causes a concomitant oxidation of AA, which in turn leads to lipid peroxidation as demonstrated by Beavers et al. (2019). This scenario is supported by the antagonistic effect of α-tocopherol on the anti-bacterial and anti-biofilm effect of AA on *S. mutans* (Figure 13). α-Tocopherol acts as a peroxyl radical chain-terminating agent (Liebler et al., 1986), and has also been shown by Beavers et al. (2019) to inhibit the AA-mediated toxicities against *S. pneumoniae*. Another possible anti-bacterial mechanism of AA could be an alteration of the redox balance that affects bacterial survival (Reniere, 2018). This possibility is based on findings implementing ROS species in both pro-survival signaling and anti-bacterial activities (Paiva and Bozza, 2014; Zhao and Drlica, 2014; Hong et al., 2019). This may also explain the anti-bacterial activity of various antioxidants (e.g., Sendamangalam et al., 2011; Stagos et al., 2012; Martelli and Giacomini, 2018; Schneider-Rayman et al., 2021).

Several membrane proteins are involved in nutrient uptake, transmembrane transport of enzymes such as Gtfs, and in the removal of toxic metabolites. *S. mutans* harbors 14 phosphoenolpyruvate-dependent sugar:phosphotransferase systems (PTSs) with specificities for various mono- and di-saccharides, some efflux pumps, and several ATP-binding cassette (ABC) transporters involved, among others, in internalization of oligosaccharides and export of competence-stimulating peptide (CSP) and mutacins (Hale et al., 2005; Suntharalingam and Cvitkovitch, 2005; Abranches et al., 2006; Webb and Hosie, 2006; Webb et al., 2008; Ahn et al., 2014; Nagayama et al., 2014; Liu et al., 2017; Lemos et al., 2019). We used different fluorescent efflux pump substrates to study the effect of AA on transmembrane transport, and observed dose-dependent and dye-dependent patterns of intracellular accumulation and export (Figures 9, 11). Notably, there was a dose-dependent increase in DAPI accumulation with increasing concentrations of AA (Figure 9), which resembled the DAPI accumulation observed with anandamide (Banerjee et al., 2021; Wolfson et al., 2023) and thymoquinone (Kouidhi et al., 2011). On the other hand, Nile Red and Rhodamine 6G showed a dose-dependent bell-shaped pattern with increased export at lower AA concentrations (6.25–25 μg/ml) while increased accumulation at 50 μg/ml AA (Figures 9, 11). Further studies showed increased accumulation of ethidium bromide at 6.25 μg/ml AA, with a gradual decrease upon increasing concentrations of AA (Figure 11). The opposite effects of dye uptake and dye release may be due to AA-induced alterations in the activity of different transport systems. Since these transport systems are also involved in nutrient uptake and excretion of toxic compounds, the interruption of which may lead to an imbalance of intracellular nutrients and toxic compounds which may contribute to the anti-bacterial effects of AA.

A major aim in caries prevention is to inhibit biofilm formation of cariogenic bacteria such as *S. mutans*. Although the AA concentration required for preventing biofilm formation was the same as that of the anti-bacterial effect against planktonic growing bacteria (Figure 2 versus Figure 1), gene expression studies suggest that AA also may have a direct anti-biofilm effect (Figure 14). Several of the biofilm-associated genes were down-regulated after a 2 h incubation with AA, including the glucosyltransferases *gtfB* and *gtfC* involved in exopolysaccharide production (Koo et al., 2010), *brpA* regulating biofilm formation and playing a role in acid and oxidative stress tolerance (Bitoun et al., 2012), *luxS* involved in autoinducer-2 production (Wright and Ramachandra, 2022), *gbpB* which binds to glucans (Mattos-Graner et al., 2006), and *spaP* which acts as an adhesin (Järvå et al., 2020; Figure 14). In contrast, *rgpG* that catalyzes the first step of rhamnose-containing glucose polymers (Yamashita et al., 1999) was upregulated (Figure 14). The rhamnose-glucose polysaccharides have been shown to protect *S. mutans* from various stress stimuli (Kovacs et al., 2019), such that the AA-mediated upregulation of *rgpG* might be a feedback mechanism to the cellular stress caused by AA. The latter is manifested by the upregulation of the two stress-response chaperones *groEL* and *dnaK* (Figure 14). The reduction of *gtfB* and *gtfC* genes which encode for the major enzymes involved in EPS production (Koo et al., 2010), may contribute to the reduced biofilm formation caused by AA. This assumption is based on findings that have shown a link between *gtf* gene expression and biofilm formation in *S. mutans* (He et al., 2019; Schneider-Rayman et al., 2021; Chi et al., 2022; Zheng et al., 2023). However, we cannot exclude the possibility that other mechanisms are also involved in the anti-biofilm activity of AA. HR-SEM images of biofilms showed the presence of EPS in samples exposed to 6.25 and 12.5 μg/ml AA, while no EPS could be discerned around the bacteria that had managed to adhere to plastic in the presence of 25 and 50 μg/ml AA (Figure 6). These data suggest that AA may impair EPS production. The altered membrane transport caused by AA (Figures 9, 11) may also contribute to the reduced EPS production since the latter depends on the secretion of Gtfs (Zhang et al., 2021; Zheng et al., 2023). Further studies are required to fully understand the molecular mechanisms involved in the anti-biofilm activity of AA.

In conclusion, we have shown here that arachidonic acid has both anti-bacterial and anti-biofilm activities against the cariogenic *S. mutans*. The anti-bacterial activity of AA is a combined effect of bacterial growth arrest and bacterial cell death. Treatment of *S. mutans* with AA leads to several changes in bacterial membrane properties including membrane hyperpolarization, increased membrane fluidity, changes in membrane transport, and membrane perforation with consequent cytoplasmic leakage. One of the action mechanisms is mediated by its anti-oxidant property, which leads to lipid oxidation. This in turn impairs the activity of membrane proteins that are, among others, involved in membrane transport, membrane integrity and the maintenance of membrane potential. Interference with these processes ultimately results in disruption of the energy supply and to the loss of bacterial viability. The anti-biofilm activity of AA is caused by a combination of its anti-bacterial activity, reduced expression of biofilm-associated genes, altered membrane transport, and reduced EPS production. The observation that AA is not toxic to normal Vero epithelial cells and does not cause hemolysis, even at concentrations well above the MIC and MBIC for *S. mutans*, is important because it does not limit therapeutic doses. This is actually not surprising given the many natural sources of AA. Overall, arachidonic acid can be considered a possible safe drug with potential benefits for dental caries prevention and the promotion of global oral health, based on its selective toxicity against cariogenic *S. mutans*. The abundant natural sources of AA further support the feasibility of its application in the clinical setting. The limitations of this study are that we only investigated the mechanisms of action against one clinical strain of *S. mutans*, and that it is an *in vitro* study. Further research should focus on the *in vivo* implications of using AA as an anti-bacterial agent to reduce dental caries.

## Data availability statement

The original contributions presented in the study are included in the article/Supplementary material, further inquiries can be directed to the corresponding author.

## Ethics statement

Ethical approval was not required for the studies on animals in accordance with the local legislation and institutional requirements because only commercially available established cell lines were used.

## Author contributions

MC: Data curation, Formal analysis, Investigation, Methodology, Software, Validation, Visualization, Writing – review & editing. JH: Data curation, Formal analysis, Investigation, Methodology, Software, Validation, Visualization, Writing – review & editing. RS: Conceptualization, Data curation, Formal analysis, Investigation, Methodology, Software, Supervision, Validation, Visualization, Writing – original draft, Writing – review & editing. DS: Conceptualization, Funding acquisition, Project administration, Resources, Supervision, Writing – review & editing.

## References

[ref1] Abiko Y. Shibata Y. Fukushima K. Murai S. Takiguchi H. (1983). The stimulation of macrophage prostaglandin E2 and thromboxane B2 secretion by *Streptococcus mutans* insoluble glucans. FEBS Lett. 154, 297–300. doi: 10.1016/0014-5793(83)80170-7, PMID: 6403385

[ref2] Abou Neel E. A. Aljabo A. Strange A. Ibrahim S. Coathup M. Young A. M.. (2016). Demineralization-remineralization dynamics in teeth and bone. Int. J. Nanomedicine 11, 4743–4763. doi: 10.2147/IJN.S107624, PMID: 27695330 PMC5034904

[ref3] Abranches J. Candella M. M. Wen Z. T. Baker H. V. Burne R. A. (2006). Different roles of EIIABMan and EIIGlc in regulation of energy metabolism, biofilm development, and competence in *Streptococcus mutans*. J. Bacteriol. 188, 3748–3756. doi: 10.1128/JB.00169-06, PMID: 16707667 PMC1482907

[ref4] Abranches J. Zeng L. Kajfasz J. K. Palmer S. R. Chakraborty B. Wen Z. T.. (2018). Biology of Oral streptococci. Microbiol. Spectr. 6:5. doi: 10.1128/microbiolspec.GPP3-0042-2018, PMID: 30338752 PMC6287261

[ref5] Adolph S. Fuhrmann H. Schumann J. (2012). Unsaturated fatty acids promote the phagocytosis of *P. Aeruginosa* and *R. equi* by RAW264.7 macrophages. Curr. Microbiol. 65, 649–655. doi: 10.1007/s00284-012-0207-3, PMID: 22903555

[ref6] Ahn S. J. Ahn S. J. Wen Z. T. Brady L. J. Burne R. A. (2008). Characteristics of biofilm formation by *Streptococcus mutans* in the presence of saliva. Infect. Immun. 76, 4259–4268. doi: 10.1128/IAI.00422-08, PMID: 18625741 PMC2519434

[ref7] Ahn S. J. Kaspar J. Kim J. N. Seaton K. Burne R. A. (2014). Discovery of novel peptides regulating competence development in *Streptococcus mutans*. J. Bacteriol. 196, 3735–3745. doi: 10.1128/JB.01942-14, PMID: 25135217 PMC4248802

[ref8] Aldini G. Altomare A. Baron G. Vistoli G. Carini M. Borsani L.. (2018). N-acetylcysteine as an antioxidant and disulphide breaking agent: the reasons why. Free Radic. Res. 52, 751–762. doi: 10.1080/10715762.2018.1468564, PMID: 29742938

[ref9] Anes E. Kühnel M. P. Bos E. Moniz-Pereira J. Habermann A. Griffiths G. (2003). Selected lipids activate phagosome actin assembly and maturation resulting in killing of pathogenic mycobacteria. Nat. Cell Biol. 5, 793–802. doi: 10.1038/ncb1036, PMID: 12942085

[ref10] Aragão M. G. B. Aires C. P. Corona S. A. M. (2022). Effects of the green tea catechin epigallocatechin-3-gallate on *Streptococcus mutans* planktonic cultures and biofilms: systematic literature review of *in vitro* studies. Biofouling 38, 687–695. doi: 10.1080/08927014.2022.2116320 36017657

[ref11] Avraham M. Steinberg D. Barak T. Shalish M. Feldman M. Sionov R. V. (2023). Improved anti-biofilm effect against the oral cariogenic *Streptococcus mutans* by combined Triclosan/CBD treatment. Biomedicines 11:521. doi: 10.3390/biomedicines11020521, PMID: 36831057 PMC9953046

[ref12] Baker J. L. Abranches J. Faustoferri R. C. Hubbard C. J. Lemos J. A. Courtney M. A.. (2015). Transcriptional profile of glucose-shocked and acid-adapted strains of *Streptococcus mutans*. Mol Oral Microbiol 30, 496–517. doi: 10.1111/omi.12110, PMID: 26042838 PMC4659386

[ref13] Balemans W. Lounis N. Gilissen R. Guillemont J. Simmen K. Andries K.. (2010). Essentiality of FASII pathway for *Staphylococcus aureus*. Nature 463:E3. doi: 10.1038/nature08667 20090698

[ref14] Banerjee S. Sionov R. V. Feldman M. Smoum R. Mechoulam R. Steinberg D. (2021). Anandamide alters the membrane properties, halts the cell division and prevents drug efflux in multidrug resistant *Staphylococcus aureus*. Sci. Rep. 11:8690. doi: 10.1038/s41598-021-88099-6, PMID: 33888802 PMC8062478

[ref15] Barakat R. Abou El-Ela N. E. Sharaf S. El Sagheer O. Selim S. Tallima H.. (2015). Efficacy and safety of arachidonic acid for treatment of school-age children in *Schistosoma mansoni* high-endemicity regions. Am. J. Trop. Med. Hyg. 92, 797–804. doi: 10.4269/ajtmh.14-0675, PMID: 25624403 PMC4385776

[ref16] Beavers W. N. Monteith A. J. Amarnath V. Mernaugh R. L. Roberts L. J. 2nd Chazin W. J.. (2019). Arachidonic acid kills *Staphylococcus aureus* through a lipid peroxidation mechanism. MBio 10, e01333–19. doi: 10.1128/mBio.01333-19, PMID: 31575763 PMC6775451

[ref17] Beavers W. N. Munneke M. J. Stackhouse A. R. Freiberg J. A. Skaar E. P. (2022). Host polyunsaturated fatty acids potentiate aminoglycoside killing of *Staphylococcus aureus*. Microbiol Spectr. 10:e0276721. doi: 10.1128/spectrum.02767-21, PMID: 35377191 PMC9045252

[ref18] Benarroch J. M. Asally M. (2020). The Microbiologist's guide to membrane potential dynamics. Trends Microbiol. 28, 304–314. doi: 10.1016/j.tim.2019.12.008, PMID: 31952908

[ref19] Bitoun J. P. Liao S. Yao X. Ahn S. J. Isoda R. Nguyen A. H.. (2012). BrpA is involved in regulation of cell envelope stress responses in *Streptococcus mutans*. Appl. Environ. Microbiol. 78, 2914–2922. doi: 10.1128/AEM.07823-11, PMID: 22327589 PMC3318800

[ref20] Bohnert J. A. Karamian B. Nikaido H. (2010). Optimized Nile red efflux assay of AcrAB-TolC multidrug efflux system shows competition between substrates. Antimicrob. Agents Chemother. 54, 3770–3775. doi: 10.1128/AAC.00620-10, PMID: 20606071 PMC2934970

[ref21] Button D. K. Robertson B. R. (2001). Determination of DNA content of aquatic bacteria by flow cytometry. Appl. Environ. Microbiol. 67, 1636–1645. doi: 10.1128/AEM.67.4.1636-1645.2001, PMID: 11282616 PMC92780

[ref22] Card G. L. Jasuja R. R. Gustafson G. L. (1994). Activation of arachidonic acid metabolism in mouse macrophages by bacterial amphiphiles. J. Leukoc. Biol. 56, 723–728. doi: 10.1002/jlb.56.6.723 7996048

[ref23] Casillas-Vargas G. Ocasio-Malavé C. Medina S. Morales-Guzmán C. Del Valle R. G. Carballeira N. M.. (2021). Antibacterial fatty acids: an update of possible mechanisms of action and implications in the development of the next-generation of antibacterial agents. Prog. Lipid Res. 82:101093. doi: 10.1016/j.plipres.2021.101093, PMID: 33577909 PMC8137538

[ref24] Chi Y. Wang Y. Ji M. Li Y. Zhu H. Yan Y.. (2022). Natural products from traditional medicine as promising agents targeting at different stages of oral biofilm development. Front. Microbiol. 13:955459. doi: 10.3389/fmicb.2022.955459, PMID: 36033896 PMC9411938

[ref25] Dann A. B. Hontela A. (2011). Triclosan: environmental exposure, toxicity and mechanisms of action. J. Appl. Toxicol. 31, 285–311. doi: 10.1002/jat.1660, PMID: 21462230

[ref26] Das U. N. (2018). Arachidonic acid and other unsaturated fatty acids and some of their metabolites function as endogenous antimicrobial molecules: a review. J. Adv. Res. 11, 57–66. doi: 10.1016/j.jare.2018.01.001, PMID: 30034876 PMC6052656

[ref27] Deo P. N. Deshmukh R. (2019). Oral microbiome: unveiling the fundamentals. J Oral Maxillofac Pathol. 23, 122–128. doi: 10.4103/jomfp.JOMFP_304_18, PMID: 31110428 PMC6503789

[ref28] Desbois A. P. Smith V. J. (2010). Antibacterial free fatty acids: activities, mechanisms of action and biotechnological potential. Appl. Microbiol. Biotechnol. 85, 1629–1642. doi: 10.1007/s00253-009-2355-3, PMID: 19956944

[ref29] Dewhirst F. E. Chen T. Izard J. Paster B. J. Tanner A. C. Yu W. H.. (2010). The human oral microbiome. J. Bacteriol. 192, 5002–5017. doi: 10.1128/JB.00542-10, PMID: 20656903 PMC2944498

[ref30] Ehteshami A. Shirban F. Gharibpour F. Bagherniya M. Sathyapalan T. Sahebkar A. (2021). Does curcumin have an anticaries effect? A systematic review of *in vitro* studies. Adv. Exp. Med. Biol. 1291, 213–227. doi: 10.1007/978-3-030-56153-6_12, PMID: 34331692

[ref31] Eijkelkamp B. A. Begg S. L. Pederick V. G. Trapetti C. Gregory M. K. Whittall J. J.. (2018). Arachidonic acid stress impacts pneumococcal fatty acid homeostasis. Front. Microbiol. 9:813. doi: 10.3389/fmicb.2018.00813, PMID: 29867785 PMC5958418

[ref32] Ells R. Kock J. L. Van Wyk P. W. Botes P. J. Pohl C. H. (2009). Arachidonic acid increases antifungal susceptibility of *Candida albicans* and *Candida dubliniensis*. J. Antimicrob. Chemother. 63, 124–128. doi: 10.1093/jac/dkn446, PMID: 18971215

[ref33] Fernandes M. Lourenço T. Lopes A. Spínola Santos A. Pereira Santos M. C. Pereira B. M. (2019). Chlorhexidine: a hidden life-threatening allergen. Asia Pac. Allergy 9:e29. doi: 10.5415/apallergy.2019.9.e29, PMID: 31720240 PMC6826114

[ref34] Forssten S. D. Björklund M. Ouwehand A. C. (2010). *Streptococcus mutans*, caries and simulation models. Nutrients 2, 290–298. doi: 10.3390/nu2030290, PMID: 22254021 PMC3257652

[ref35] Fozo E. M. Kajfasz J. K. Quivey R. G. Jr. (2004). Low pH-induced membrane fatty acid alterations in oral bacteria. FEMS Microbiol. Lett. 238, 291–295. doi: 10.1111/j.1574-6968.2004.tb09769.x, PMID: 15358413

[ref36] Fozo E. M. Quivey R. G. Jr. (2004). The *fabM* gene product of *Streptococcus mutans* is responsible for the synthesis of monounsaturated fatty acids and is necessary for survival at low pH. J. Bacteriol. 186, 4152–4158. doi: 10.1128/JB.186.13.4152-4158.2004, PMID: 15205416 PMC421590

[ref37] Franke T. Deppenmeier U. (2018). Physiology and central carbon metabolism of the gut bacterium *Prevotella copri*. Mol. Microbiol. 109, 528–540. doi: 10.1111/mmi.14058 29995973

[ref38] Gęgotek A. Skrzydlewska E. (2022). Antioxidative and anti-inflammatory activity of ascorbic acid. Antioxidants 11:1993. doi: 10.3390/antiox11101993 36290716 PMC9598715

[ref39] Giacaman R. A. Maturana C. A. Molina J. Volgenant C. M. C. Fernández C. E. (2023). Effect of casein phosphopeptide-amorphous calcium phosphate added to milk, chewing gum, and candy on dental caries: a systematic review. Caries Res. 57, 106–118. doi: 10.1159/000530638, PMID: 37054690

[ref40] Gil-de-Gómez L. Monge P. Rodríguez J. P. Astudillo A. M. Balboa M. A. Balsinde J. (2020). Phospholipid arachidonic acid remodeling during phagocytosis in mouse peritoneal macrophages. Biomedicines 8:274. doi: 10.3390/biomedicines8080274, PMID: 32764331 PMC7459916

[ref41] Guo L. McLean J. S. Lux R. He X. Shi W. (2015). The well-coordinated linkage between acidogenicity and aciduricity via insoluble glucans on the surface of *Streptococcus mutans*. Sci. Rep. 5:18015. doi: 10.1038/srep18015, PMID: 26657939 PMC4675080

[ref42] Gurunathan D. Somasundaram S. Kumar S. (2012). Casein phosphopeptide-amorphous calcium phosphate: a remineralizing agent of enamel. Aust. Dent. J. 57, 404–408. doi: 10.1111/adj.12006 23186562

[ref43] Hale J. D. Heng N. C. Jack R. W. Tagg J. R. (2005). Identification of *nlmTE*, the locus encoding the ABC transport system required for export of nonlantibiotic mutacins in *Streptococcus mutans*. J. Bacteriol. 187, 5036–5039. doi: 10.1128/JB.187.14.5036-5039.2005, PMID: 15995224 PMC1169533

[ref44] He Z. Huang Z. Jiang W. Zhou W. (2019). Antimicrobial activity of cinnamaldehyde on *Streptococcus mutans* biofilms. Front. Microbiol. 10:2241. doi: 10.3389/fmicb.2019.02241, PMID: 31608045 PMC6773874

[ref45] Hong Y. Zeng J. Wang X. Drlica K. Zhao X. (2019). Post-stress bacterial cell death mediated by reactive oxygen species. Proc. Natl. Acad. Sci. USA 116, 10064–10071. doi: 10.1073/pnas.1901730116, PMID: 30948634 PMC6525477

[ref46] Huang C. B. Alimova Y. Myers T. M. Ebersole J. L. (2011). Short- and medium-chain fatty acids exhibit antimicrobial activity for oral microorganisms. Arch. Oral Biol. 56, 650–654. doi: 10.1016/j.archoralbio.2011.01.011, PMID: 21333271 PMC3119748

[ref47] Huang S. Wu M. Li Y. Du J. Chen S. Jiang S.. (2022). The *dlt* operon contributes to the resistance to chlorhexidine in *Streptococcus mutans*. Int. J. Antimicrob. Agents 59:106540. doi: 10.1016/j.ijantimicag.2022.106540, PMID: 35092806

[ref48] Järvå M. A. Hirt H. Dunny G. M. Berntsson R. P. (2020). Polymer adhesin domains in gram-positive cell surface proteins. Front. Microbiol. 11:599899. doi: 10.3389/fmicb.2020.599899, PMID: 33324381 PMC7726212

[ref49] Jijakli K. Jensen P. A. (2019). Metabolic modeling of *Streptococcus mutans* reveals complex nutrient requirements of an oral pathogen. mSystems 4, e00529–e00519. doi: 10.1128/mSystems.00529-19 31662430 PMC6819733

[ref50] Johansson I. Witkowska E. Kaveh B. Lif Holgerson P. Tanner A. C. (2016). The microbiome in populations with a low and high prevalence of caries. J. Dent. Res. 95, 80–86. doi: 10.1177/0022034515609554, PMID: 26442950 PMC4700664

[ref51] Kapuściński J. Yanagi K. (1979). Selective staining by 4′, 6-diamidine-2-phenylindole of nanogram quantities of DNA in the presence of RNA on gels. Nucleic Acids Res. 6, 3535–3542. doi: 10.1093/nar/6.11.3535, PMID: 493114 PMC327954

[ref52] Kaseda K. Yokota H. Ishii Y. Yanagida T. Inoue T. Fukui K.. (2000). Single-molecule imaging of interaction between dextran and glucosyltransferase from *Streptococcus sobrinus*. J. Bacteriol. 182, 1162–1166. doi: 10.1128/JB.182.4.1162-1166.2000, PMID: 10648546 PMC94396

[ref53] Koo H. Xiao J. Klein M. I. Jeon J. G. (2010). Exopolysaccharides produced by *Streptococcus mutans* glucosyltransferases modulate the establishment of microcolonies within multispecies biofilms. J. Bacteriol. 192, 3024–3032. doi: 10.1128/JB.01649-09, PMID: 20233920 PMC2901689

[ref54] Kouidhi B. Zmantar T. Jrah H. Souiden Y. Chaieb K. Mahdouani K.. (2011). Antibacterial and resistance-modifying activities of thymoquinone against oral pathogens. Ann. Clin. Microbiol. Antimicrob. 10:29. doi: 10.1186/1476-0711-10-29, PMID: 21707998 PMC3146813

[ref55] Kovacs C. J. Faustoferri R. C. Bischer A. P. Quivey R. G. Jr. (2019). *Streptococcus mutans* requires mature rhamnose-glucose polysaccharides for proper pathophysiology, morphogenesis and cellular division. Mol. Microbiol. 112, 944–959. doi: 10.1111/mmi.14330, PMID: 31210392 PMC6736739

[ref56] Krzyściak W. Jurczak A. Kościelniak D. Bystrowska B. Skalniak A. (2014). The virulence of *Streptococcus mutans* and the ability to form biofilms. Eur. J. Clin. Microbiol. Infect. Dis. 33, 499–515. doi: 10.1007/s10096-013-1993-7, PMID: 24154653 PMC3953549

[ref57] Kumaratilake L. M. Robinson B. S. Ferrante A. Poulos A. (1992). Antimalarial properties of n-3 and n-6 polyunsaturated fatty acids: *in vitro* effects on *plasmodium falciparum* and *in vivo* effects on *P. berghei*. J. Clin. Invest. 89, 961–967. doi: 10.1172/JCI115678, PMID: 1541684 PMC442944

[ref58] Lambert C. Poyart C. Gruss A. Fouet A. (2022). FabT, a bacterial transcriptional repressor that limits futile fatty acid biosynthesis. Microbiol. Mol. Biol. Rev. 86:e0002922. doi: 10.1128/mmbr.00029-22, PMID: 35726719 PMC9491164

[ref59] Lebold K. M. Traber M. G. (2014). Interactions between α-tocopherol, polyunsaturated fatty acids, and lipoxygenases during embryogenesis. Free Radic. Biol. Med. 66, 13–19. doi: 10.1016/j.freeradbiomed.2013.07.039, PMID: 23920314 PMC3874081

[ref60] Lemos J. A. Palmer S. R. Zeng L. Wen Z. T. Kajfasz J. K. Freires I. A.. (2019). The biology of *Streptococcus mutans*. Microbiol. Spectr. 7. doi: 10.1128/microbiolspec.GPP3-0051-2018, PMID: 30657107 PMC6615571

[ref61] Liao Y. Brandt B. W. Li J. Crielaard W. Van Loveren C. Deng D. M. (2017). Fluoride resistance in *Streptococcus mutans*: a mini review. J. Oral Microbiol. 9:1344509. doi: 10.1080/20002297.2017.1344509, PMID: 28748043 PMC5508371

[ref62] Liebler D. C. Kling D. S. Reed D. J. (1986). Antioxidant protection of phospholipid bilayers by alpha-tocopherol. Control of alpha-tocopherol status and lipid peroxidation by ascorbic acid and glutathione. J. Biol. Chem. 261, 12114–12119. doi: 10.1016/S0021-9258(18)67210-2, PMID: 3745181

[ref63] Liu J. Zhang J. Guo L. Zhao W. Hu X. Wei X. (2017). Inactivation of a putative efflux pump (LmrB) in *Streptococcus mutans* results in altered biofilm structure and increased exopolysaccharide synthesis: implications for biofilm resistance. Biofouling 33, 481–493. doi: 10.1080/08927014.2017.1323206 28587519

[ref64] Livak K. J. Schmittgen T. D. (2001). Analysis of relative gene expression data using real-time quantitative PCR and the 2(-Delta Delta C(T)) method. Methods 25, 402–408. doi: 10.1006/meth.2001.1262 11846609

[ref65] Lu W. J. Lin H. J. Hsu P. H. Lin H. V. (2020). Determination of drug efflux pump efficiency in drug-resistant bacteria using MALDI-TOF MS. Antibiotics (Basel). 9:639. doi: 10.3390/antibiotics9100639, PMID: 32987695 PMC7598683

[ref66] Maione A. Buonanno A. Galdiero M. de Alteriis E. Petrillo F. Reibaldi M.. (2023). A re-purposing strategy: sub-lethal concentrations of an eicosanoid derived from the omega-3-polyunsaturated fatty acid Resolvin D1 affect dual species biofilms. Int. J. Mol. Sci. 24:12876. doi: 10.3390/ijms241612876, PMID: 37629056 PMC10454369

[ref67] Mao X. Auer D. L. Buchalla W. Hiller K. A. Maisch T. Hellwig E.. (2020). Cetylpyridinium chloride: mechanism of action, antimicrobial efficacy in biofilms, and potential risks of resistance. Antimicrob. Agents Chemother. 64, e00576-20. doi: 10.1128/AAC.00576-20, PMID: 32513792 PMC7526810

[ref68] Marsh P. D. (2010). Controlling the oral biofilm with antimicrobials. J. Dent. 38, S11–S15. doi: 10.1016/S0300-5712(10)70005-1 20621238

[ref69] Martelli G. Giacomini D. (2018). Antibacterial and antioxidant activities for natural and synthetic dual-active compounds. Eur. J. Med. Chem. 158, 91–105. doi: 10.1016/j.ejmech.2018.09.009 30205261

[ref70] Mattos-Graner R. O. Porter K. A. Smith D. J. Hosogi Y. Duncan M. J. (2006). Functional analysis of glucan binding protein B from *Streptococcus mutans*. J. Bacteriol. 188, 3813–3825. doi: 10.1128/JB.01845-05, PMID: 16707674 PMC1482924

[ref71] Müller H. D. Eick S. Moritz A. Lussi A. Gruber R. (2017). Cytotoxicity and antimicrobial activity of oral rinses *in vitro*. Biomed. Res. Int. 2017, 1–9. doi: 10.1155/2017/4019723 PMC5376431 28401154

[ref72] Nagayama K. Fujita K. Takashima Y. Ardin A. C. Ooshima T. Matsumoto-Nakano M. (2014). Role of ABC transporter proteins in stress responses of *Streptococcus mutans*. Oral Health Dent. Manag. 13, 359–365. PMID: 24984648

[ref73] Nakano K. Lapirattanakul J. Nomura R. Nemoto H. Alaluusua S. Grönroos L.. (2007). *Streptococcus mutans* clonal variation revealed by multilocus sequence typing. J. Clin. Microbiol. 45, 2616–2625. doi: 10.1128/JCM.02343-06, PMID: 17567784 PMC1951271

[ref74] Nasila K. Shijith K. Mohammed S. Ramya C. (2021). A review on cetylpyridinium chloride. Int. J. Res. Rev. 8, 439–445. doi: 10.52403/ijrr.20210453

[ref75] Oz H. S. (2017). Chronic inflammatory diseases and green tea polyphenols. Nutrients 9:561. doi: 10.3390/nu9060561, PMID: 28587181 PMC5490540

[ref76] Paiva C. N. Bozza M. T. (2014). Are reactive oxygen species always detrimental to pathogens? Antioxid. Redox Signal. 20, 1000–1037. doi: 10.1089/ars.2013.5447, PMID: 23992156 PMC3924804

[ref77] Paixão L. Rodrigues L. Couto I. Martins M. Fernandes P. de Carvalho C. C.. (2009). Fluorometric determination of ethidium bromide efflux kinetics in *Escherichia coli*. J. Biol. Eng. 3:18. doi: 10.1186/1754-1611-3-18, PMID: 19835592 PMC2774284

[ref78] Philip N. Suneja B. (2023). The revolutionary evolution in carious lesion management. J. Conserv. Dent. 26, 249–257. doi: 10.4103/jcd.jcd_54_23, PMID: 37398856 PMC10309123

[ref79] Piomelli D. (2014). More surprises lying ahead. The endocannabinoids keep us guessing. Neuropharmacology 76, 228–234. doi: 10.1016/j.neuropharm.2013.07.026, PMID: 23954677 PMC3855347

[ref80] Pozarowski P. Darzynkiewicz Z. (2004). Analysis of cell cycle by flow cytometry. Methods Mol. Biol. 281, 301–311. doi: 10.1385/1-59259-811-0:301 PMID: 15220539

[ref81] Qiu W. Zhou Y. Li Z. Huang T. Xiao Y. Cheng L.. (2020). Application of antibiotics/antimicrobial agents on dental caries. Biomed. Res. Int. 2020, 1–11. doi: 10.1155/2020/5658212 PMC7013294 32076608

[ref82] Reniere M. L. (2018). Reduce, induce, thrive: bacterial redox sensing during pathogenesis. J. Bacteriol. 200, e00128–e00118. doi: 10.1128/JB.00128-18 29891640 PMC6088161

[ref83] Sæbø I. P. Bjørås M. Franzyk H. Helgesen E. Booth J. A. (2023). Optimization of the hemolysis assay for the assessment of cytotoxicity. Int. J. Mol. Sci. 24:2914. doi: 10.3390/ijms24032914, PMID: 36769243 PMC9917735

[ref84] Schneider-Rayman M. Steinberg D. Sionov R. V. Friedman M. Shalish M. (2021). Effect of epigallocatechin gallate on dental biofilm of *Streptococcus mutans*: an *in vitro* study. BMC Oral Health 21:447. doi: 10.1186/s12903-021-01798-4, PMID: 34525984 PMC8444437

[ref85] Schumann J. (2016). It is all about fluidity: fatty acids and macrophage phagocytosis. Eur. J. Pharmacol. 785, 18–23. doi: 10.1016/j.ejphar.2015.04.057, PMID: 25987422

[ref86] Sendamangalam V. Choi O. K. Seo Y. Kim D.-S. (2011). Antimicrobial and antioxidant activities of polyphenols against *Streptococcus mutans*. Free Radicals Antioxidants 1, 48–55. doi: 10.5530/ax.2011.3.7

[ref87] Sharma N. Bhatia S. Sodhi A. S. Batra N. (2018). Oral microbiome and health. AIMS Microbiol. 4, 42–66. doi: 10.3934/microbiol.2018.1.42, PMID: 31294203 PMC6605021

[ref88] Shibata Y. Abiko Y. Ohishi T. Tamaya H. Takiguchi H. (1989). Free arachidonic acid source for PGE2 and TXB2 production in guinea pig peritoneal macrophages exposed to insoluble glucan from *Streptococcus mutans*. Int. J. Biochem. 21, 1043–1045. doi: 10.1016/0020-711X(89)90238-3, PMID: 2512188

[ref89] Song J. Choi B. Jin E. J. Yoon Y. Choi K. H. (2012). Curcumin suppresses *Streptococcus mutans* adherence to human tooth surfaces and extracellular matrix proteins. Eur. J. Clin. Microbiol. Infect. Dis. 31, 1347–1352. doi: 10.1007/s10096-011-1448-y, PMID: 22009290

[ref90] Stagos D. Portesis N. Spanou C. Mossialos D. Aligiannis N. Chaita E.. (2012). Correlation of total polyphenolic content with antioxidant and antibacterial activity of 24 extracts from Greek domestic *Lamiaceae* species. Food Chem. Toxicol. 50, 4115–4124. doi: 10.1016/j.fct.2012.08.033, PMID: 22939934

[ref91] Standard I. Biological evaluation of medical devices—Part 5: Tests for in vitro cytotoxicity. Geneve, Switzerland: International Organization for Standardization (2009)

[ref92] Suntharalingam P. Cvitkovitch D. G. (2005). Quorum sensing in streptococcal biofilm formation. Trends Microbiol. 13, 3–6. doi: 10.1016/j.tim.2004.11.009 15639624

[ref93] Suwa M. Morii I. Kino M. (2020). Fatty acid treatment with pure omega-3 eicosapentaenoic acid ethyl ester for patients with cardiovascular diseases: differences between branded (EPADEL®) and generic products. Food Nutr. Sci. 11, 887–898. doi: 10.4236/fns.2020.1110062

[ref94] Takahashi N. Nyvad B. (2011). The role of bacteria in the caries process: ecological perspectives. J. Dent. Res. 90, 294–303. doi: 10.1177/0022034510379602 20924061

[ref95] Takashima Y. Fujita K. Ardin A. C. Nagayama K. Nomura R. Nakano K.. (2015). Characterization of the dextran-binding domain in the glucan-binding protein C of *Streptococcus mutans*. J. Appl. Microbiol. 119, 1148–1157. doi: 10.1111/jam.12895, PMID: 26176557

[ref96] Tallima H. El Ridi R. (2018). Arachidonic acid: physiological roles and potential health benefits - a review. J. Adv. Res. 11, 33–41. doi: 10.1016/j.jare.2017.11.004, PMID: 30034874 PMC6052655

[ref97] Tallima H. Hanna V. S. El Ridi R. (2020). Arachidonic acid is a safe and efficacious Schistosomicide, and an Endoschistosomicide in natural and experimental infections, and cysteine peptidase vaccinated hosts. Front. Immunol. 11:609994. doi: 10.3389/fimmu.2020.609994, PMID: 33281832 PMC7705376

[ref98] Wang S. Wang P. Liu J. Yang C. Wang Q. Su M.. (2022). Antibiofilm activity of essential fatty acids against *Candida albicans* from vulvovaginal candidiasis and bloodstream infections. Infect Drug Resist. 15, 4181–4193. doi: 10.2147/IDR.S373991, PMID: 35946033 PMC9357398

[ref99] Webb A. J. Homer K. A. Hosie A. H. (2008). Two closely related ABC transporters in *Streptococcus mutans* are involved in disaccharide and/or oligosaccharide uptake. J. Bacteriol. 190, 168–178. doi: 10.1128/JB.01509-07, PMID: 17965163 PMC2223742

[ref100] Webb A. J. Hosie A. H. (2006). A member of the second carbohydrate uptake subfamily of ATP-binding cassette transporters is responsible for ribonucleoside uptake in *Streptococcus mutans*. J. Bacteriol. 188, 8005–8012. doi: 10.1128/JB.01101-06, PMID: 16997965 PMC1698198

[ref101] Whittle E. E. Legood S. W. Alav I. Dulyayangkul P. Overton T. W. Blair J. M. A. (2019). Flow cytometric analysis of efflux by dye accumulation. Front. Microbiol. 10:2319. doi: 10.3389/fmicb.2019.02319, PMID: 31636625 PMC6787898

[ref102] Wolfson G. Sionov R. V. Smoum R. Korem M. Polacheck I. Steinberg D. (2023). Anti-bacterial and anti-biofilm activities of anandamide against the cariogenic *Streptococcus mutans*. Int. J. Mol. Sci. 24:6177. doi: 10.3390/ijms24076177, PMID: 37047147 PMC10094667

[ref103] Wong A. Subar P. E. Young D. A. (2017). Dental caries: an update on dental trends and therapy. Adv. Pediatr. Infect. Dis. 64, 307–330. doi: 10.1016/j.yapd.2017.03.011 28688595

[ref104] Wright P. P. Ramachandra S. S. (2022). Quorum sensing and quorum quenching with a focus on cariogenic and periodontopathic oral biofilms. Microorganisms 10:1783. doi: 10.3390/microorganisms10091783, PMID: 36144385 PMC9503171

[ref105] Wu-Yuan C. D. Tai S. Slade H. D. (1979). Properties of *Streptococcus mutans* grown in a synthetic medium: binding of glucosyltransferase and *in vitro* adherence, and binding of dextran/glucan and glycoprotein and agglutination. Infect. Immun. 23, 600–608. doi: 10.1128/iai.23.3.600-608.1979, PMID: 457252 PMC414208

[ref106] Yamada T. Carlsson J. (1973). Phosphoenolpyruvate carboxylase and ammonium metabolism in oral streptococci. Arch. Oral Biol. 18, 799–812. doi: 10.1016/0003-9969(73)90051-4, PMID: 4516182

[ref107] Yamashita Y. Shibata Y. Nakano Y. Tsuda H. Kido N. Ohta M.. (1999). A novel gene required for rhamnose-glucose polysaccharide synthesis in *Streptococcus mutans*. J. Bacteriol. 181, 6556–6559. doi: 10.1128/JB.181.20.6556-6559.1999, PMID: 10515952 PMC103797

[ref108] Yazicioglu O. Ucuncu M. K. Guven K. (2023). Ingredients in commercially available mouthwashes: a review. Int. Dent. J. S0020-6539, 437–439. doi: 10.1016/j.identj.2023.08.004 PMC10988267 37709645

[ref109] Zamakhaeva S. Chaton C. T. Rush J. S. Ajay Castro S. Kenner C. W. Yarawsky A. E.. (2021). Modification of cell wall polysaccharide guides cell division in *Streptococcus mutans*. Nat. Chem. Biol. 17, 878–887. doi: 10.1038/s41589-021-00803-9, PMID: 34045745 PMC8403489

[ref110] Zeng H. Liu J. Ling J. (2017). Efflux inhibitor suppresses *Streptococcus mutans* virulence properties. FEMS Microbiol. Lett. 364:364. doi: 10.1093/femsle/fnx033 28175292

[ref111] Zhang Q. Ma Q. Wang Y. Wu H. Zou J. (2021). Molecular mechanisms of inhibiting glucosyltransferases for biofilm formation in *Streptococcus mutans*. Int. J. Oral Sci. 13:30. doi: 10.1038/s41368-021-00137-1, PMID: 34588414 PMC8481554

[ref112] Zhao X. Drlica K. (2014). Reactive oxygen species and the bacterial response to lethal stress. Curr. Opin. Microbiol. 21, 1–6. doi: 10.1016/j.mib.2014.06.008, PMID: 25078317 PMC4254325

[ref113] Zheng T. Jing M. Gong T. Yan J. Wang X. Xu M.. (2023). Regulatory mechanisms of exopolysaccharide synthesis and biofilm formation in *Streptococcus mutans*. J. Oral Microbiol. 15:2225257. doi: 10.1080/20002297.2023.2225257, PMID: 37346997 PMC10281425

[ref114] Zheng C. J. Yoo J. S. Lee T. G. Cho H. Y. Kim Y. H. Kim W. G. (2005). Fatty acid synthesis is a target for antibacterial activity of unsaturated fatty acids. FEBS Lett. 579, 5157–5162. doi: 10.1016/j.febslet.2005.08.028 16146629

