# Supplementary material for: Anti-bacterial and anti-biofilm activities of arachidonic acid against the cariogenic bacterium Streptococcus mutans
Source: Front Microbiol. 2024 Feb 26;15:1333274. doi: 10.3389/fmicb.2024.1333274 (PMC11002910; doi:10.3389/fmicb.2024.1333274)
Supplement: Supplementary file 1 [file Data_Sheet_1.PDF]

Supplementary Material

Anti-Bacterial and Anti-Biofilm Activities of Arachidonic Acid against the Cariogenic Bacterium *Streptococcus mutans*

Manoj Chamlagain, Jieni Hu, Ronit Vogt Sionov and Doron Steinberg

Supplementary Table S1: Sequences of primers used for real-time PCR.

Gene symbol	Forward Primer	Reverse Primer	Reference
atlA	GCTCACTATACTTCTGCGGC	AACCTTGACGCCTCATCTCA	This paper
atpD	CGTGCTCTCTCGCCTGAAATAG	ACTCACGATAACGCTGCAAGAC	(1)
brpA	GGAGGAGCTGCATCAGGATTC	AACTCCAGCACATCCAGCAAG	(1)
clpX	CCGTGCCAAACGTGCTTTAGC	TCCAGAACCTGTCGGTCCAATC	(2)
dnaK	GCAGGTCAAGAGGGAGCTCA	CCGCCCTTGTCTGAGAATC	(1)
fabD	CATTGCTGTCGCTTCTGGTTTG	GCCTGCGTTTGTTCTGTTTGTG	(2)
fabM	ACTGATTAATGCCAATGGGAAAGTC	TGCGAACAAGAGATTGTACATCATC	(1)
ftf	AAATATGAAGGCGGCTACAACG	CTTCACCAGTCTTAGCATCCTGAA	(1)
ftsZ	CAACCAAGAGCACAACAGCAAG	ACGACGAAGATTCCAATCGCC	(1)
gbpB	AGGGCAATGTACTTGGGGTG	TTTGGCCACCTTGAACACCT	(1)
groEL	CCAGGAGCTTTGACTGCGAC	TTGCGGATGATGATGTAGATGGT	(1)
gtfB	AGCAATGCAGCCAATCTACAAAT	ACGAACTTTGCCGTTATTGTCA	(1)
gtfC	GGTTTAACGTCAAAATTAGCTGTATT	CTCAACCAACCGCCACTGTT	(1)
gyrA	TACAGGTGATGTCATGGGTAAATAC	CCGGGTAGTACTTCCATTAGGTCAC	(1)
lmrB	GTTCCTAAGGCACCGGCAAA	CGCCGGTTTTGTGTTACTGC	(3)
luxS	ACTGTTCCCCTTTTGGCTGTC	AACTTGCTTTGATGACTGTGGC	(1)
nox	GGGTTGTGGAATGGCACTTTGG	CAATGGCTGTCACTGGCGATTC	(1)
relA	ACAAAAAGGGTATCGTCCGTACAT	AATCACGCTTGGTATTGCTAATTG	(1)
rgpG	CGGTCGCCATTATCAGACGAAC	CGATGAGTGAATCCCATAGCAAG	This paper
sodA	GCAGTGCTAAGACTCCCGAATC	TTGCGGAAGTGTGAGATTGGC	(1)
SMU_1286c	TATGCCCTGCATGATCCGTC	TGCAGAACAGACAGCTTGGT	This paper
spaP	GACTTTGGTAATGGTTATGCATCAA	TTTGTATCAGCCGGATCAAGTG	(1)

1. Wolfson G, Sionov RV, Smoum R, Korem M, Polacheck I, Steinberg D. Anti-bacterial and anti-biofilm activities of anandamide against the cariogenic *Streptococcus mutans*. Int J Mol Sci. 2023;24(7):6177.
2. Kajfasz JK, Rivera-Ramos I, Abranches J, Martinez AR, Rosalen PL, Derr AM, *et al.* Two Spx proteins modulate stress tolerance, survival, and virulence in *Streptococcus mutans.* J Bacteriol. 2010;192(10):2546-56.
3. Liu J, Zhang J, Guo L, Zhao W, Hu X, Wei X. Inactivation of a putative efflux pump (LmrB) in *Streptococcus mutans* results in altered biofilm structure and increased exopolysaccharide synthesis: Implications for biofilm resistance. Biofouling. 2017;33(6):481-93.

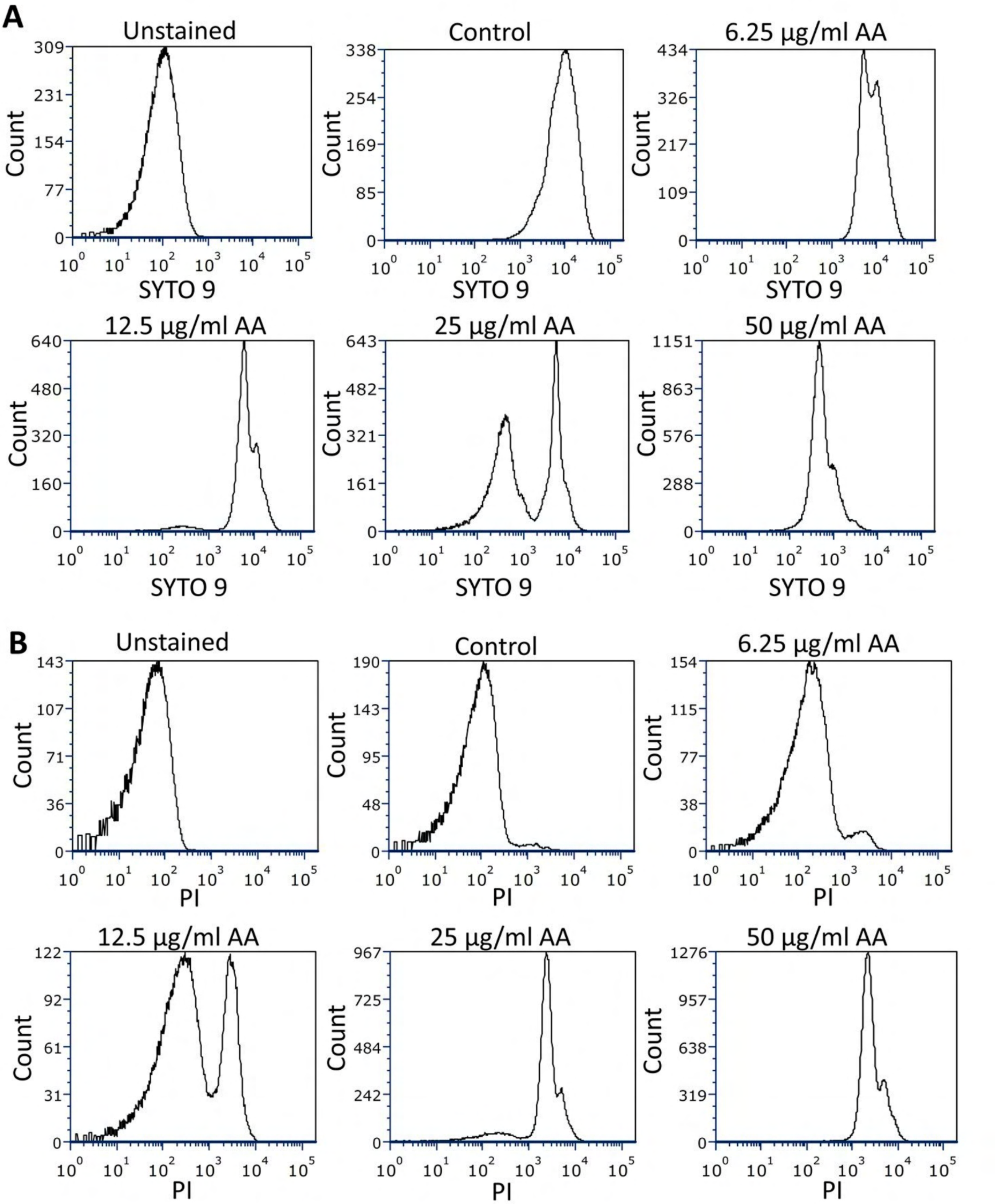

Supplementary Figure S1: A-B. Histograms of SYTO 9 (**A**) and PI (**B**) of unstained, control bacteria and *S. mutans* after a 2 h incubation with various concentrations of arachidonic acid (AA).

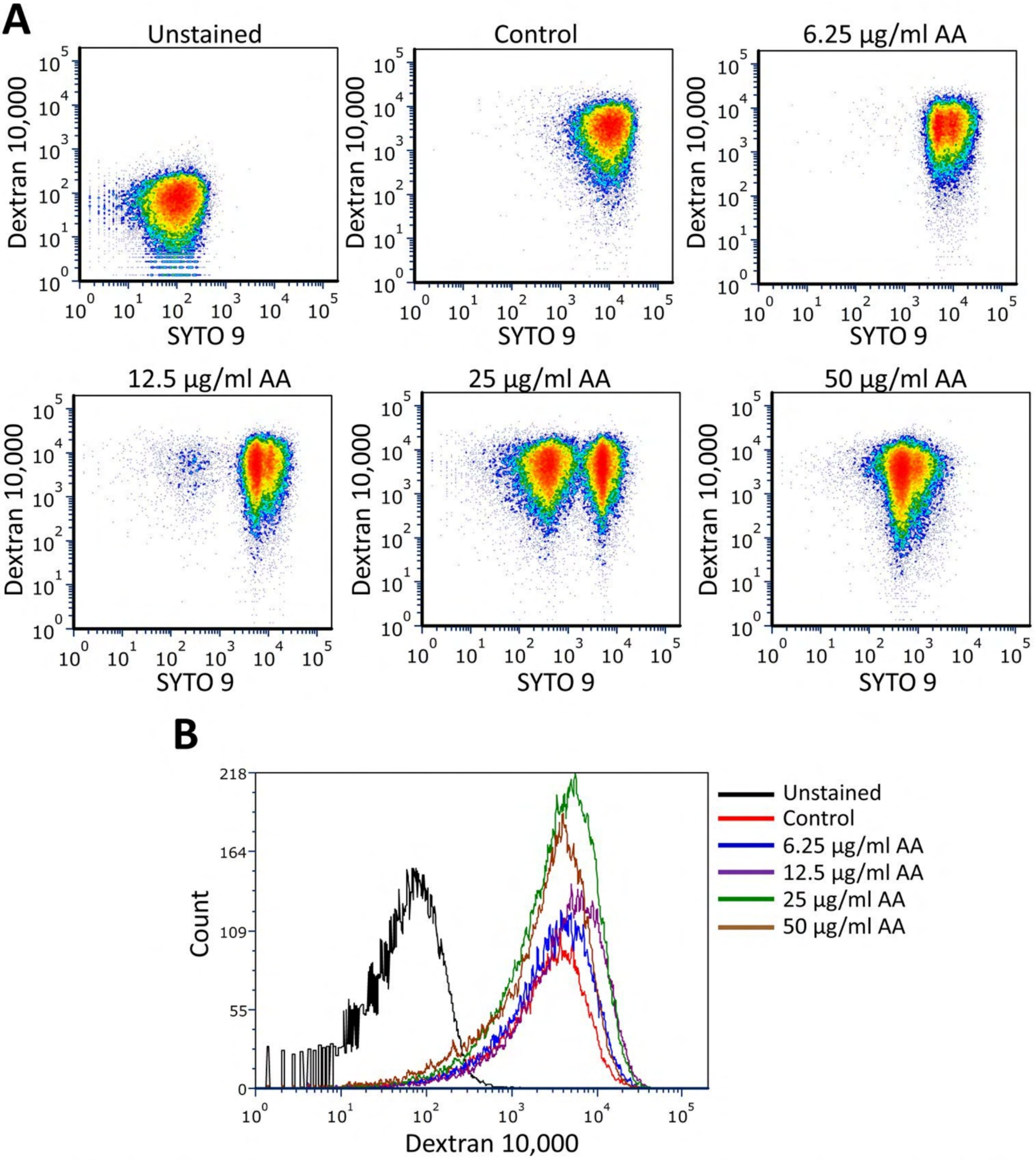

Supplementary Figure S2: A. Flow cytometry density plots of Alexafluor^{647}-labeled Dextran 10,000 versus SYTO 9 of unstained, control and treated *S. mutans* after a 2 h incubation with arachidonic acid (AA). **B.** Histogram of Dextran 10,000 staining of control and 2 h AA-treated bacteria. 50,000 events were collected for each sample. N=3.

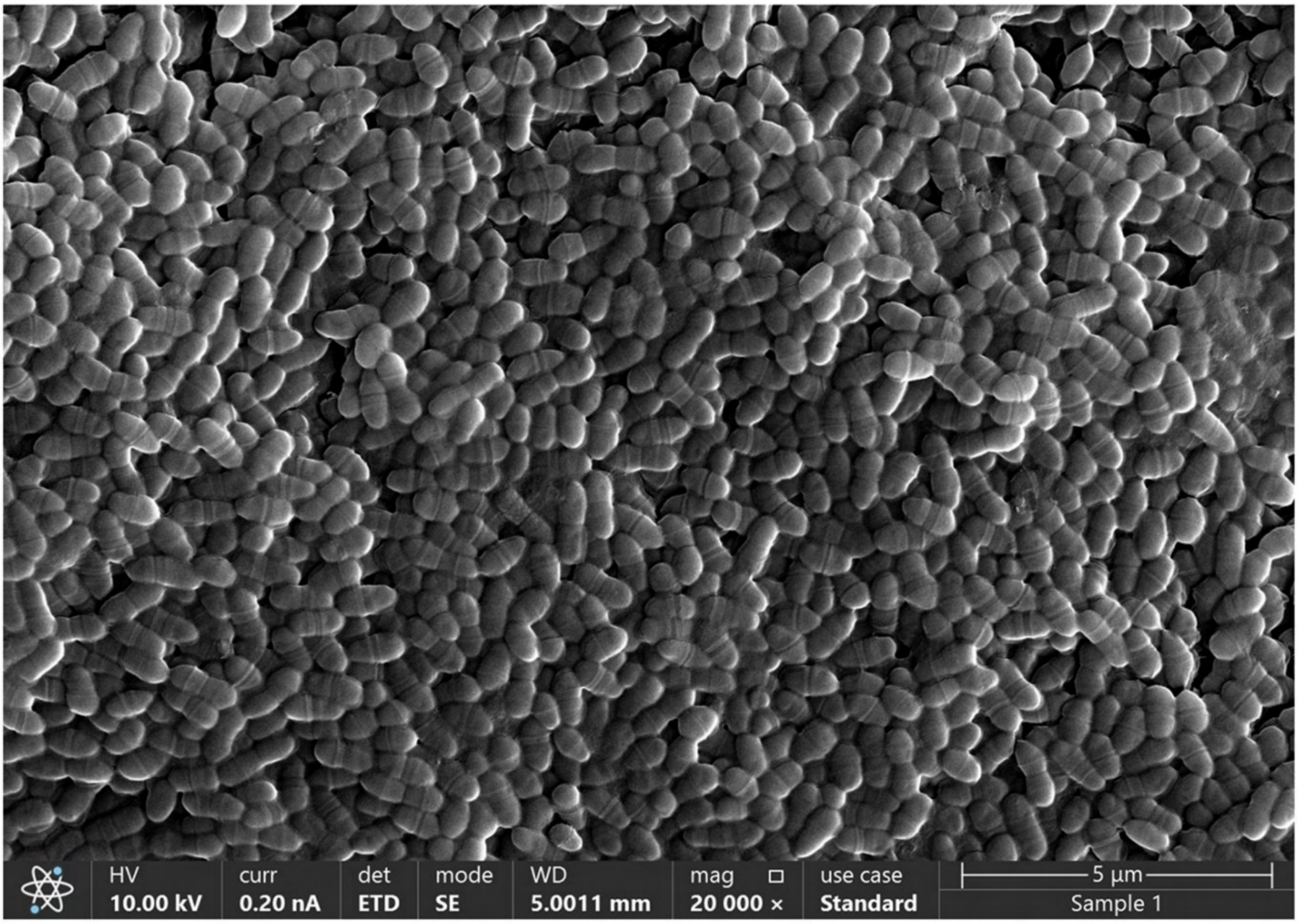

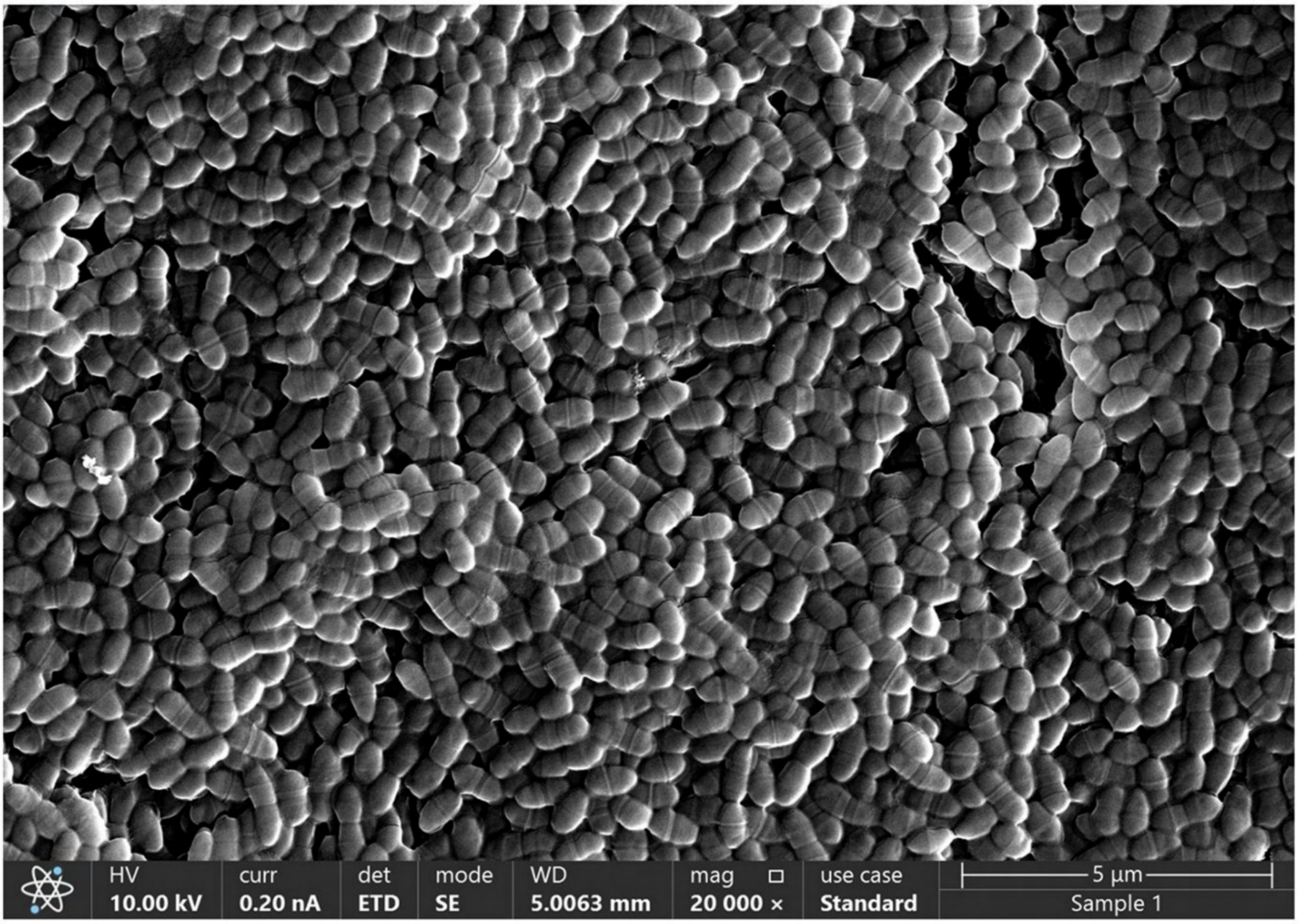

Supplementary Figure S3A: Uncropped HR-SEM images of control *S. mutans* bacteria. Magnification: × 20,000.

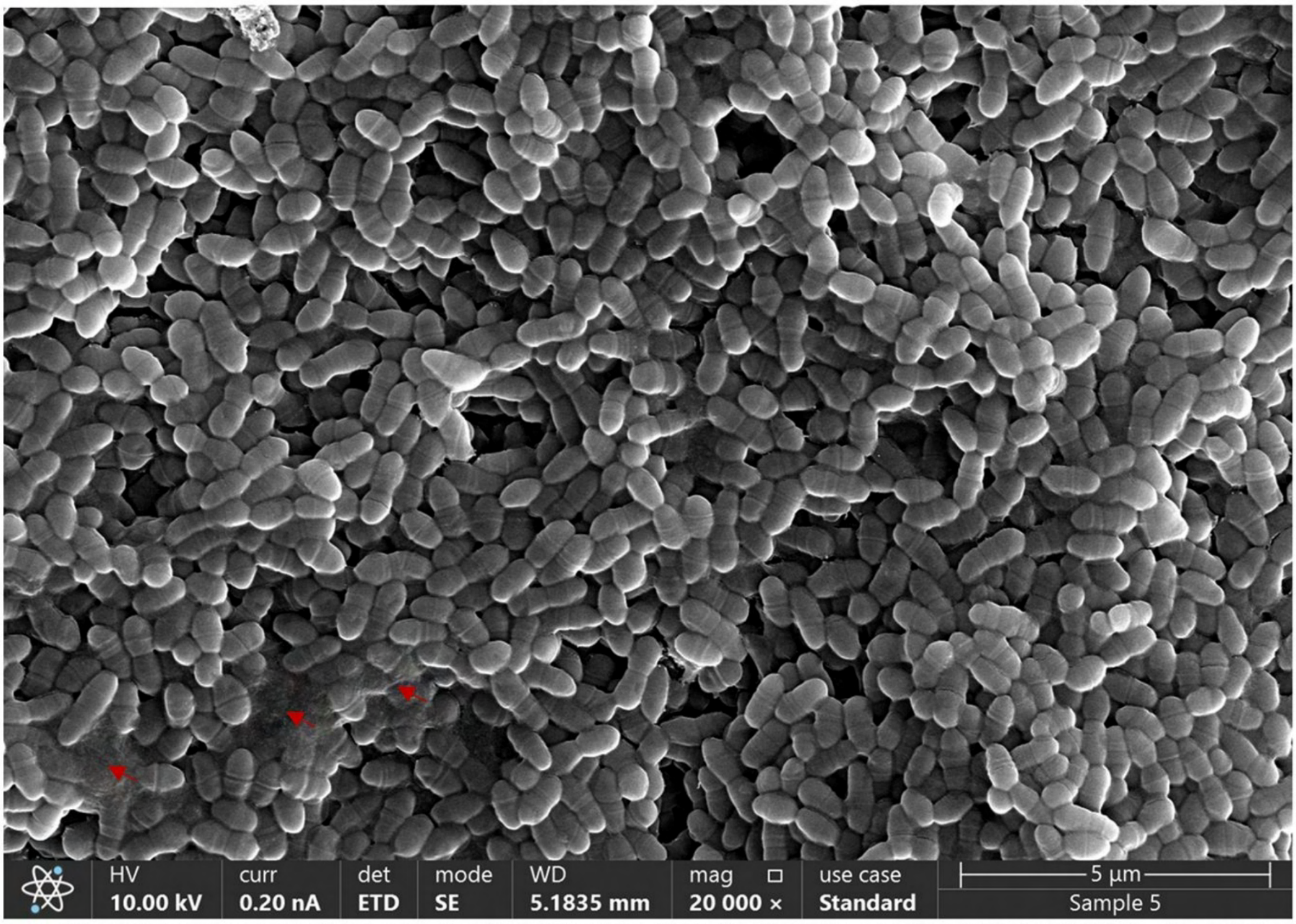

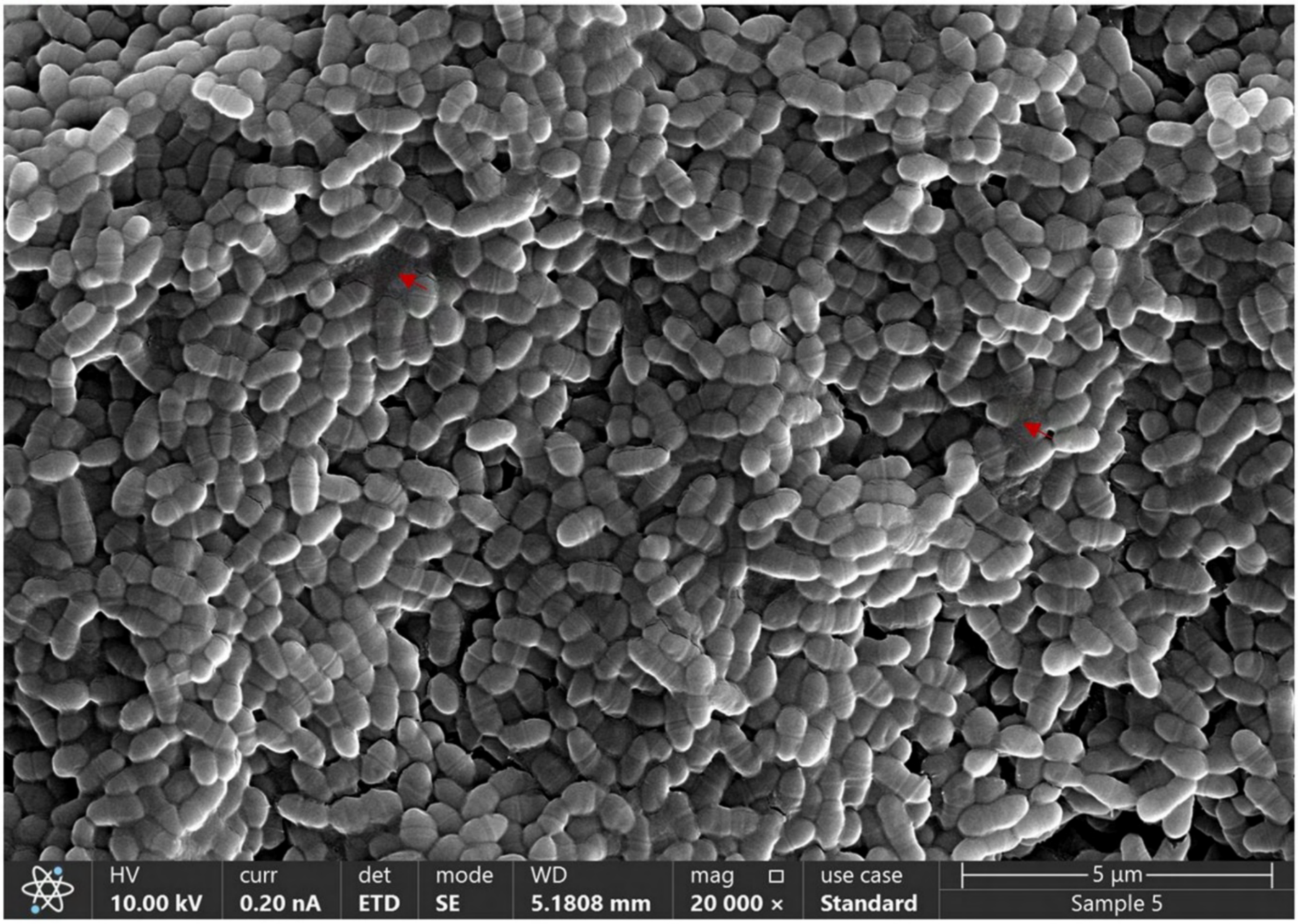

Supplementary Figure S3B: Uncropped HR-SEM images of *S. mutans* exposed to 6.25 µg/mL arachidonic acid (AA) for 2 h. Red arrows point to debris from exploded bacteria. Magnification: × 20,000.

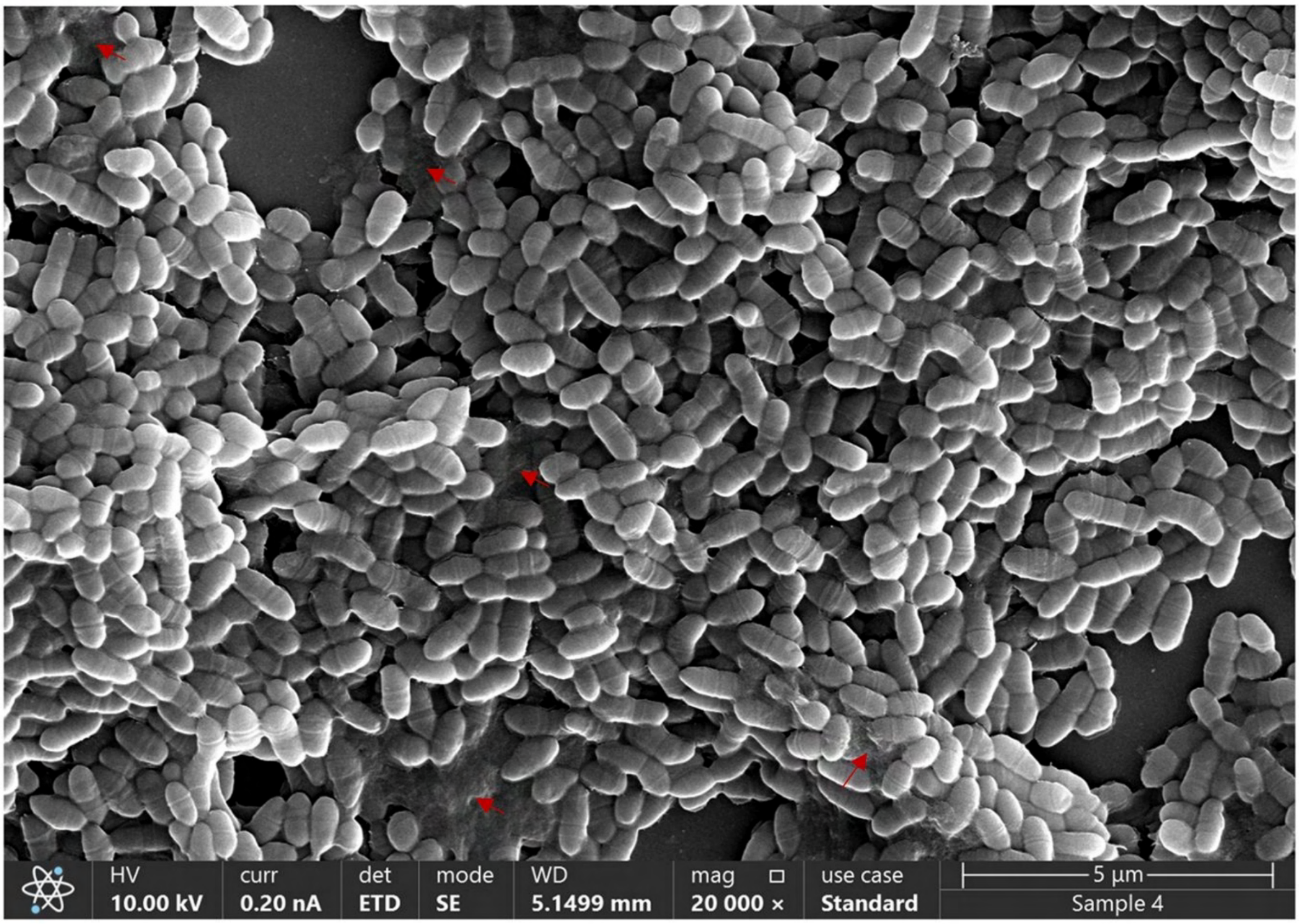

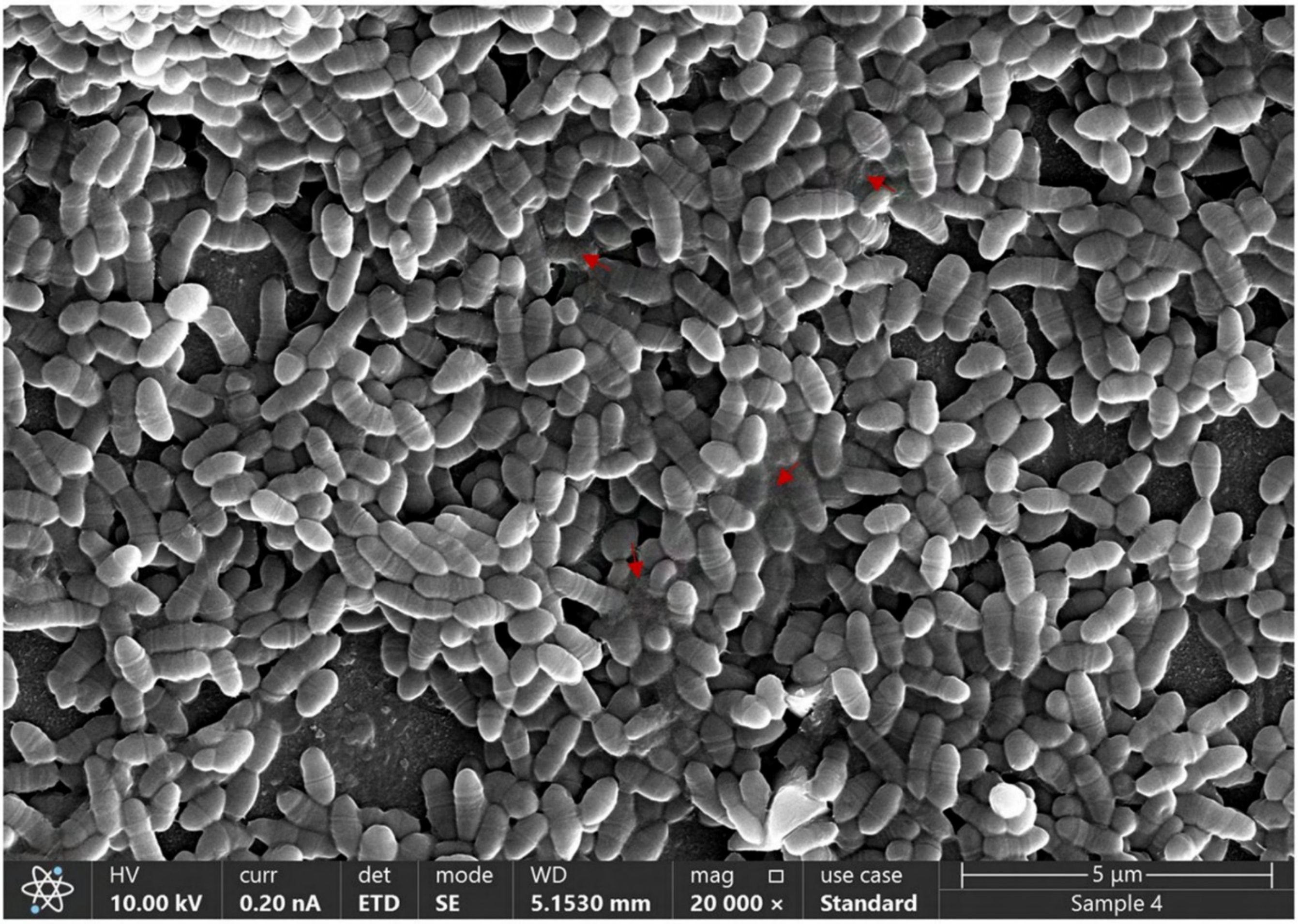

Supplementary Figure S3C: Uncropped HR-SEM images of *S. mutans* exposed to 12.5 µg/mL arachidonic acid (AA) for 2 h. Red arrows point to debris from exploded bacteria. Magnification: × 20,000.

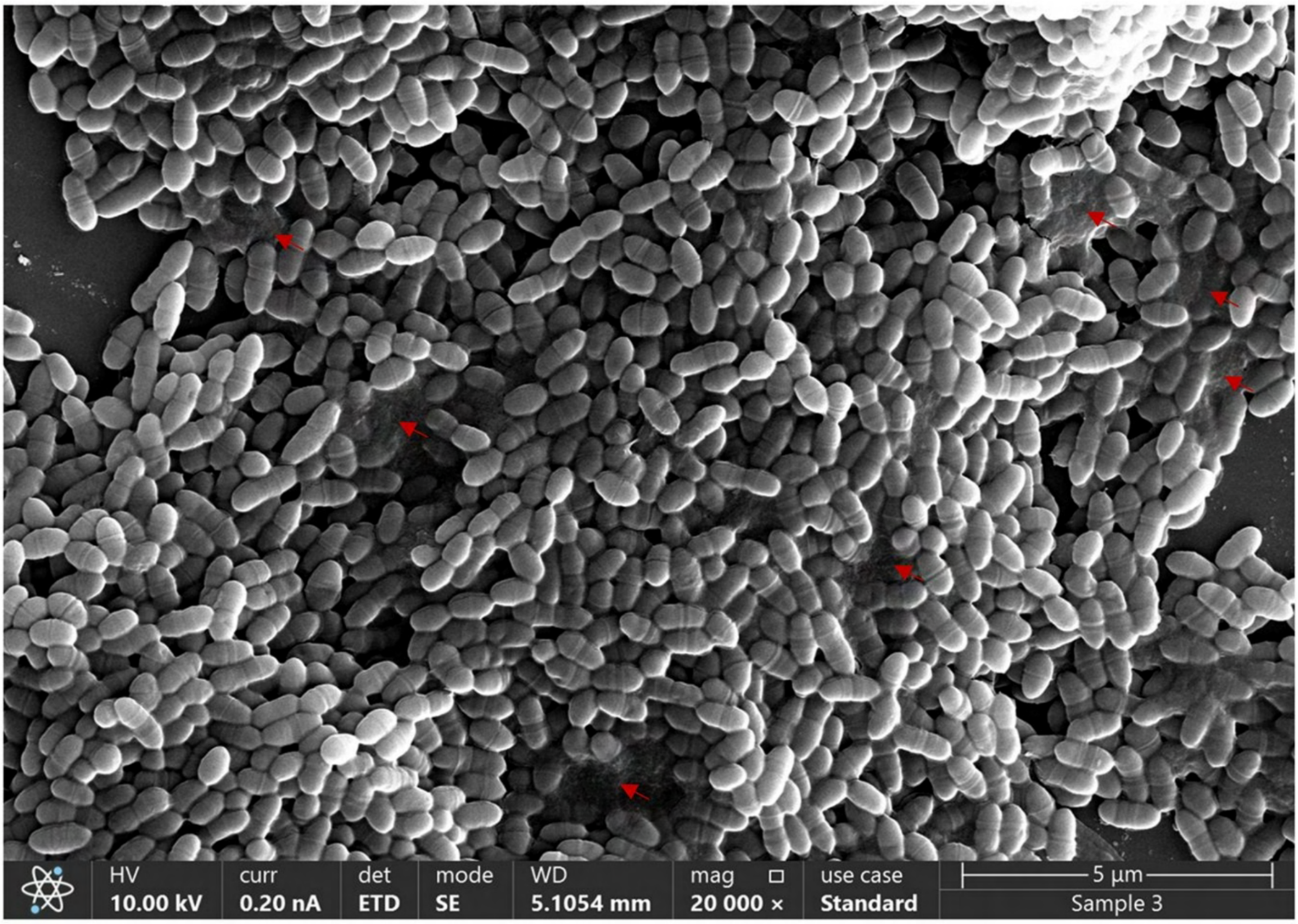

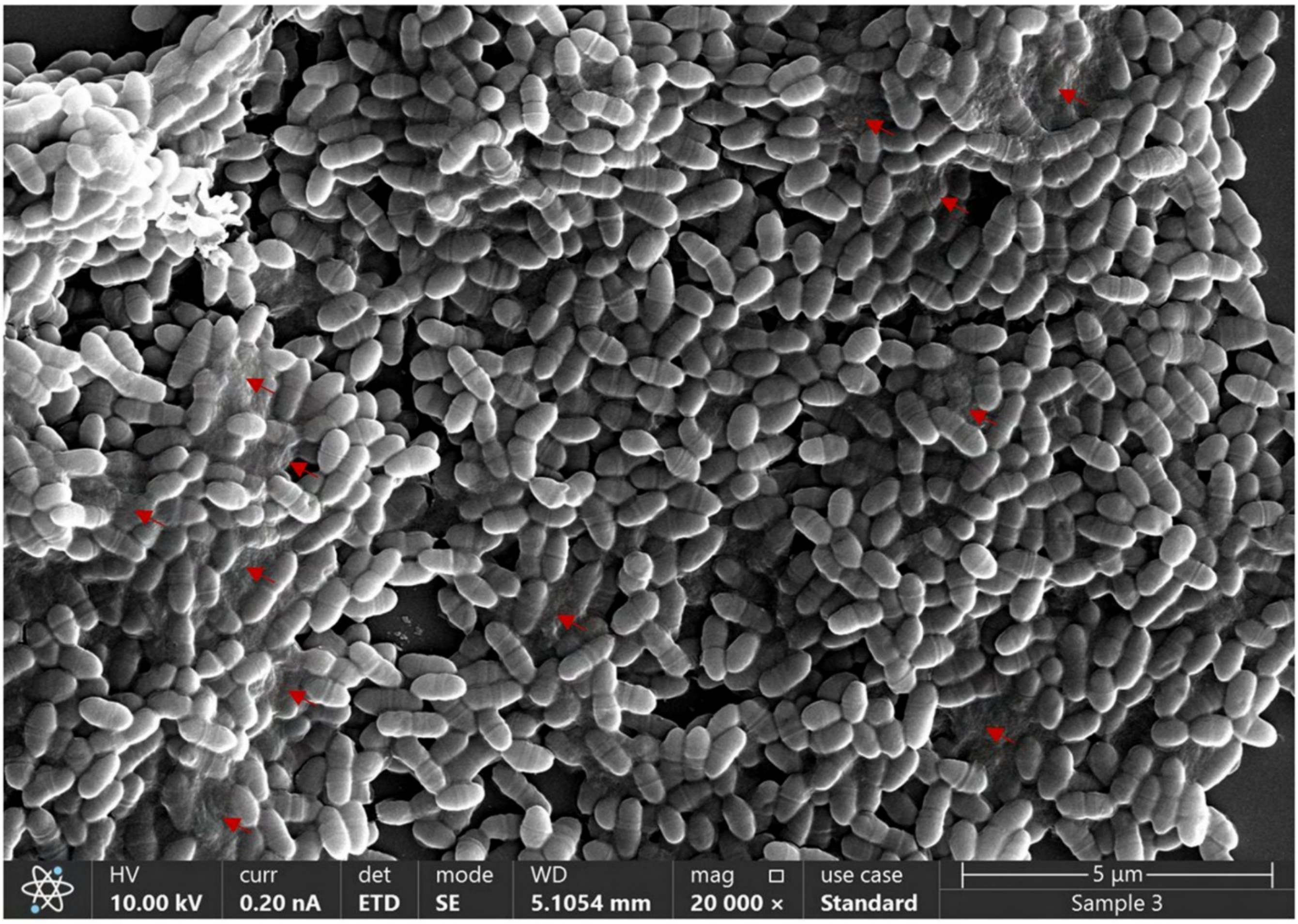

Supplementary Figure S3D: Uncropped HR-SEM images of *S. mutans* exposed to 25 µg/mL arachidonic acid (AA) for 2 h. Red arrows point to debris from exploded bacteria. Magnification: × 20,000.

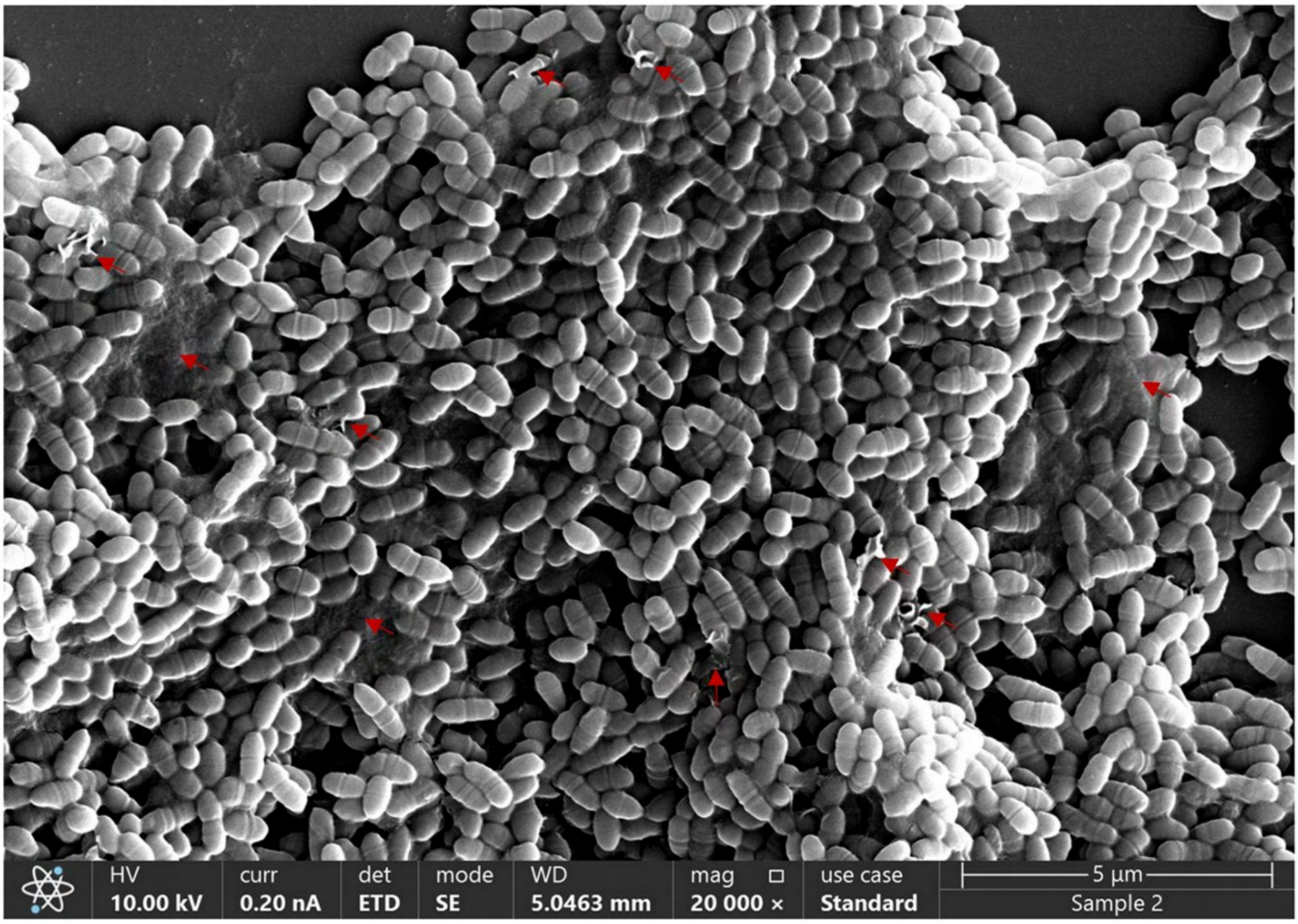

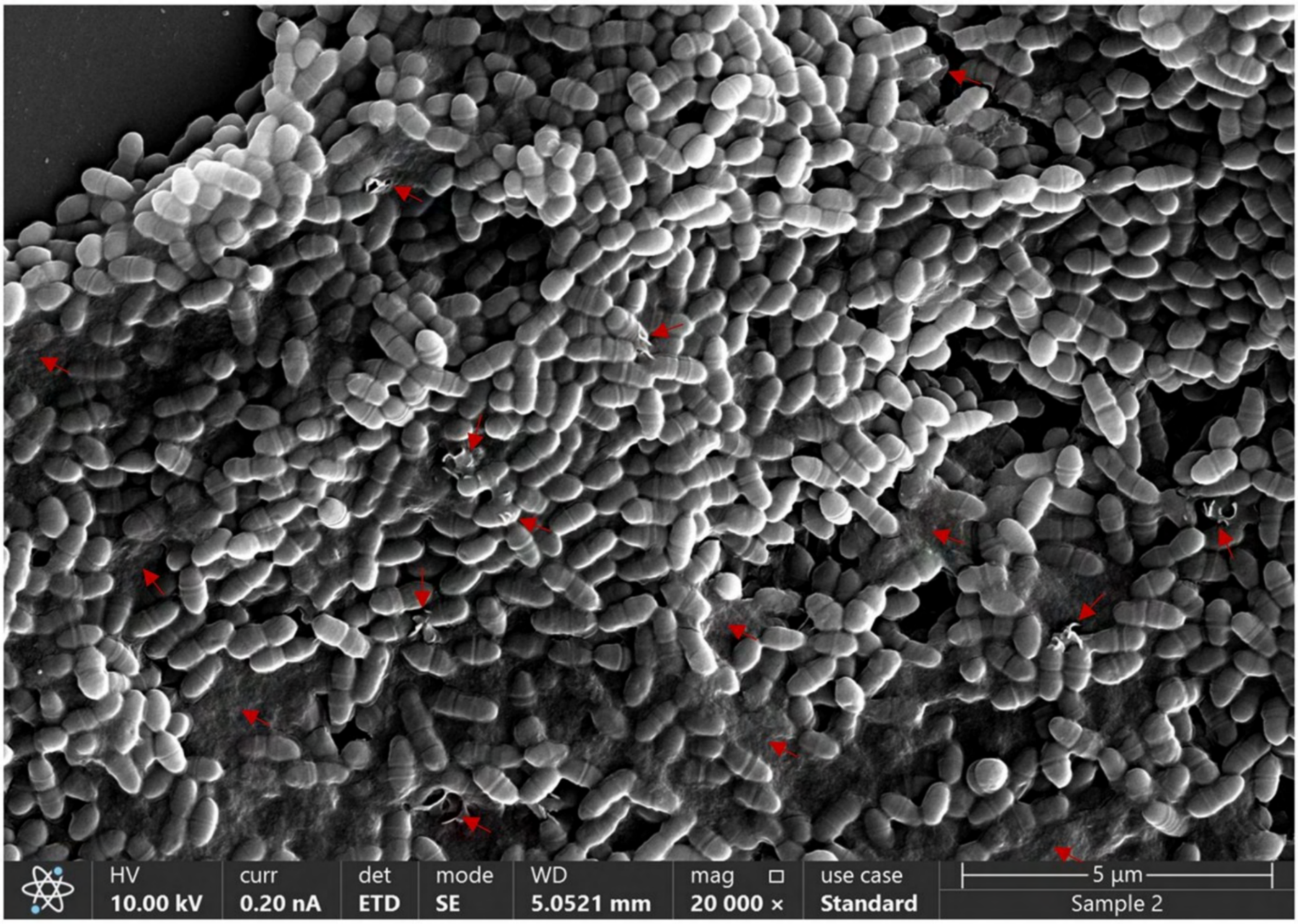

Supplementary Figure S3E: Uncropped HR-SEM images of *S. mutans* exposed to 50 µg/mL arachidonic acid (AA) for 2 h. Red arrows point to debris from exploded bacteria. Magnification: × 20,000.

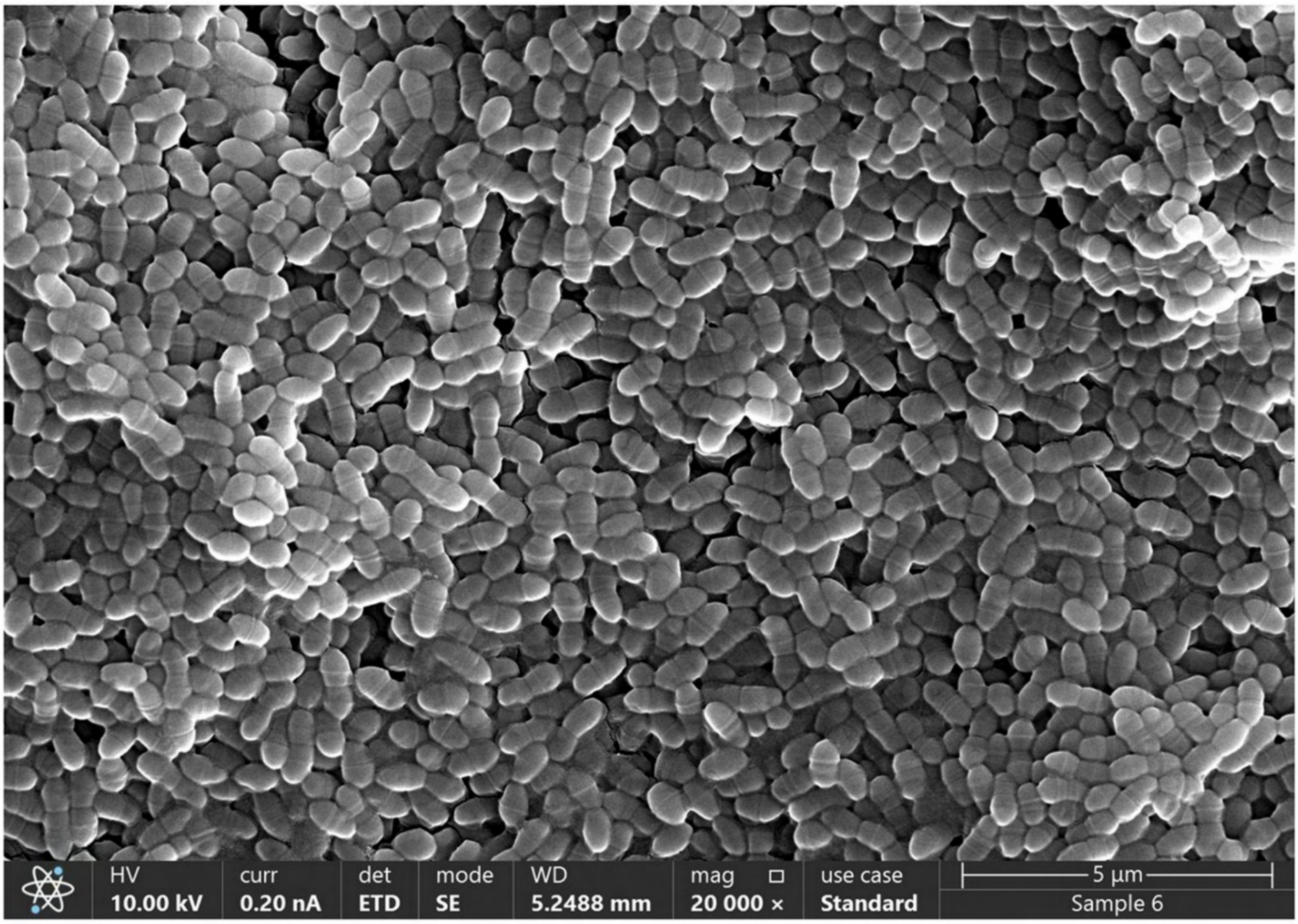

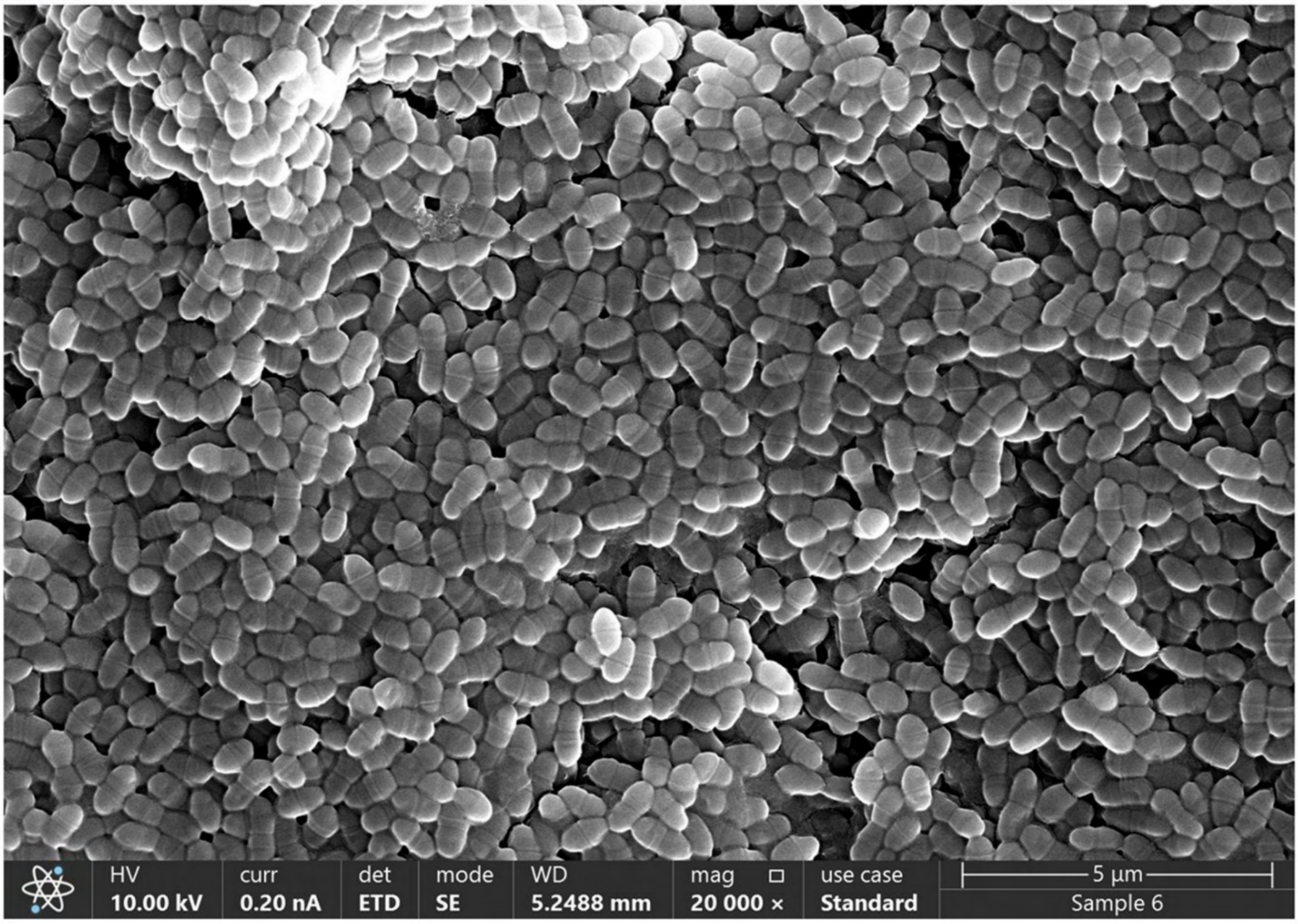

Supplementary Figure S3F: Uncropped HR-SEM images of *S. mutans* exposed to 0.1% ethanol for 2 h. Magnification: × 20,000.

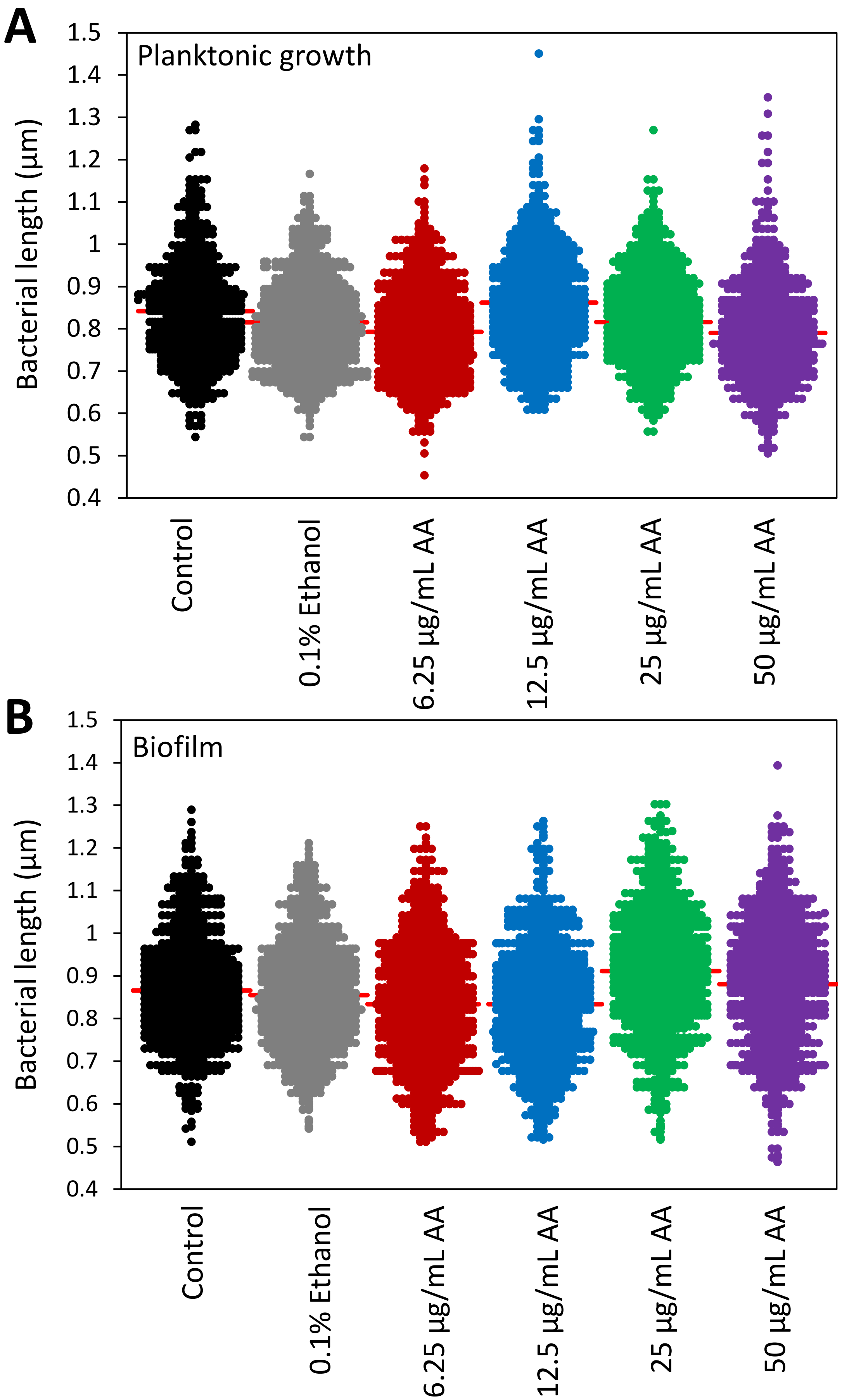

Supplementary Figure S4: **A.** The length of planktonic growing *S. mutans* in control and AA-treated samples as measured on 10-12 different HR-SEM images (×20,000-50,000) from three individiual samples using the Image J software. N=520-550 for each treatment group. The red line shows the median bacterial length. **B.** The length of *S. mutans* in biofilms of control and AA-treated samples as measured on 10-12 different HR-SEM images (×20,000) from three individiual samples using the Image J software. N=620-650 for each treatment group. The red line shows the median bacterial length.

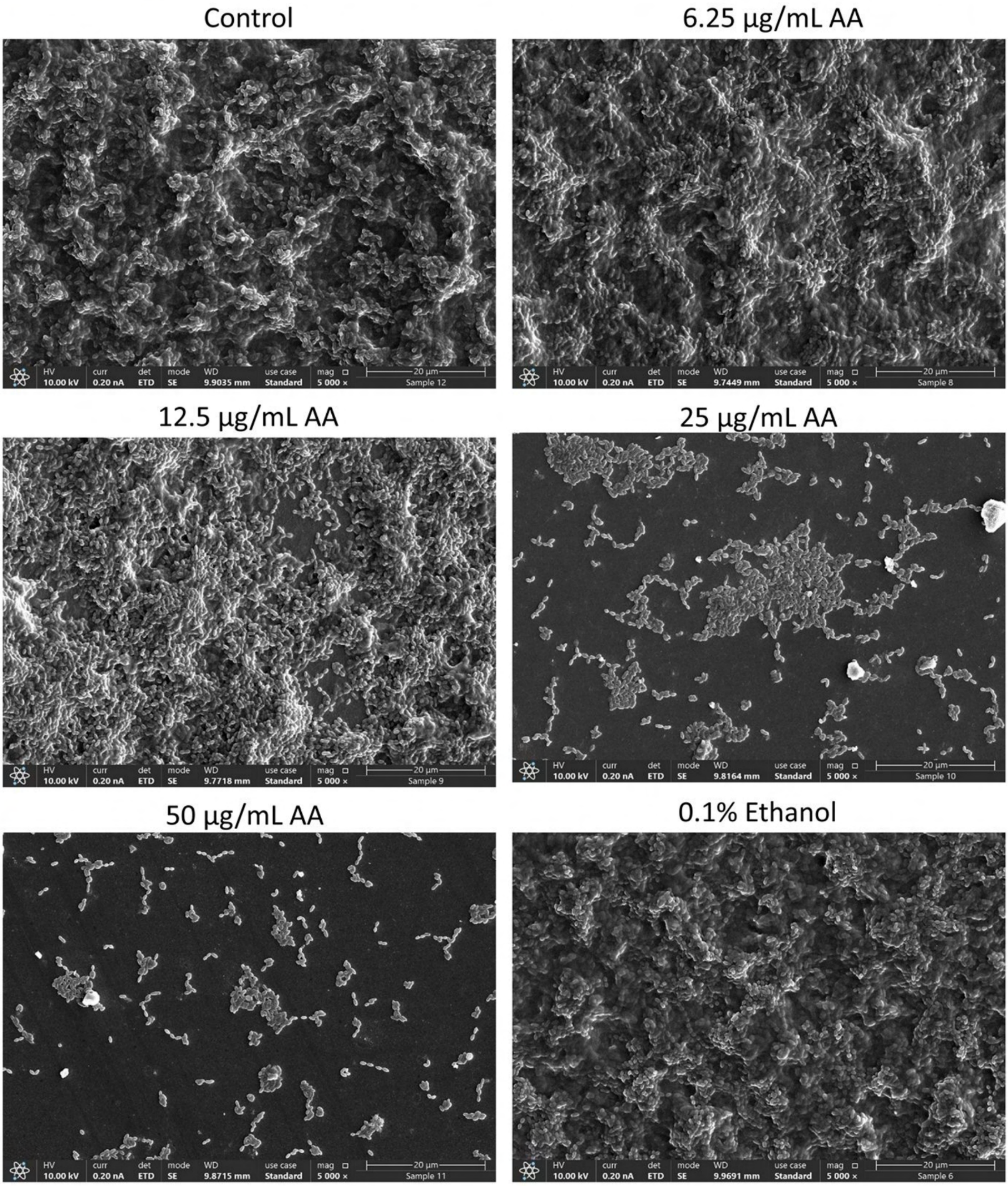

Supplementary Figure S5 : Panoramic HR-SEM images of biofilms formed by control *S. mutans* and bacteria that have been exposed to AA for 24 h. Magnification × 5000.

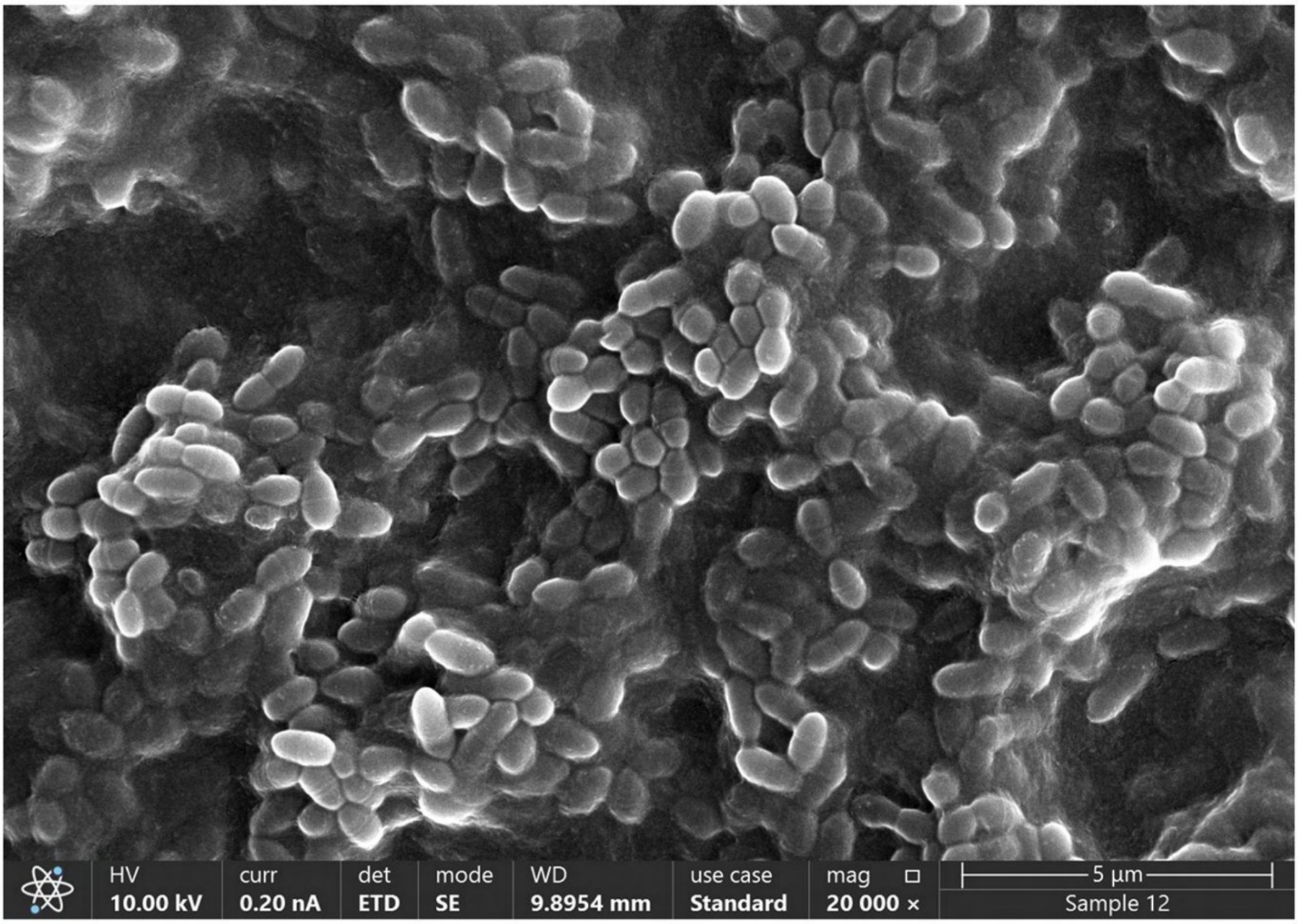

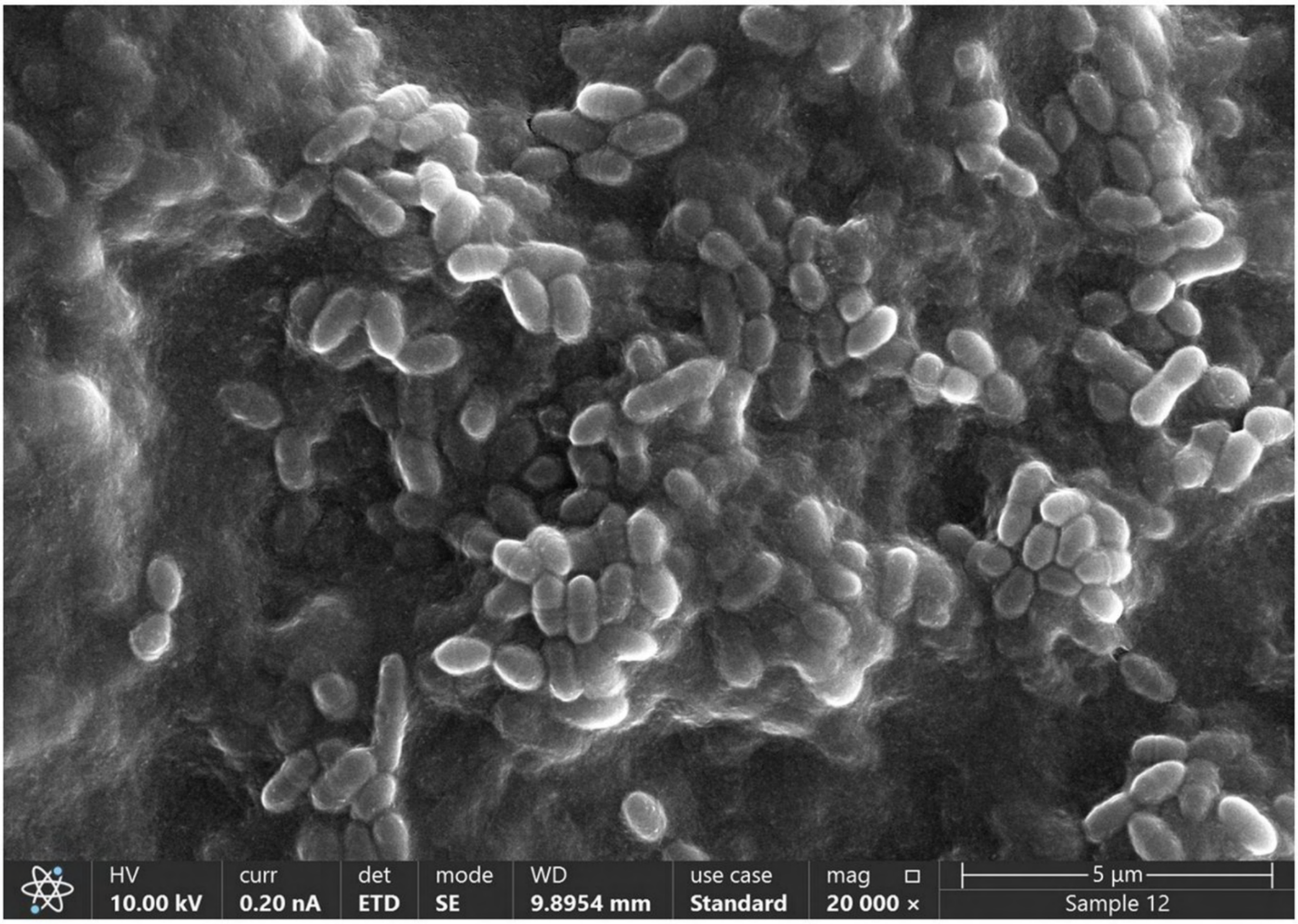

Supplementary Figure S6A : Uncropped HR-SEM images of biofilms formed by control *S. mutans* for 24 h. Magnification × 20,000.

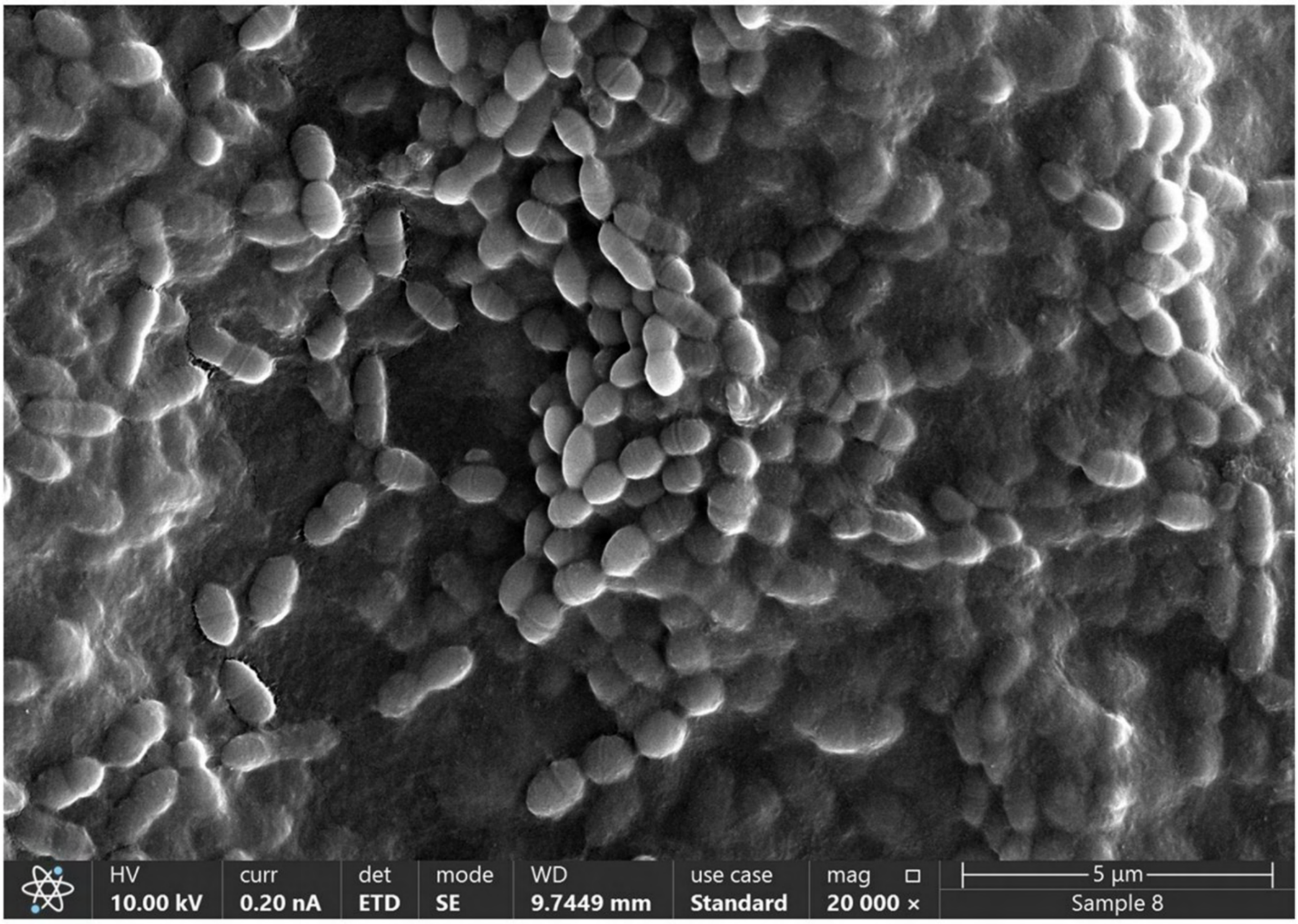

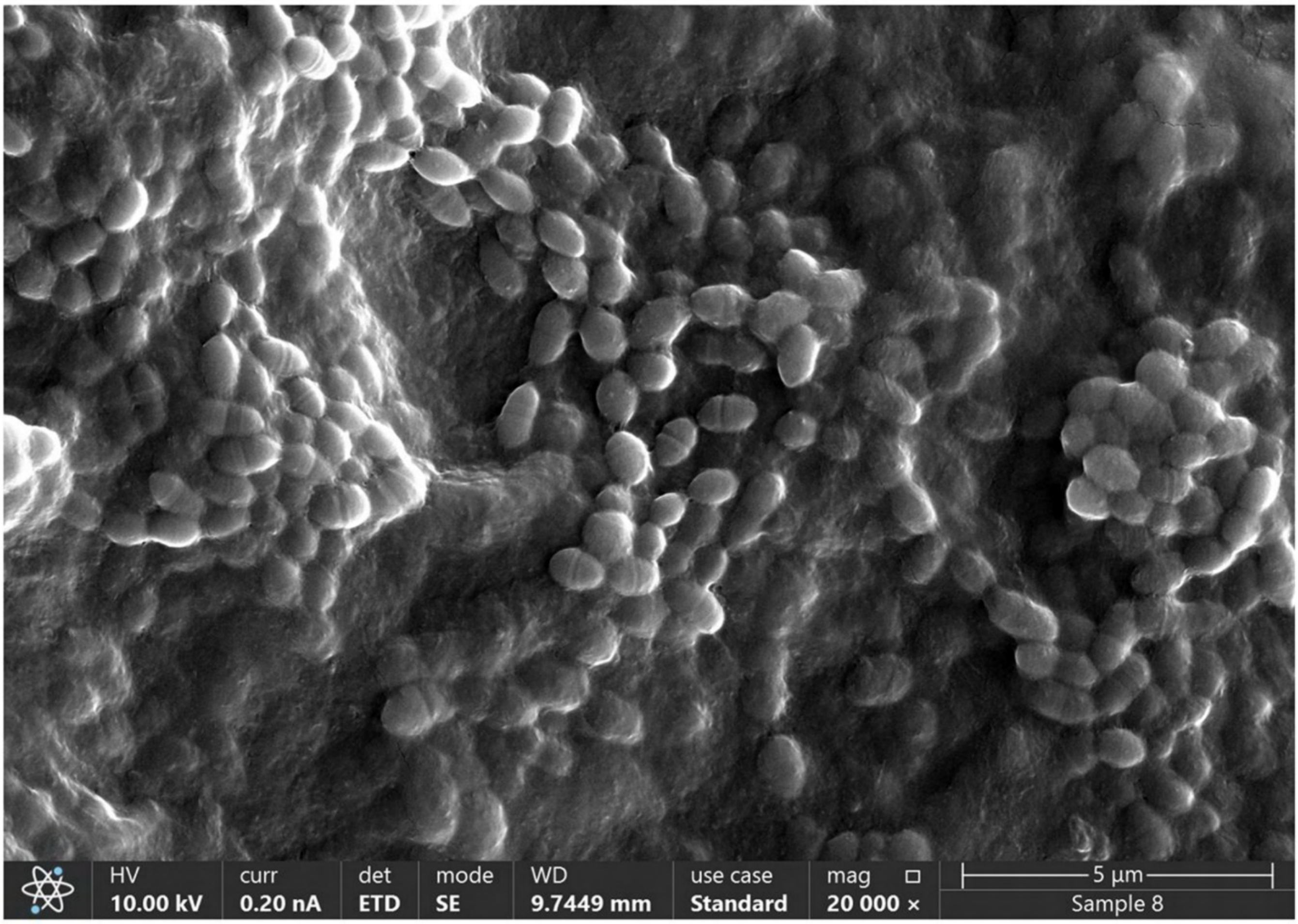

Supplementary Figure S6B : Uncropped HR-SEM images of biofilms formed by *S. mutans* in the presence of 6.25 µg/mL AA for 24 h. Magnification × 20,000.

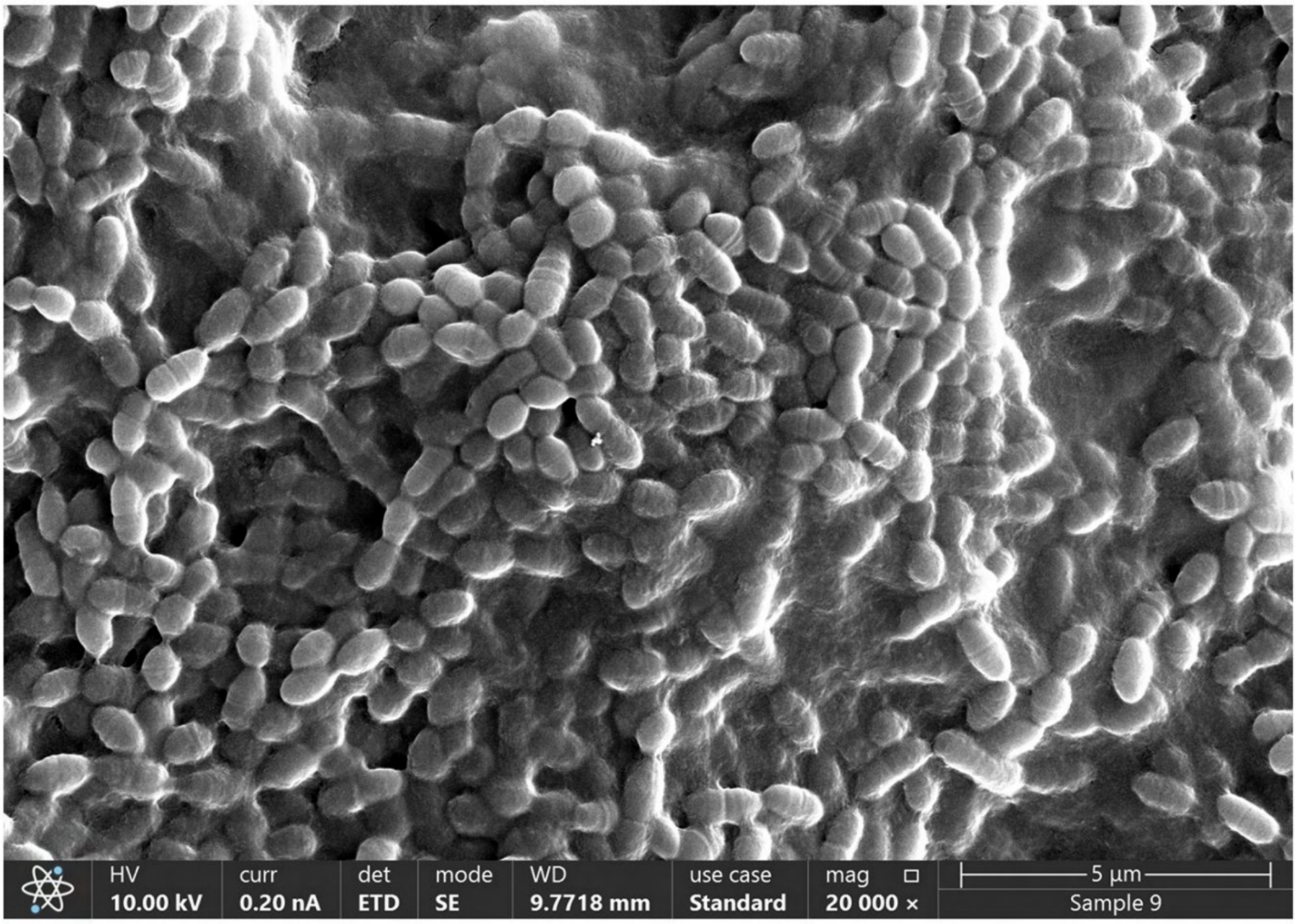

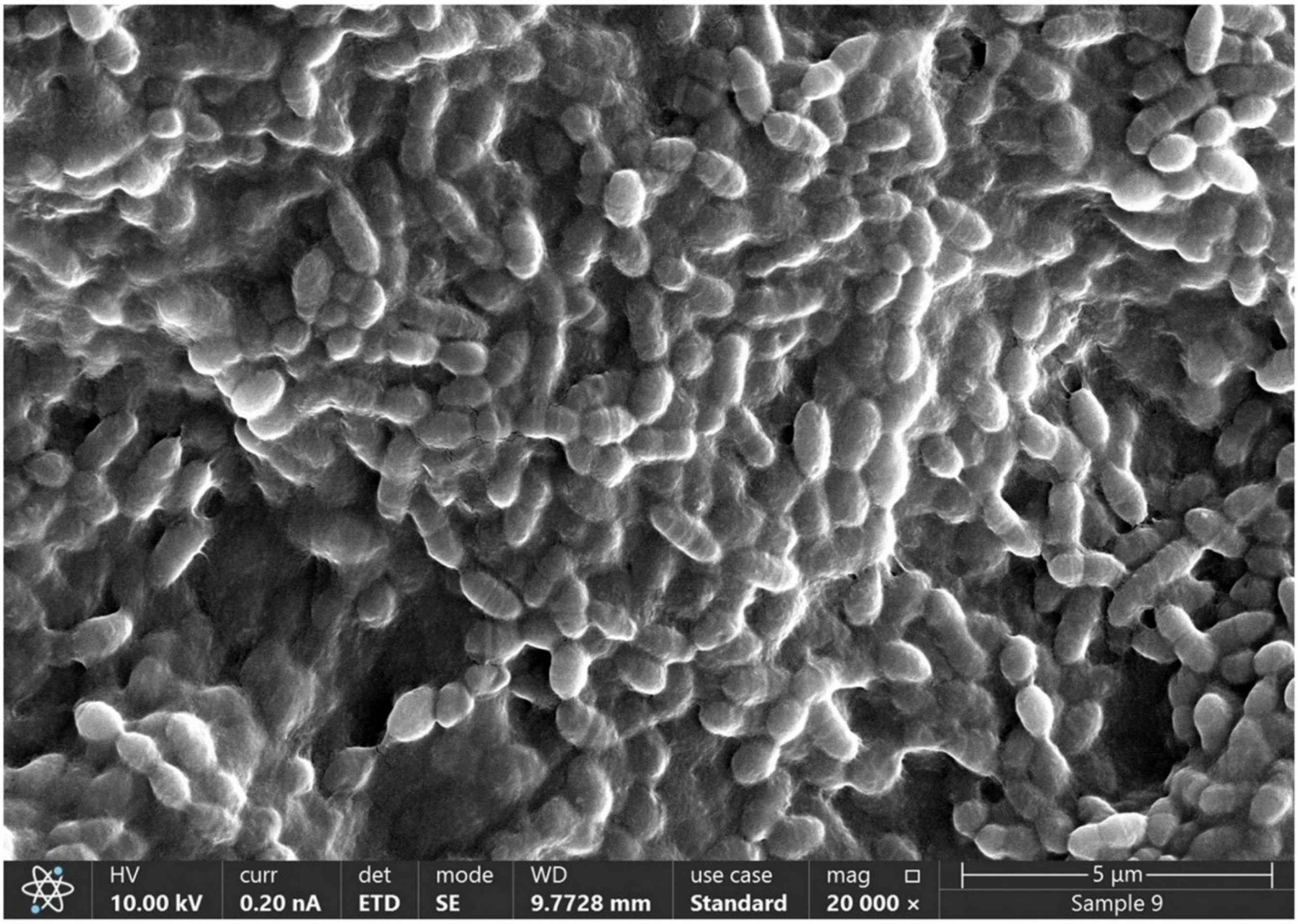

Supplementary Figure S6C : Uncropped HR-SEM images of biofilms formed by *S. mutans* in the presence of 12.5 µg/mL AA for 24 h. Magnification × 20,000.

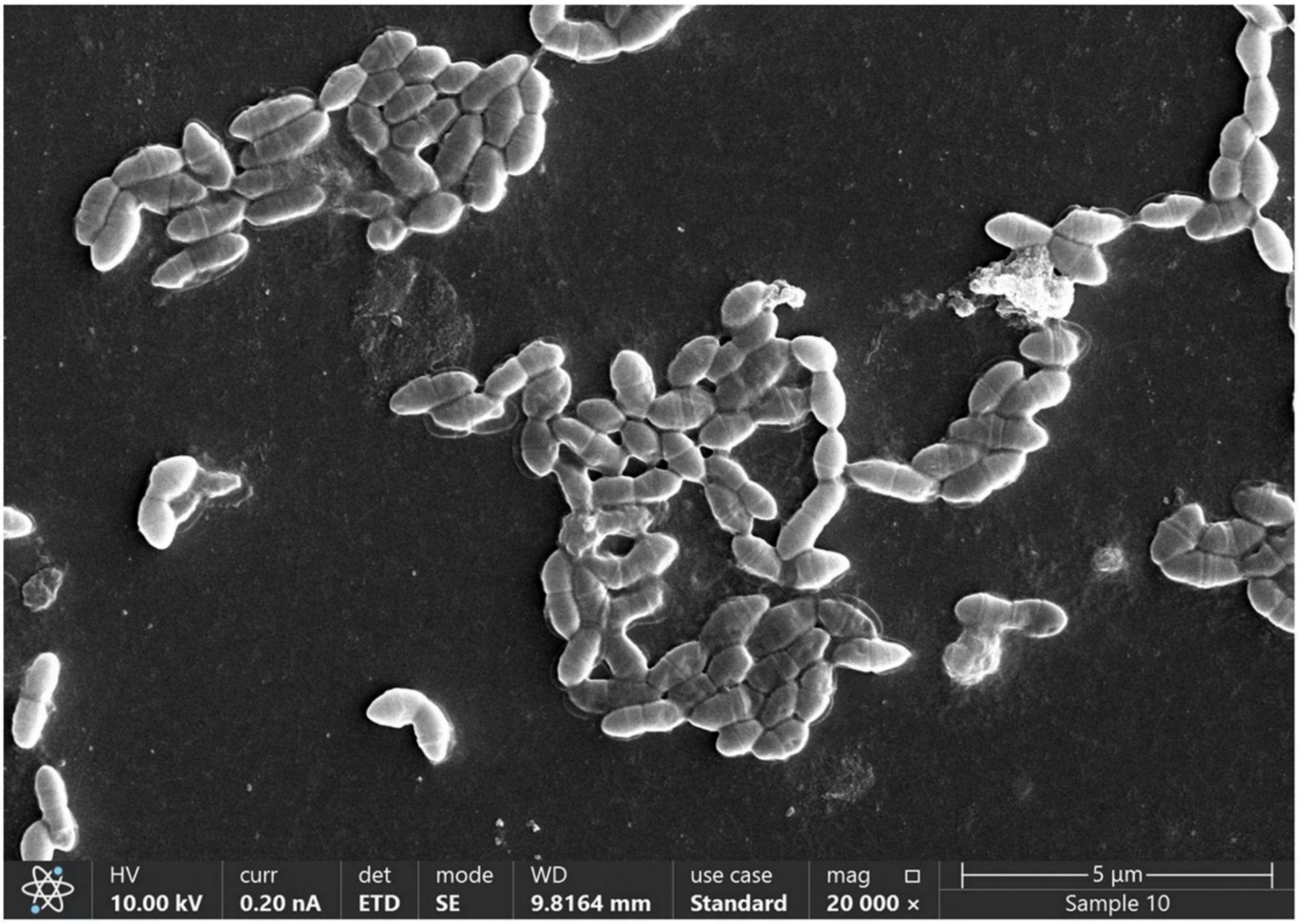

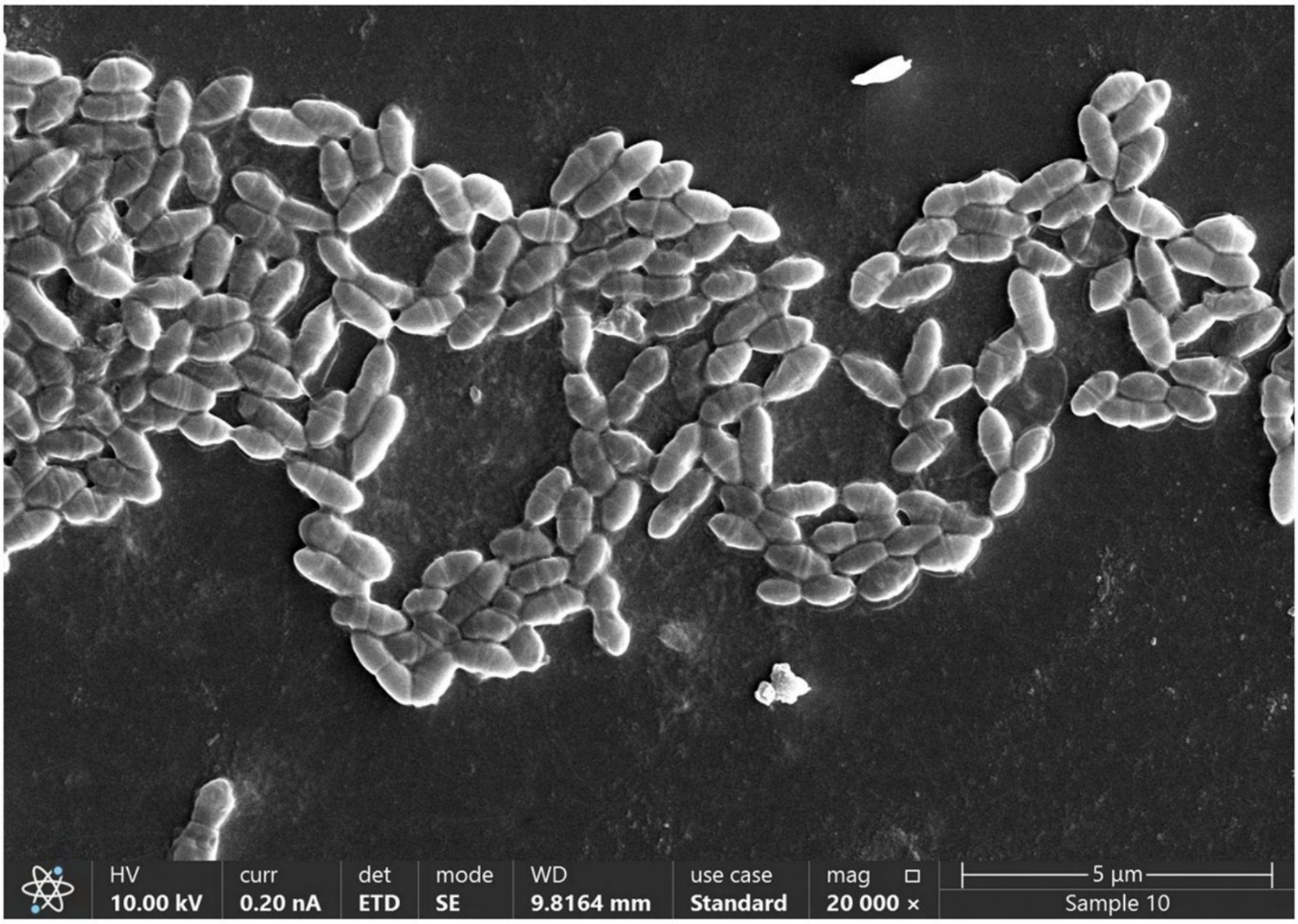

Supplementary Figure S6D : Uncropped HR-SEM images of biofilms formed by *S. mutans* in the presence of 25 µg/mL AA for 24 h. Magnification × 20,000.

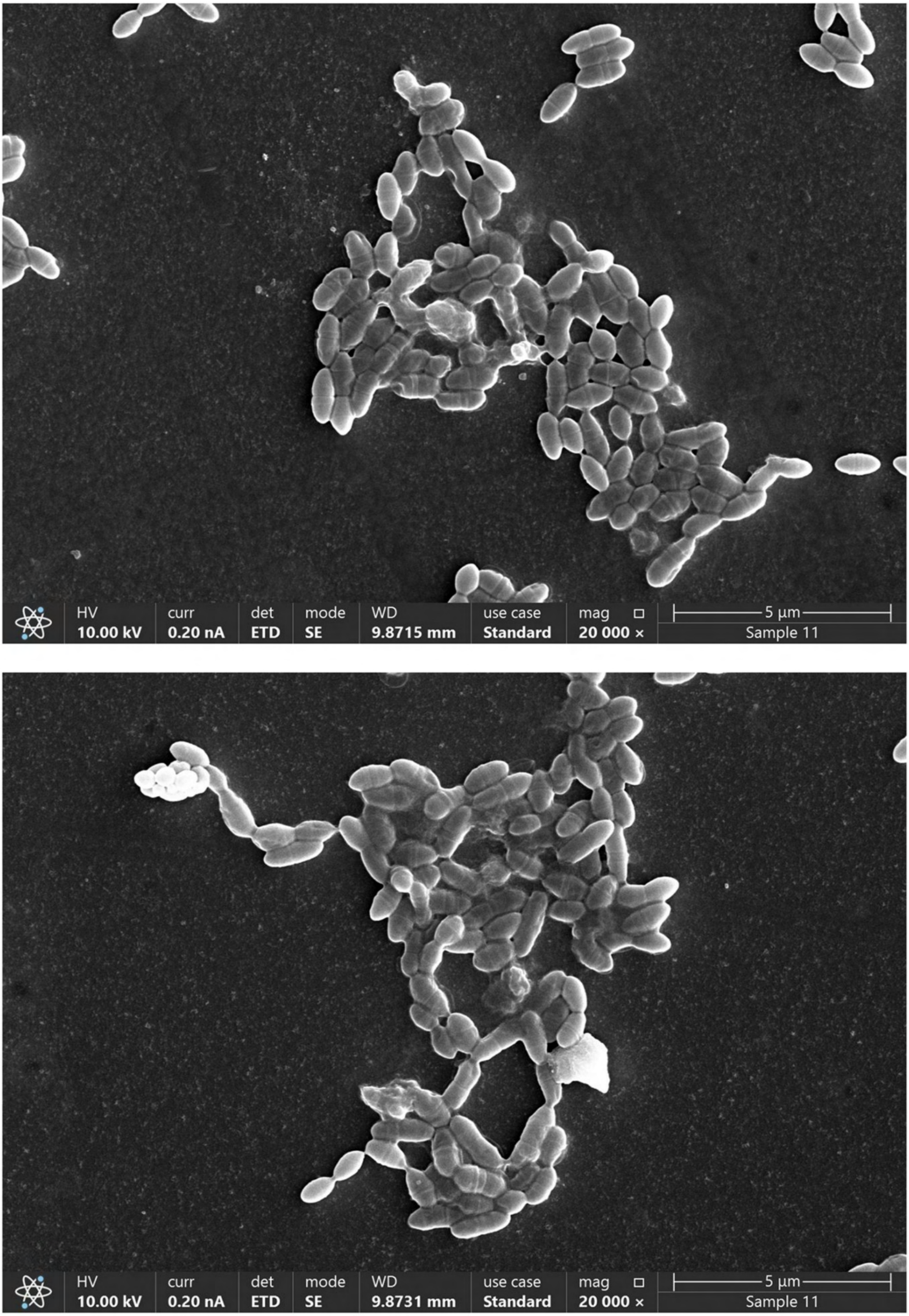

Supplementary Figure S6E : Uncropped HR-SEM images of biofilms formed by *S. mutans* in the presence of 50 µg/mL AA for 24 h. Magnification × 20,000.

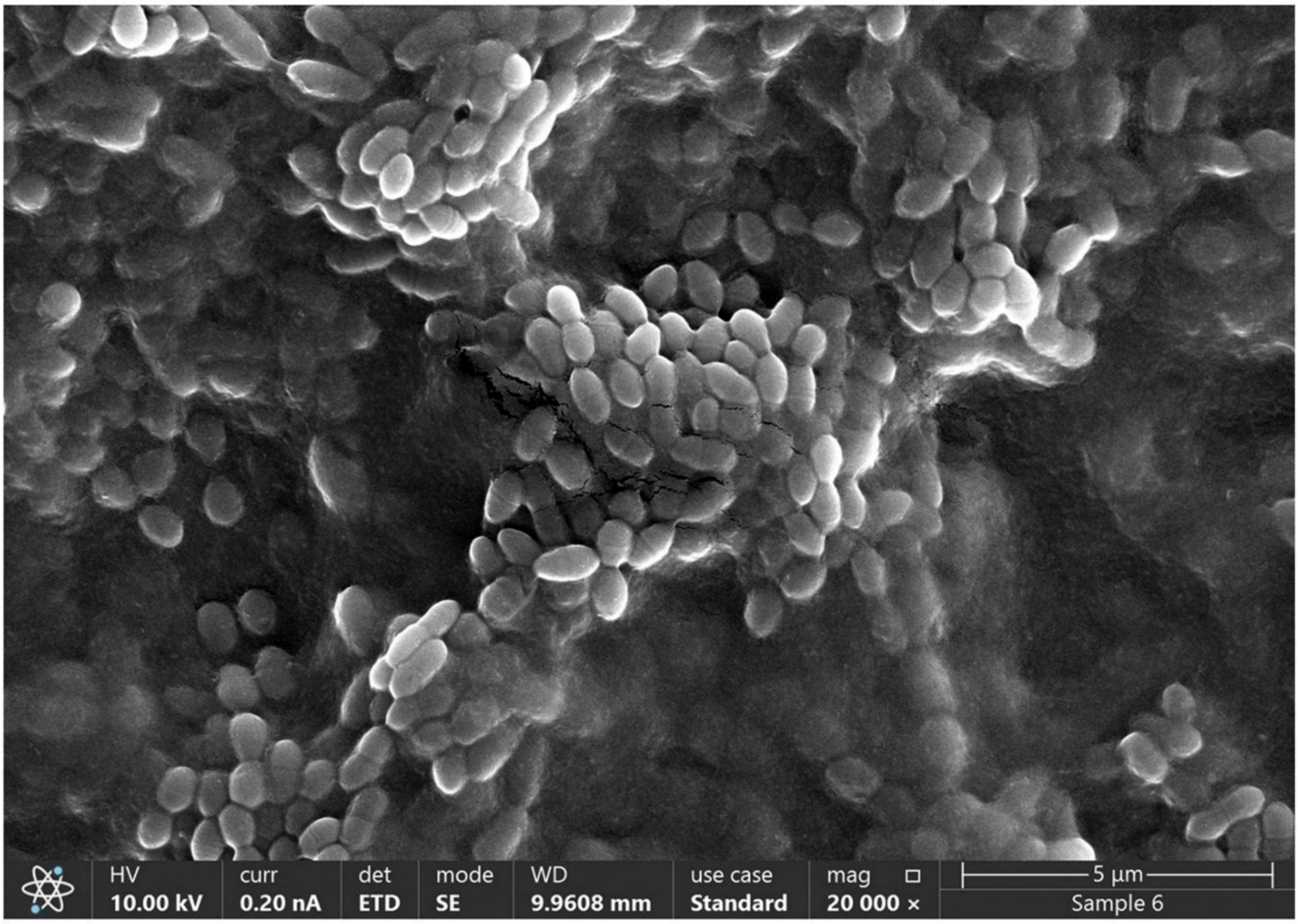

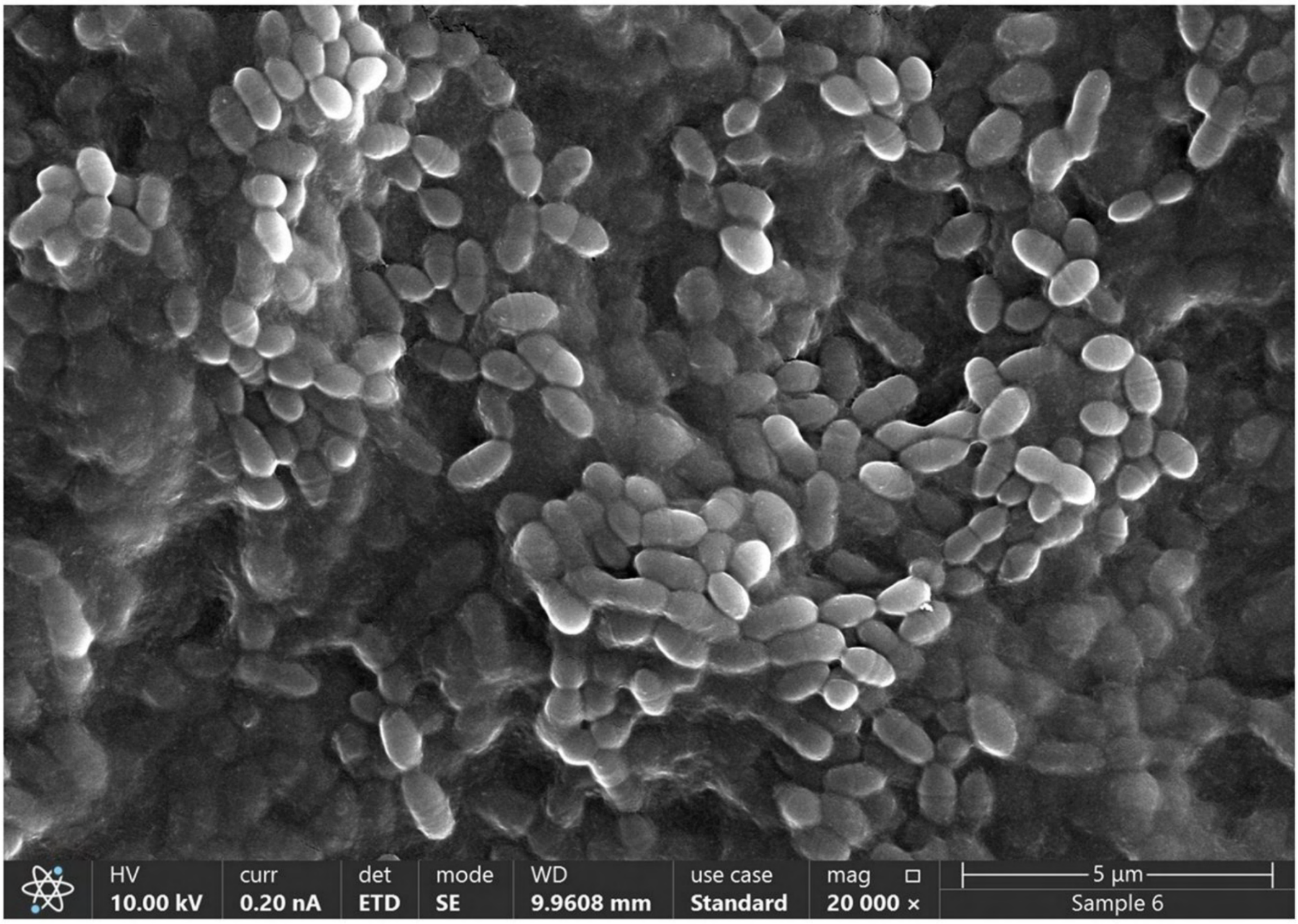

Supplementary Figure S6F : Uncropped HR-SEM images of biofilms formed by *S. mutans* in the presence of 0.1% ethanol for 24 h. Magnification × 20,000.

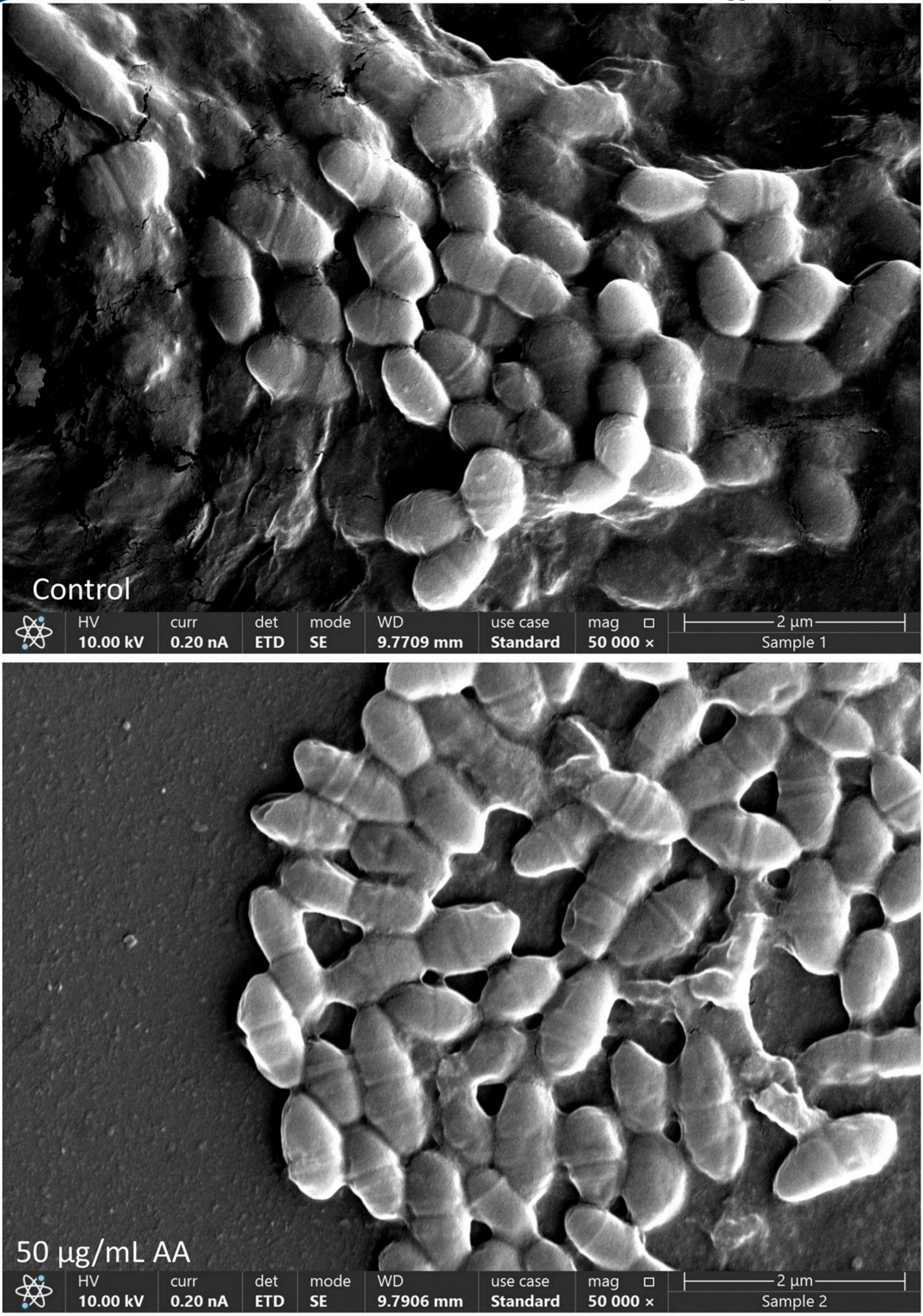

Supplementary Figure S7. Higher magnification (× 50,000) of *S. mutans* biofilm formed after 24 h in the absence (control) or presence of 50 µg/mL AA.

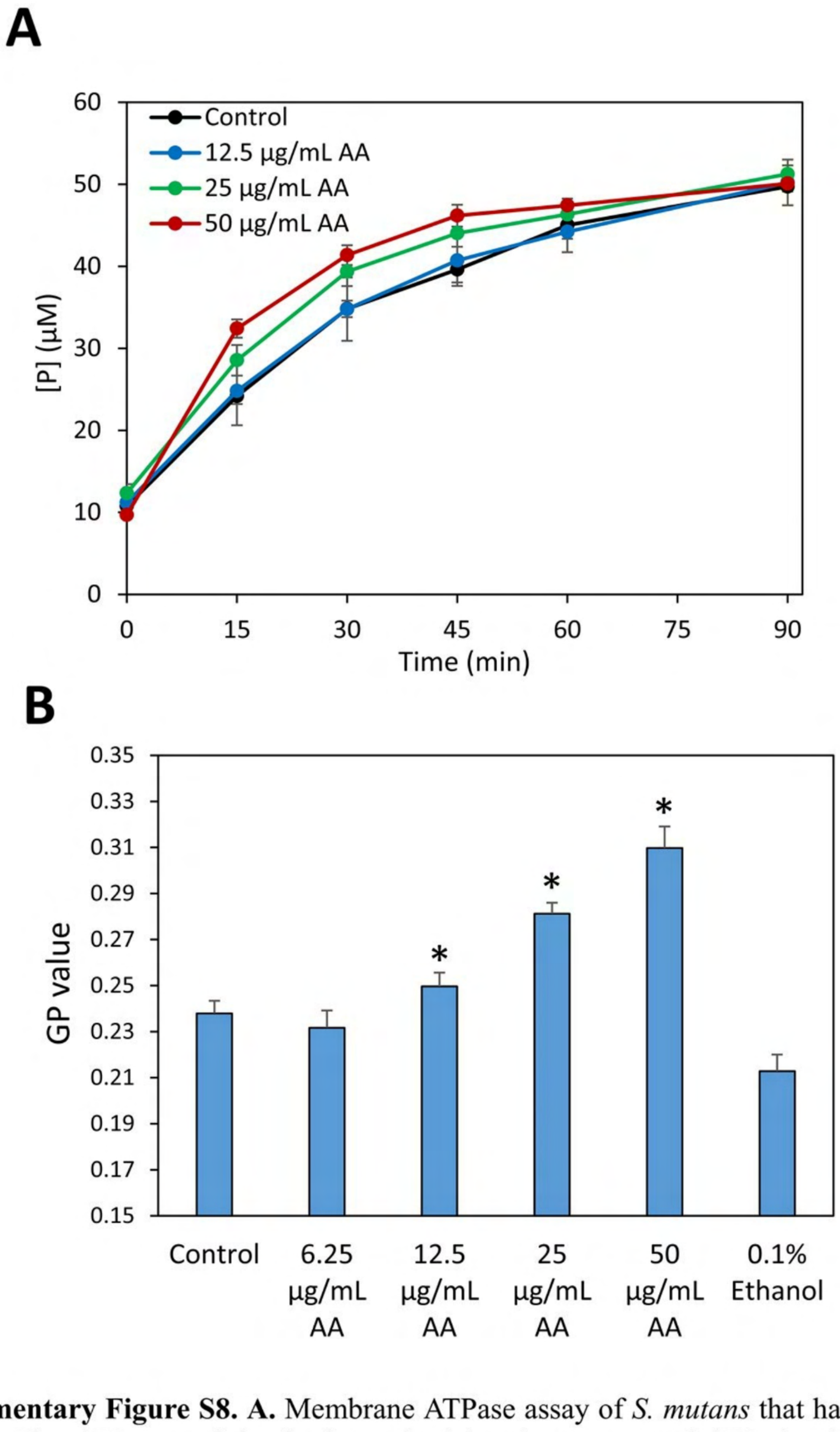

Supplementary Figure S8. A. Membrane ATPase assay of *S. mutans* that have been exposed to AA for 1 h. The ATPase activity is determined by the amount of ATP that has been converted to phosphate [P]. **B.** Changes in membrane fluidity after a 2 h incubation with increasing concentrations of AA as determined by Laurdan assay. GP = Generalized polarization = $(I_{440} - I_{490})/(I_{440} + I_{490})$, where I_{440} is the fluorescence intensity at 440 nm and I_{490} is the fluorescence intensity at 490 nm. Excitation was done with a wavelength of 350 nm.

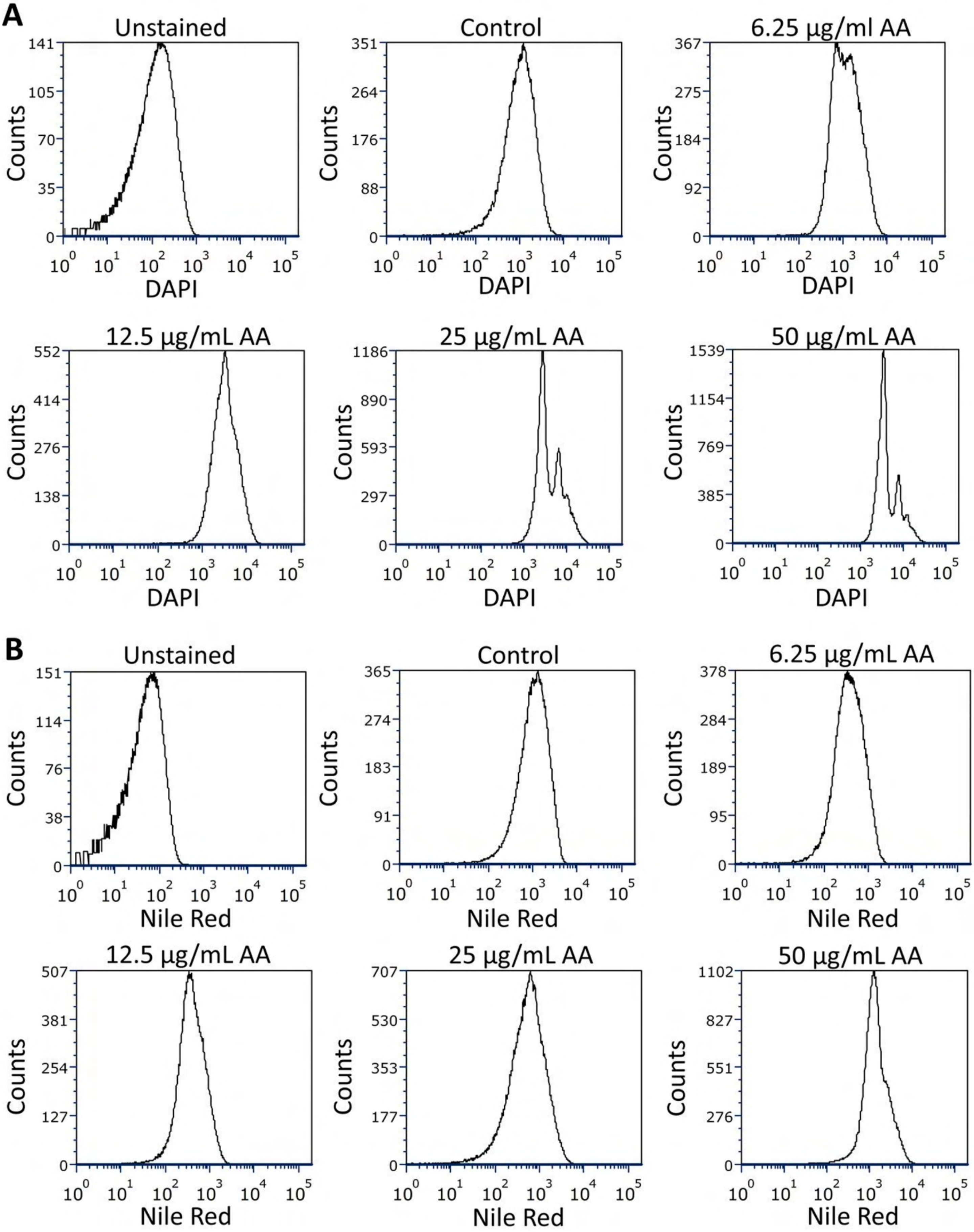

Supplementary Figure S9. A-B. Flow cytometry analysis of DAPI (A) and Nile Red (B) staining of live *S. mutans* that have been treated with various concentrations of AA for 2 h.

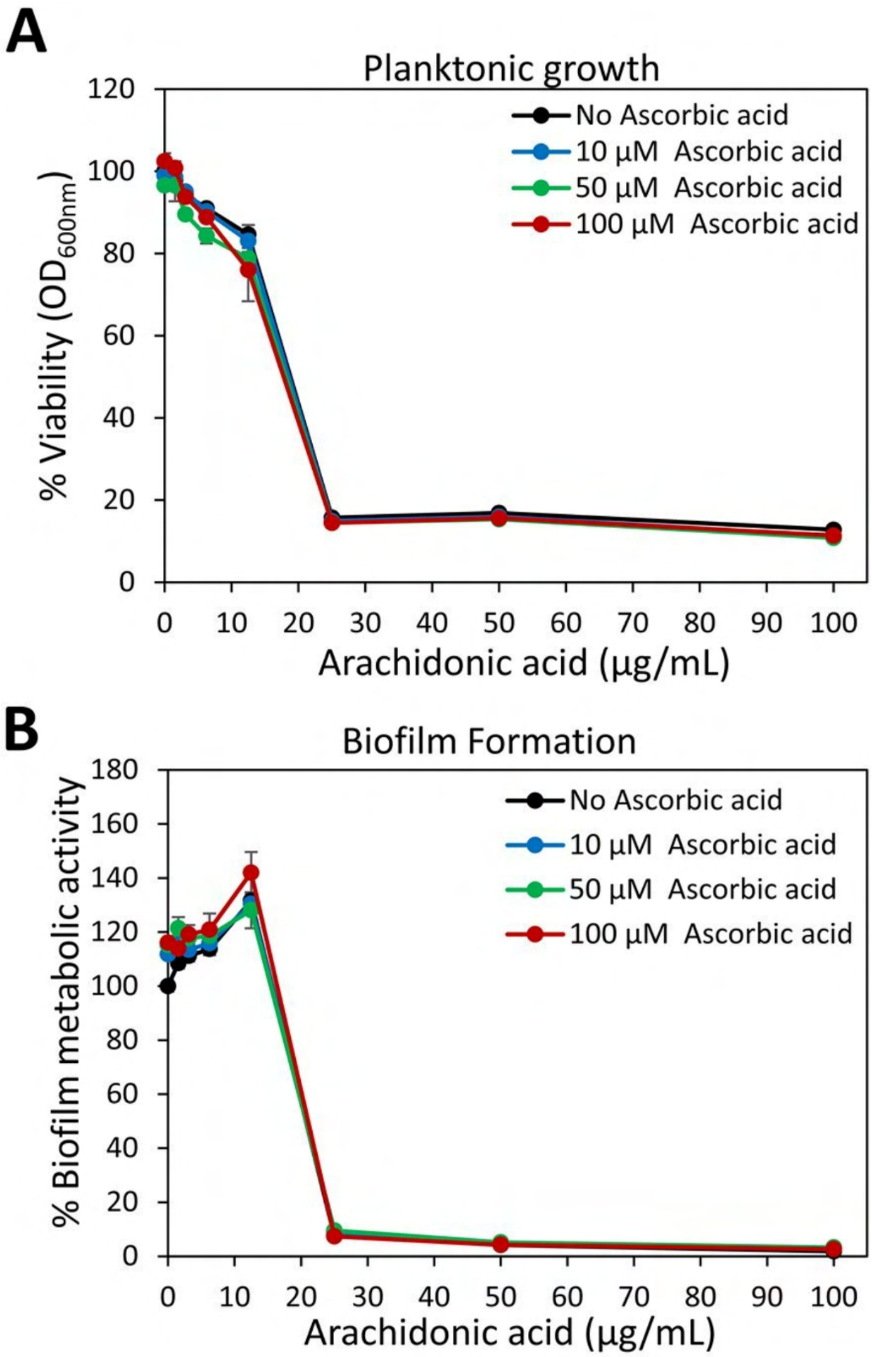

Supplementary Figure S10. **A-B.** Ascorbic acid at tested concentrations did not interfere with the anti-bacterial (**A**) and anti-biofilm (**B**) activities of arachidonic acid against *S. mutans*. *S. mutans* was incubated with ascorbic acid in the absence or presence of various concentrations of arachidonic acid for 24 h. The planktonic growth was analyzed by OD at 600 nm. The metabolic activity of biofilms formed in the presence of the various combinations of compounds was analyzed by MTT metabolic assay.

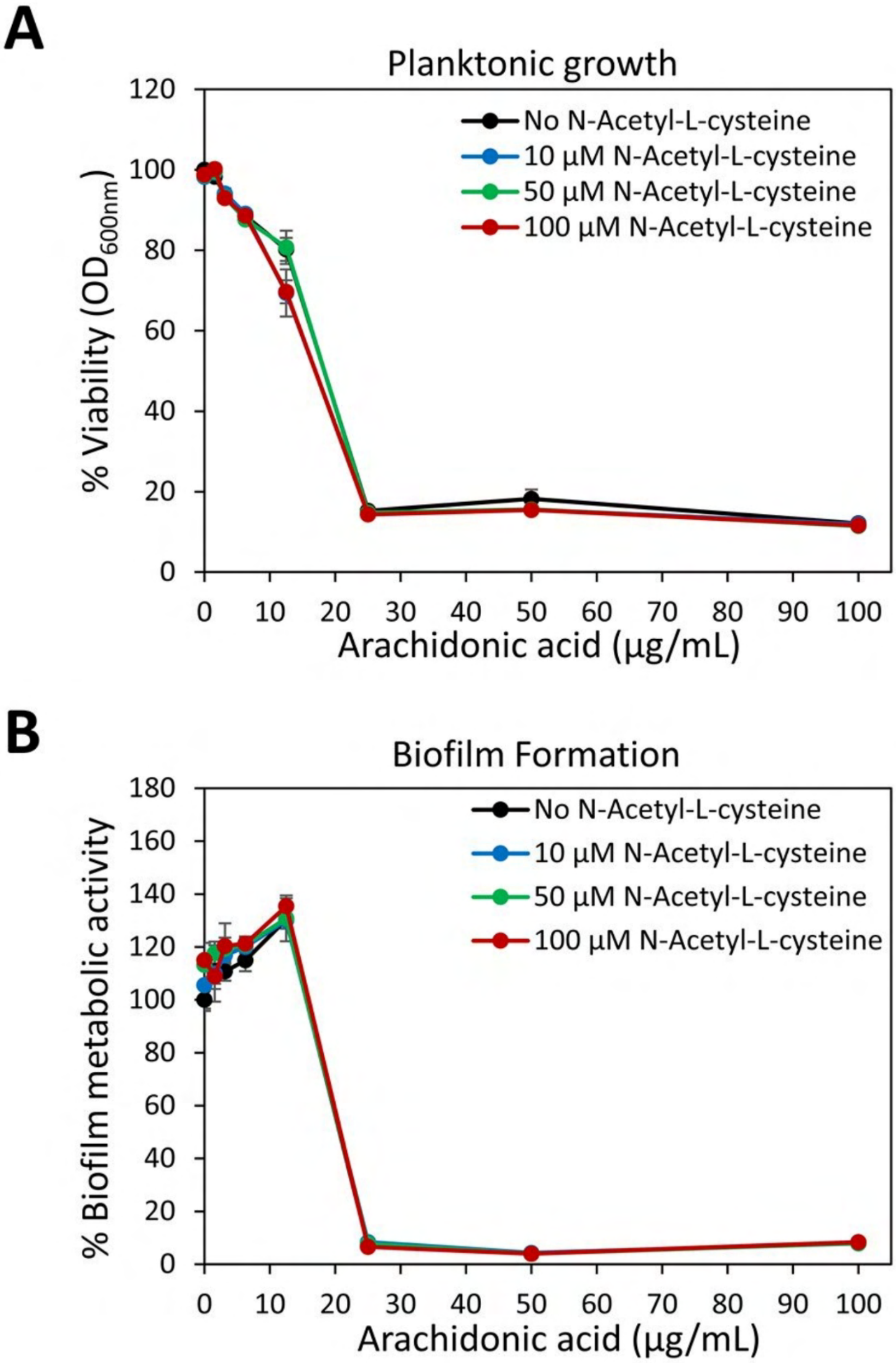

Supplementary Figure S11. A-B. N-Acetyl-L-cysteine at tested concentrations did not interfere with the anti-bacterial (**A**) and anti-biofilm (**B**) activities of arachidonic acid against *S. mutans*. *S. mutans* was incubated with N-acetyl-L-cysteine in the absence or presence of various concentrations of arachidonic acid for 24 h. The planktonic growth was analyzed by OD at 600nm. The metabolic activity of biofilms formed in the presence of the various combinations of compounds was analyzed by MTT metabolic assay.
